# Deep Brain Stimulation for Parkinson’s Disease with Early Motor Complications: A UK Cost-Effectiveness Analysis

**DOI:** 10.1371/journal.pone.0159340

**Published:** 2016-07-21

**Authors:** Tomasz Fundament, Paul R. Eldridge, Alexander L. Green, Alan L. Whone, Rod S. Taylor, Adrian C. Williams, W. M. Michael Schuepbach

**Affiliations:** 1 HTA Consulting, Krakow, Poland; 2 The Walton Centre NHS Foundation Trust and Liverpool University, Liverpool, United Kingdom; 3 Nuffield Department of Surgical Sciences, University of Oxford, Oxford, United Kingdom; 4 Bristol Brain Centre, Southmead Hospital, Bristol, United Kingdom; 5 University of Exeter Medical School, Exeter, United Kingdom; 6 University Hospitals Birmingham NHS Foundation Trust, Birmingham, United Kingdom; 7 Movement Disorders Center, Department of Neurology, Bern University Hospital and University of Bern, Bern, Switzerland; 8 Assistance Publique Hôpitaux de Paris, Centre d’Investigation Clinique 9503, Institut du Cerveau et de la Moelle épinière, Département de Neurologie, Université Pierre et Marie Curie–Paris 6 et INSERM, CHU Pitié-Salpêtrière, Paris, France; University of Ulm, GERMANY

## Abstract

**Background:**

Parkinson’s disease (PD) is a debilitating illness associated with considerable impairment of quality of life and substantial costs to health care systems. Deep brain stimulation (DBS) is an established surgical treatment option for some patients with advanced PD. The EARLYSTIM trial has recently demonstrated its clinical benefit also in patients with early motor complications. We sought to evaluate the cost-effectiveness of DBS, compared to best medical therapy (BMT), among PD patients with early onset of motor complications, from a United Kingdom (UK) payer perspective.

**Methods:**

We developed a Markov model to represent the progression of PD as rated using the Unified Parkinson's Disease Rating Scale (UPDRS) over time in patients with early PD. Evidence sources were a systematic review of clinical evidence; data from the EARLYSTIM study; and a UK Clinical Practice Research Datalink (CPRD) dataset including DBS patients. A mapping algorithm was developed to generate utility values based on UPDRS data for each intervention. The cost-effectiveness was expressed as the incremental cost per quality-adjusted life-year (QALY). One-way and probabilistic sensitivity analyses were undertaken to explore the effect of parameter uncertainty.

**Results:**

Over a 15-year time horizon, DBS was predicted to lead to additional mean cost per patient of £26,799 compared with BMT (£73,077/patient versus £46,278/patient) and an additional mean 1.35 QALYs (6.69 QALYs versus 5.35 QALYs), resulting in an incremental cost-effectiveness ratio of £19,887 per QALY gained with a 99% probability of DBS being cost-effective at a threshold of £30,000/QALY. One-way sensitivity analyses suggested that the results were not significantly impacted by plausible changes in the input parameter values.

**Conclusion:**

These results indicate that DBS is a cost-effective intervention in PD patients with early motor complications when compared with existing interventions, offering additional health benefits at acceptable incremental cost. This supports the extended use of DBS among patients with early onset of motor complications.

## Introduction

Parkinson’s disease (PD) is a chronic progressive neurodegenerative disorder involving dopaminergic neurons, which results in a lack of the neurotransmitter dopamine. Clinically the hallmark of PD is a movement disorder with bradykinesia, rigidity and often rest tremor, although non-motor signs and symptoms are also common [1,2]. A recent-meta-analysis concluded that the worldwide prevalence of PD is around 315 cases per 100,000 population [3]. During early stages of the disease, patients are effectively treated with oral medication such as levodopa; however, over time, medication-induced motor complications such as unpredictable fluctuations in motor symptoms and abnormal involuntary movements (dyskinesias) develop [4]. Progressively, the disease leads to increasingly severe motor signs, worsening of medication-related complications and a decrease in the time between ‘off’ periods when symptoms are not well-controlled. In advanced stages axial motor signs such as impaired balance and gait that respond less favourably to levodopa or DBS become more prevalent and non-motor, especially psychiatric and cognitive problems cause significant loss of quality of life [4–8]. Thus, there is a time window between the occurrence of levodopa-induced motor complications and the development of levodopa-resistant symptoms of PD when DBS can potentially improve the patients’ condition.

PD imposes a significant burden upon patients’ health-related quality of life [9–15], and creates a major economic burden for health care systems, driven mainly by hospitalisations and medication [9,16–20]. A broader socio-economic impact also occurs due to lost income owing to a reduced capacity to work, early retirement and institutional or unpaid care provided by patients’ relatives [17,18,21–27]. One European study has shown that a one-unit increase on the dyskinesia severity scale (Part IVa of the Unified Parkinson’s Disease Rating Scale (UPDRS)) results in additional mean combined medical and non-medical cost of €737 per patient over a 6-month period [21]. When indirect costs are included, this cost increases threefold [27]. For patients who in principle respond well to dopaminergic medication but whose benefit from oral medication is hampered by motor complications (fluctuations and dyskinesia), treatment options include deep brain stimulation (DBS), a surgical treatment which involves the implantation of a device for electrical stimulation of precise areas in the brain. Modulation of the activity of specific target structures in the brain results in improvement of certain parkinsonian motor signs. Current guidelines in the United Kingdom (UK) from the National Institute for Health and Care Excellence (NICE) recommend the use of DBS. These guidelines are currently being updated and will consider all relevant interventions including DBS [28].

Randomised controlled trial (level I) evidence support the use of DBS in advanced PD [29–31]. Previous economic studies have concluded that DBS is a cost-effective intervention, when compared with best medical therapy (BMT) among patients with advanced PD [32–35]. Furthermore, recent clinical evidence (the EARLYSTIM trial) has demonstrated that DBS is also effective in PD patients earlier in the disease course, with recent onset motor complications [36,37]. Significant improvements were observed compared with BMT in motor disability, activities of daily living, levodopa-induced motor complications and time with good mobility [36].

This study sought to assess the cost effectiveness of DBS versus BMT in treating PD from early onset of motor complications from a UK payer (National Health Service) perspective, employing a novel approach of associating UPDRS subscales with health-related quality of life to capture the multi-faceted aspects of PD.

## Materials and Methods

This economic modelling study was undertaken and reported in accord with the International Society for Pharmacoeconomics and Outcomes Research (ISPOR) best practice modelling guidelines [38].

### Overview of the economic model

We developed a Markov (state-transition) model to calculate the costs and health outcomes (in terms of life-years and quality-adjusted life-years [QALYs]) associated with a range of interventions for PD patients. Specifically, the model considered two treatment options:

- BMT: BMT alone
- DBS: DBS in combination with BMT.

**Fig 1:**
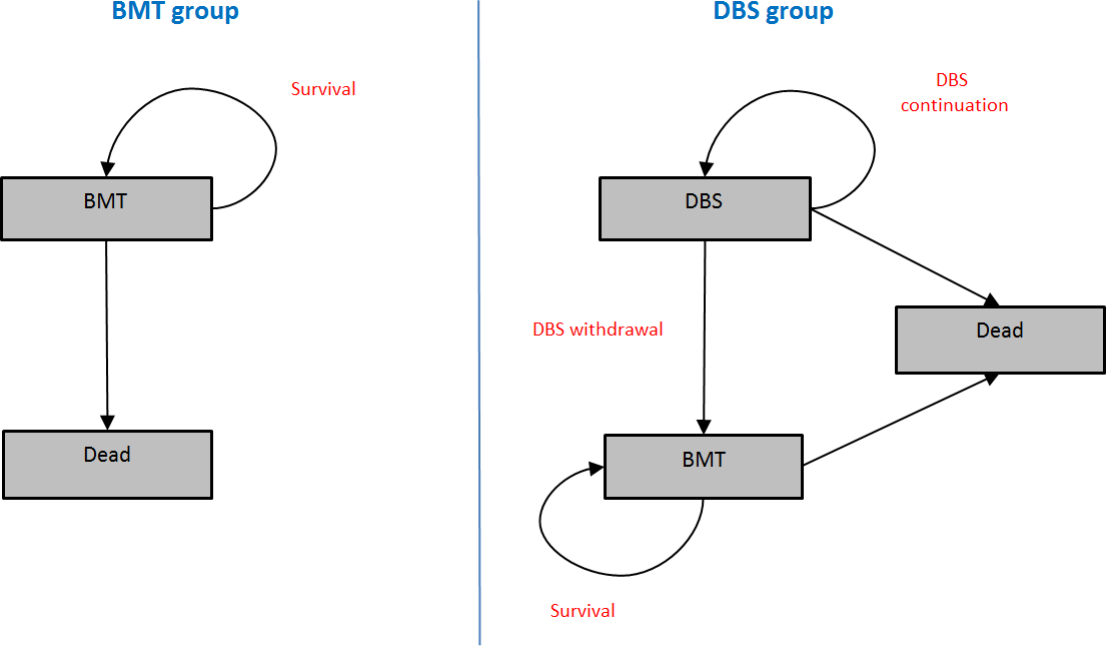
Model diagram BMT = best medical therapy; DBS = Deep brain stimulation.

Baseline characteristics for patients for both treatment options were based upon data from the EARLYSTIM study [36], i.e. all patients were assumed to have PD with early motor complications at model entry (i.e. those with motor fluctuations or dyskinesias present for 3 years or less). The model was developed in Microsoft Excel (Microsoft Corporation, Redmond, MA, USA), and in the base-case analysis a 15-year horizon was used to capture long-term results and progression to more advanced disease stages. A lifetime horizon in the base-case analyses was not considered appropriate given the uncertainty in long-term outcomes for patients on the therapies evaluated. A one-year cycle length was used for transitions between health states, and a half-cycle correction was applied to reflect the fact that patients move between states, on average, halfway through a cycle [39]. Health states were based around treatment interventions (see Fig 1), and for each treatment, disease progression was modelled according to changes in the UPDRS domain scores (Parts I to IV). The changes in UPDRS domain scores were recorded over time within the model, but were not explicitly used to derive health states. Health-related quality of life was accounted for using an existing mapping algorithm (in the short-term), and via the development of a new algorithm to link UPDRS scores to the Euroqol-5D (EQ-5D) in the long-term (see below). Costs and QALYs were both discounted at 3.5% per year, according to NICE methods guidance [40]. A systematic literature review was undertaken to identify relevant clinical data for each intervention; unit cost data were sourced from device price lists, national drug prices, hospital payment tariffs and social care cost data. Input was sought from a panel of clinical experts (PE, AG, AW, MS, AW) to ensure appropriate use of the data and for validation of the model structure and assumptions. A full table of input parameter values can be found in the Supplementary Information (S1 Table).

### Model structure and treatment comparisons

Fig 1 shows the model structure and health states for each intervention.

DBS patients would continue therapy until withdrawal, after which they would continue with BMT until the end of the model horizon or until death. No further interventions were modelled for patients withdrawing from DBS.

### Clinical data

A systematic literature review (including searches of PubMed, Embase, and The Cochrane Library) was undertaken to identify relevant short- and long-term clinical outcomes data for PD patients, and for each of interventions considered in the analysis. The systematic review considered studies among adult patients with PD which reported mean UPDRS scores at specified time points in the ‘ON’ state (i.e. on medication) (details of the systematic review are available in the supplementary information).

Disease progression and treatment effectiveness were modelled in terms of changes in UPDRS scores (Parts I to IV) over time for each intervention. Baseline characteristics of patients (in terms of mean UPDRS scores on each domain, and with a mean age of 52 years) in the model were based on pooled data from the DBS and BMT treatment groups of the EARLYSTIM study [36]. In the DBS and BMT treatment groups of the EARLYSTIM study, UPDRS domain scores were collected at treatment initiation and at 5, 12 and 24 months, which were used to calculate the percentage change from baseline in each domain score.

Disease progression beyond two years was modelled in a uniform way across all treatments, due to a lack of consistent long-term data on UPDRS outcomes for each intervention. Each UPDRS domain was modelled using data pooled from various studies, since no studies reported all domains consistently. Data from studies with a BMT treatment group were used to model the annual progression rate of UPDRS Parts I, II and IV [5,36,41–44]; a long-term study of DBS was used to represent progression of UPDRS III [45]. These rates were applied to both treatment options after two years, with the exception of UPDRS Part IV score. Clinical evidence suggests that UPDRS Part IV improvements may be more long-term for DBS patients [45–52]; in particular, two studies have demonstrated UPDRS IV scores at 8 years which are significantly better than baseline scores and not significantly different from corresponding scores at one year [50,51]. On the basis of this evidence, the clinical expert panel advised an extension of DBS response for this outcome; thus, the model held the two-year Part IV score constant for a further eight years, after which progression occurred at the same rate as for BMT. Alternative assumptions regarding the long-term effect of DBS upon UPDRS IV scores were tested in sensitivity analyses.

### Mortality data

Mortality was incorporated using a two-step approach. Firstly, an age- and gender-specific baseline mortality risk was applied using UK all-cause mortality rates [53], with patients entering the model at age 52.5 years, based on the EARLYSTIM study [36]. Whilst evidence suggests that in the early stages of PD, mortality is not significantly different to that of the general population [54–56], a recent review of mortality data in PD indicated that patients with advanced disease have a higher mortality risk [57]. Using data from studies reporting the relationship between UPDRS Part III and mortality, a 10-point increase in the UPDRS Part III score was associated with an increased mortality risk by applying a hazard ratio of 1.31 to the baseline risk [58,59,60]. This increased risk was applied to patients with a UPDRS Part III score of 15 or more, to reflect the impact only amongst patients with advanced disease.

### Adverse events

The model accounted for both treatment-specific and disease-related adverse events (AEs). Three AE types among DBS patients were included, i.e. surgery-related AEs (such as bleeding events and infections); hardware-related AEs (e.g. lead fractures and migrations); and other AEs such as worsening of mobility. Serious adverse event data from the EARLYSTIM study were used to inform the frequency of each event type [36]. Surgery-related AEs were modelled to only occur in the first two years following implantation; hardware-related and other AEs were associated with an ongoing risk. For BMT, EARLYSTIM study data were again used to model incidence of serious AEs, including worsening of mobility and motor fluctuations [36]. These risks were assumed to be constant over time.

PD progression is associated with increasing postural instability, leading to falls and in some cases serious injury to patients. To reflect the risk of falling, we pooled data from a series of studies to define the baseline proportion of patients falling per year as 42.78% [61–75]. An odds ratio of 1.07 for each point increase in UPDRS III score was then applied, derived from three studies of fall incidence [61,64,66]. Of these falls, 50.9% were assumed to require hospitalisation of the patient [62,65,72]. The clinical panel suggested that withdrawal from DBS is rare; therefore, we modelled DBS withdrawal only as a consequence of specific adverse events. Withdrawal probabilities in the other treatment groups were calculated based on studies reporting such data. Upon withdrawal from DBS patients were assumed to receive BMT until death or the end of the model horizon, with UPDRS scores reflecting this change of treatment.

### Quality of life data

UK guidelines for economic evaluations state that health outcomes should be expressed in terms of QALYs, with the EuroQol-5D (EQ-5D) being the preferred measure of health-related quality of life [40]. EQ-5D data were not collected in the EARLYSTIM study, and we therefore used a published algorithm to map from the 39-item Parkinson’s Disease Questionnaire (PDQ-39) data from EARLYSTIM to the EQ-5D [76]. The algorithm used a multinomial logistic regression approach to predict the spread of patients between the three levels of each of the five domains of the EQ-5D. These values were then used to derive a utility index for a UK population [77], with a utility weight calculated for DBS and BMT patients separately by averaging across the data from months 5 and 12 (for the year 1 utility) and months 12 and 24 (for the year 2 utility) in the EARLYSTIM study. These utility weights were applied in the first two years of the model.

Whilst studies have demonstrated a correlation between UPDRS Parts I and II and both the EQ-5D index and the PDQ-39 [78,79], existing algorithms do not fully capture this relationship [80]. For this reason we developed a new algorithm to apply after the first two years of the model for both treatment groups. An iterative process was used to identify a statistical model which could accurately predict the EQ-5D index from the explanatory variables available from the EARLYSTIM study (including UPDRS domain scores, and patient gender and age). Several model types were explored, including linear regression, beta regression, and finite mixtures of linear and beta regression with a range of link functions. A comparison of model fit was made using mean error, mean absolute error, the Bayesian information criterion and the Akaike information criterion. A beta regression approach with a log link function was considered the most appropriate, as it resulted in small errors, covered the full range of utility values possible with the EQ-5D and did not produce illogical results (e.g. worse UPDRS scores leading to higher utilities). The function is given below:

$$EQ-5D=1.59*e^{\left(0.01721*Male+0.001448*Age-0.0198*UPDRS\;I-0.00049*{\left(UPDRS\;II\right)}^{2}-0.0178*UPDRS\;IV-0.2468\right)}-0.594$$

Where ‘Male’ is set to one for males and zero for females.

The algorithm was subsequently applied to patients’ changing UPDRS scores over time in the model to predict variation in health-related quality of life for the remainder of the model.

### Cost and resource use data

The cost analysis was undertaken from a UK National Health Service perspective. The main cost groups included were: pre-surgery hospitalisation; device acquisition and implantation; drug acquisition (BMT); adverse event management (treatment-specific and generic events); general follow-up; treatment withdrawal; and device replacements. Hospital-related costs were based upon Payment by Results tariffs [81], drug costs were taken from the British National Formulary [82], and GP and nurse follow-up visits were taken from the Personal Social Sciences Research Unit [83].

For DBS, two pre-operative assessments were required to undertake tests on the patient and prepare them for the implantation procedure. A separate hospitalisation was then included at which the device, leads and extensions were implanted, with the costs of the full DBS system applied at this point (i.e. device, leads, extensions and patient programmer) [84]. The cost of a battery replacement was assumed to include the cost of a new device, plus a hospitalisation for the procedure, with a mean battery lifetime of 4.5 years used [85]. The costs of AE management were based on the frequency of serious AEs observed in the EARLYSTIM trial, with separate costs applied to surgery-related events, device-related events and other events observed in the study [81,86]. In some cases, more than one plausible payment tariff was identified, and in such instances the mean of the available tariffs was applied.

Given the range of drug options available for PD management and the lack of standardised drug protocols, we calculated drug use across treatment options for BMT using an analysis of data from the UK Clinical Practice Research Datalink (CPRD) [87]. Data were extracted on a total of 297 patients (270 on BMT and 27 on DBS) for the period April 1 2003 to March 31 2012, and included information on a total of 305 different PD drug formulations administered during this period. Dosing information for each patient was combined with drug unit costs from the British National Formulary [82], and the number of patients receiving each drug (from the CPRD) to calculate mean daily drug costs for each treatment group. The calculated drug cost per day for each treatment option was £4.16 (BMT) and £2.28 (DBS). These costs were assumed to be constant over time.

The costs of general follow-up were accounted for in each treatment group via regular neurology outpatient appointments. For patients receiving DBS, four visits were assumed in the first year of treatment to account for device programming and drug dose adjustments, with two visits assumed per year thereafter. Patients on BMT were assumed to require two visits per year for the duration of the model, except in the case of patients withdrawing from DBS, who were assumed to require four such visits in the first year after withdrawal. Home visits by a PD nurse were applied in both treatment groups with the same frequency as for the neurology outpatient visits.

PD-related falls were assumed to require hospitalisation in 50.9% of cases [62,64,72]. The unit costs of follow-up and falls were based on national tariffs and social service cost estimates [81,83]. Finally, the costs of any additional hospitalisations were included for each treatment option. The CPRD dataset of PD patients (364 patients, described above) was used to estimate the mean number of inpatient days per patient per year [87], which were adjusted for hospitalisations related to treatment and adverse events described above, and then multiplied by a cost per hospital day. The mean number of hospital days per patient was estimated as 5.97 (BMT) and 2.63 (DBS). According to expert clinical advice, these hospitalisations can be assumed to be mainly due to PD-related co-morbidities.

A full list of the unit costs used in the model is given in S1 Table in the Supporting Material.

### Data Analyses

A deterministic analysis was firstly undertaken, using the mean value of each parameter to calculate the total costs, life-years and QALYs over a 15-year horizon. To allow comparison of the cost-effectiveness of the two interventions, the incremental cost-effectiveness ratio (ICER) was calculated using the following formula (‘A’ and ‘B’ refer to the two interventions being compared):

$$ICER=\frac{{Costs}_{A}-{Costs}_{B}}{{QALYs}_{A}-{QALYs}_{B}}$$

To explore the effect of individual parameter uncertainty upon the cost-effectiveness results, extensive one-way sensitivity analyses were also undertaken, varying each parameter in turn within plausible ranges. Probabilistic sensitivity analysis (PSA) was performed to explore the joint effect of the uncertainty in all input parameter values. This involved assigning a statistical distribution to each parameter to reflect the uncertainty in its mean value. A range of distributions was used (including normal, beta, gamma and lognormal) according to the parameter type and any necessary restrictions on possible sampled values. One value was sampled from each parameter’s distribution and the model results re-calculated using these values; this process was repeated 10,000 times to provide a range of costs and QALYs for each intervention.

## Results

### Deterministic analysis

Table 1 shows the discounted results of the deterministic analysis, based on a 15-year time horizon.

**Table 1:** Deterministic model results (discounted).

Treatment	Mean cost per patient	Mean QALYs gained per patient	ICER (cost per QALY gained)
BMT	£46,278	5.35	-
DBS	£73,077	6.69	£19,887

QALY = quality-adjusted life-year; ICER = incremental cost-effectiveness ratio

**Fig 2:**
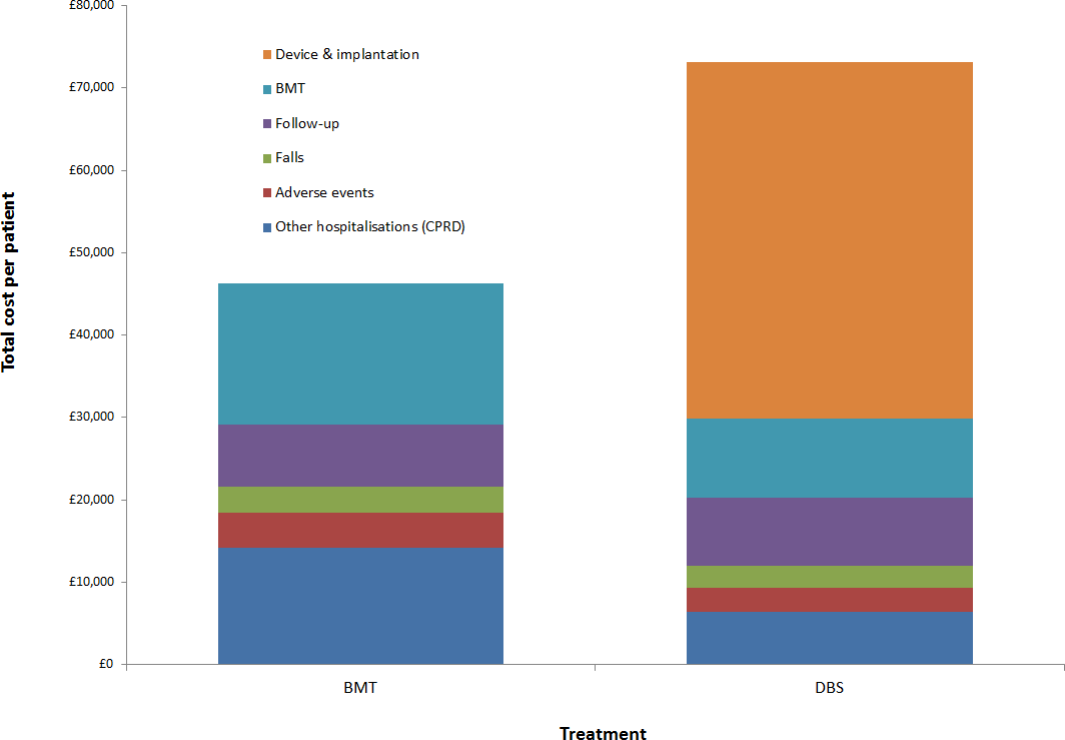
Cost breakdown by treatment.

DBS was predicted to lead to improved QALY outcomes and increased costs compared with BMT, leading to an ICER of £19,887 per QALY gained. In the DBS group, two-thirds of the total costs were related to device acquisition, implantation and replacement (see Fig 2).

**Fig 3:**
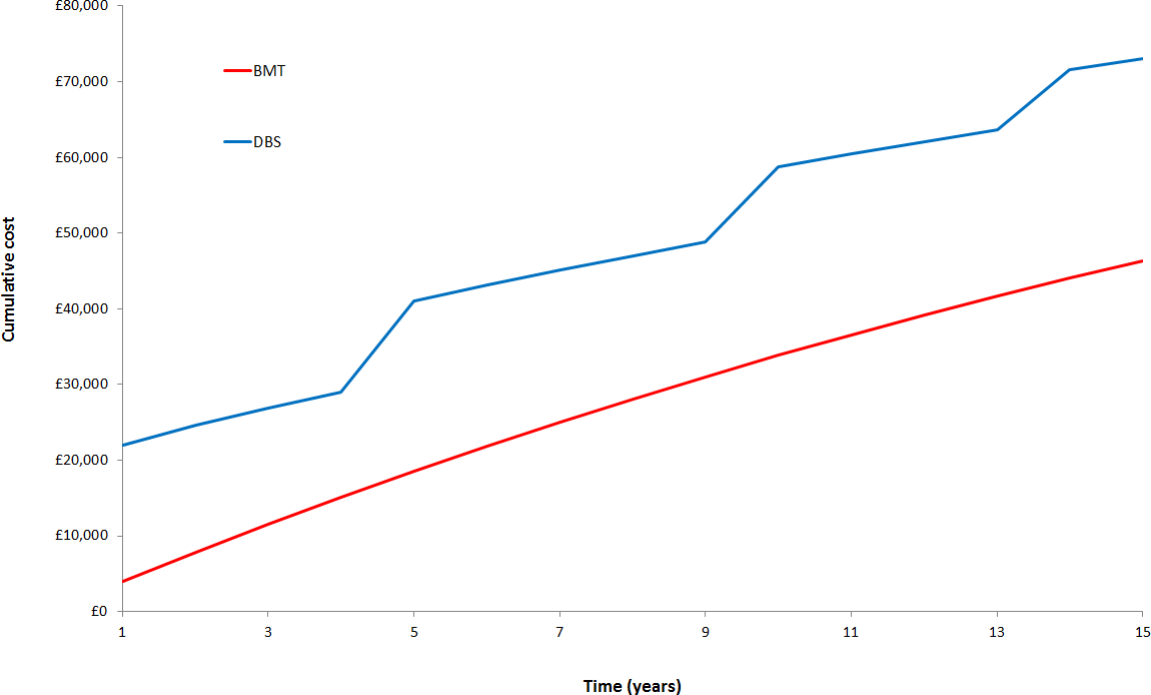
Cumulative discounted costs over 15 years.

**Fig 4:**
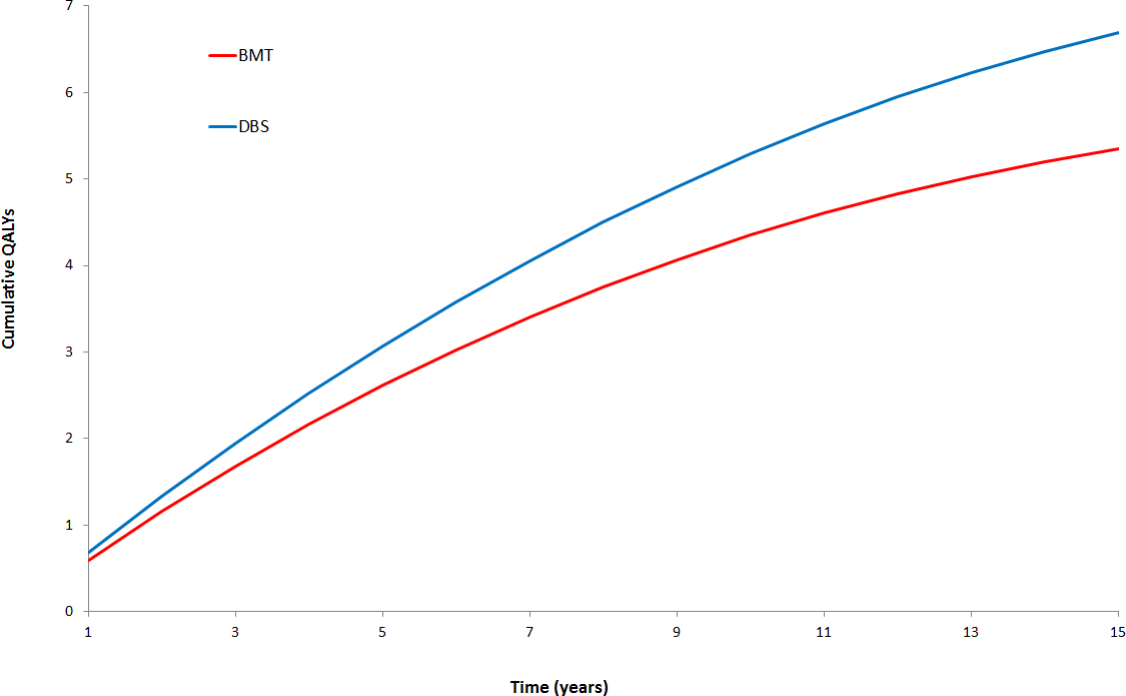
Cumulative discounted QALYs over 15 years.

Figs 3 and 4 show the cumulative costs and QALYs over the 15-year period for each intervention.

These results show a steady increase in the total costs and QALYs over time for each intervention. The timing of DBS battery replacements can be seen in Fig 3 at regular intervals.

### One-way sensitivity analysis

**Fig 5:**
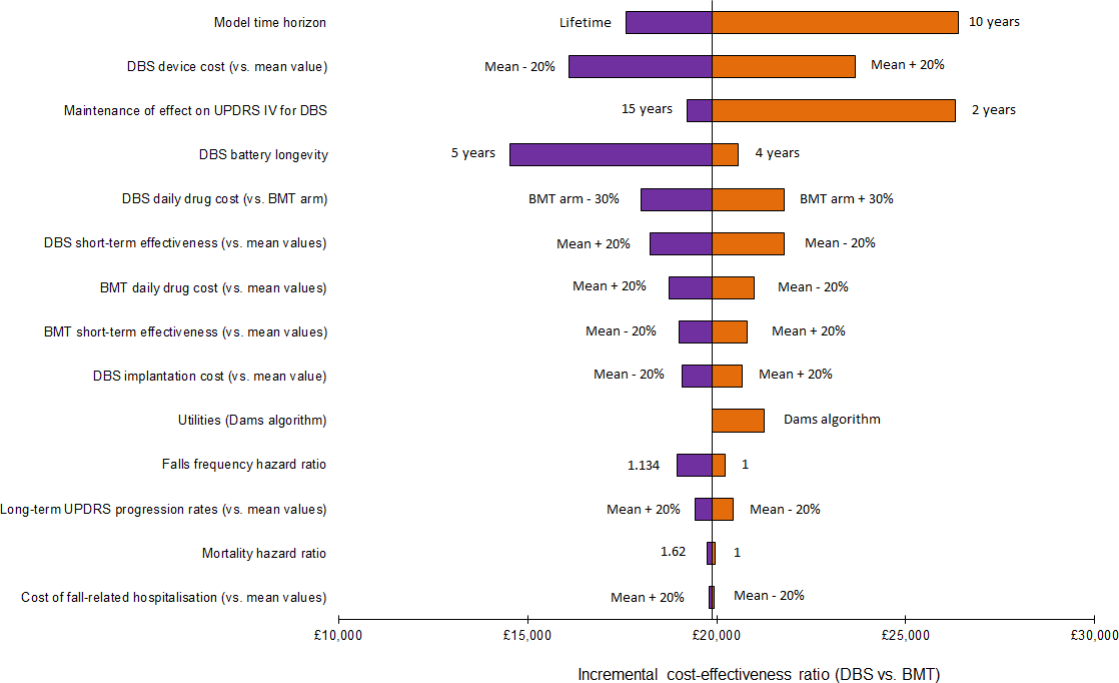
Tornado diagram of one-way sensitivity analysis for DBS versus BMT (plausible parameter ranges).

Fig 5 shows the tornado diagram for the comparison of DBS versus BMT. The variables whose uncertainty was most influential are shown towards the top of the diagram.

The key parameters identified by the one-way sensitivity analysis were: the model time horizon; the cost of the DBS system; the duration of the effect of DBS upon the UPDRS Part IV score (when this effect was assumed to last for 2 years, a QALY gain of 1.02 was observed for DBS patients, compared with 1.35 in the base-case); and the DBS battery longevity. In no scenario, however, was the computed ICER for the comparison of DBS versus BMT in excess of £30,000 per QALY gained.

### Probabilistic sensitivity analysis

**Fig 6:**
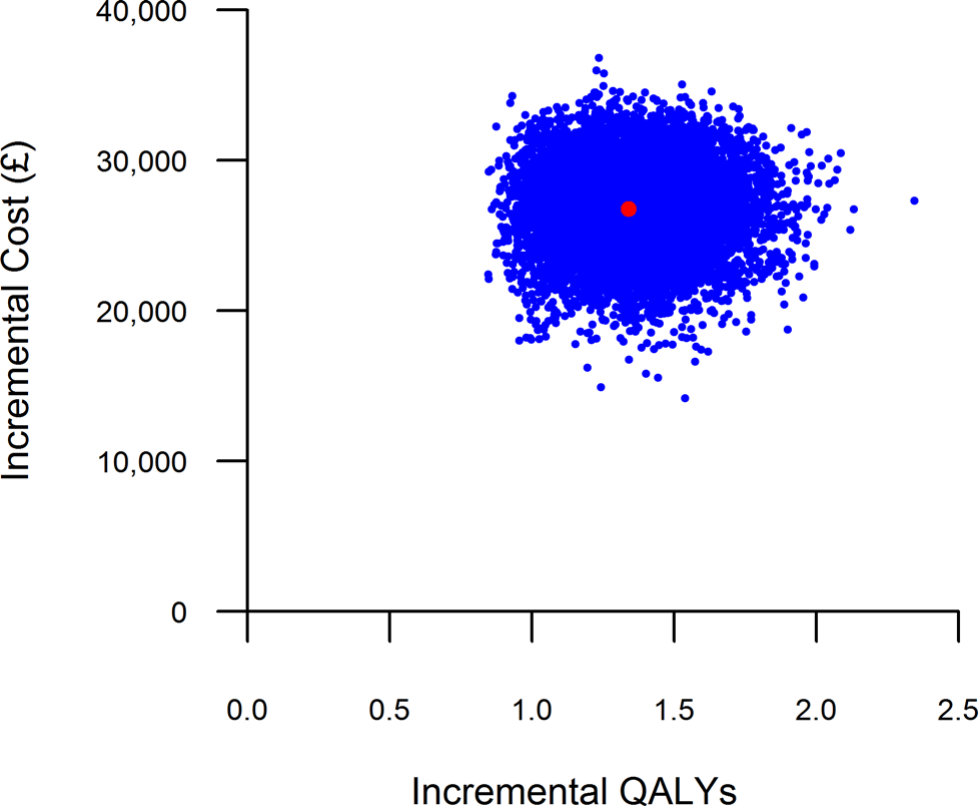
Scatter plot of probabilistic results (15-year horizon). The red dot represents the deterministic result.

**Fig 7:**
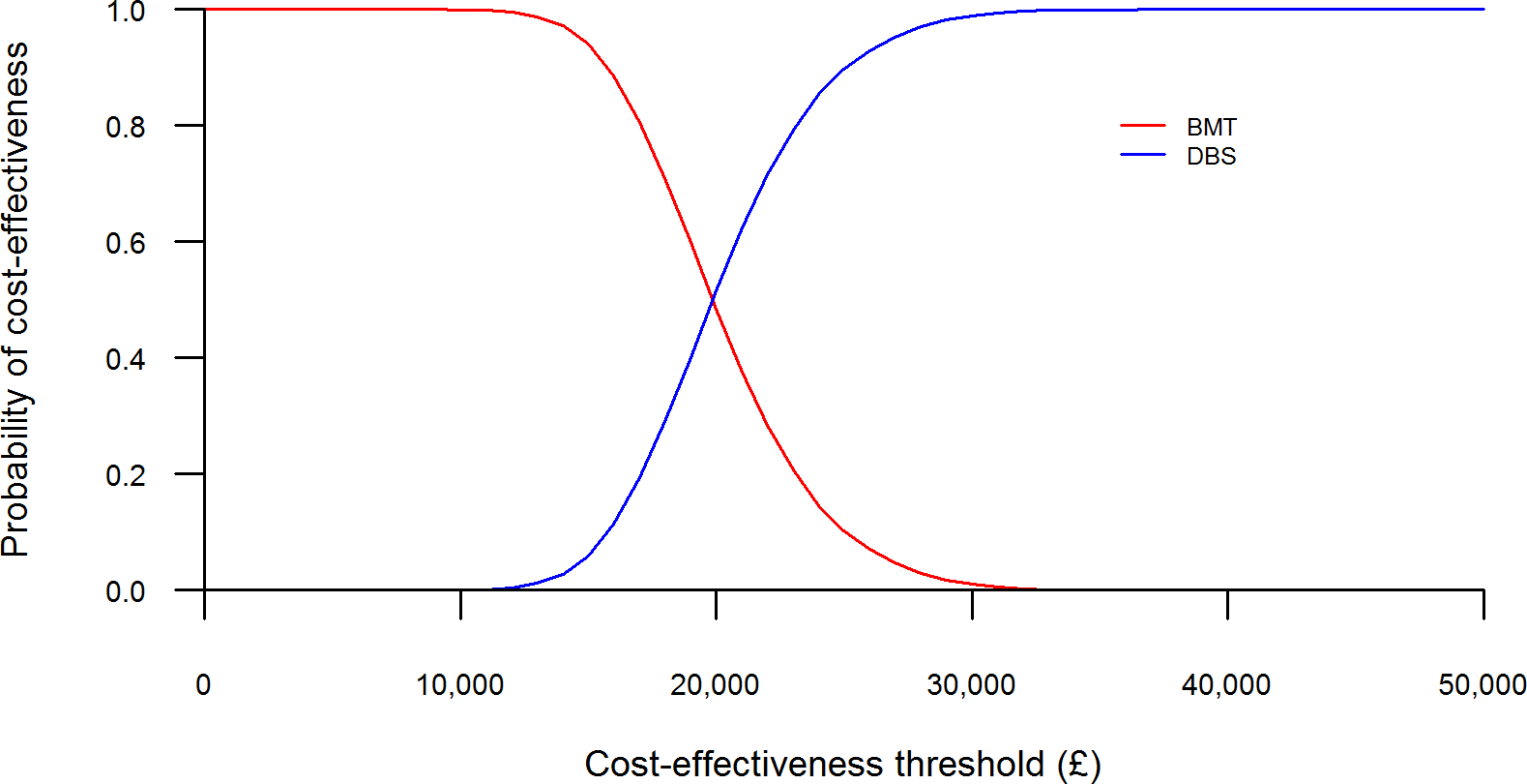
Cost-effectiveness acceptability curve.

The results of the probabilistic sensitivity analysis are shown in Figs 6 and 7, with the total costs and QALYs of each intervention presented. In Fig 6, each point represents one set of results generated from the sampled input parameter values for each treatment option. Fig 7 is the cost-effectiveness acceptability curve (CEAC), showing the probability that each treatment option is cost-effective across a range of willingness-to-pay thresholds.

The scatter plot in Fig 6 shows that, although BMT is less costly than DBS overall, it generates fewer QALYs. Each cloud of points shows the considerable variability in the total QALYs gained for each intervention, reflecting the uncertainty in the long-term disease outcomes and their impact upon health-related quality of life. At a threshold of £20,000 per QALY gained, the probability of DBS being the most cost-effective intervention was 51%, rising to 99% at a £30,000 per QALY threshold (see Fig 7).

## Discussion

This analysis was undertaken to determine the cost-effectiveness of DBS versus alternative treatment options in PD patients from early onset of motor complications on over 15 years, building on existing cost effectiveness evidence of DBS treatment in patients with more advanced disease. This analysis suggests that DBS is a cost-effective intervention with an ICER of £19,887 per QALY gained when compared with BMT and below the UK maximum willingness-to-pay threshold of £30,000 per QALY gained [88].

This is the first analysis using the UPDRS scale to model long-term progression based on a PD population with early onset of motor complications. It supports the majority of the existing economic evidence in more advanced populations that DBS is cost-effective compared with BMT[32–34,89]. The recent analysis of DBS from a Swedish societal perspective showed DBS to be cost-saving compared to BMT [90]. Two published analyses have looked at DBS in advanced PD from a UK health service perspective. Eggington et al found acceptable ICERs in their modelled analyses using clinical data from the Deuschl RCT [29,33]. In contrast, the analysis based on the PD SURG study found less favourable results for DBS [89]. The PDSURG-based analysis used a micro-costing approach to estimate costs related to DBS and BMT, and its consequences over time. However, given that the clinical study was conducted 10–16 years ago, some obsolete practices may be reflected. Outdated practices may affect costs and effects, as patient selection has improved as a result of almost 20 years of experience with DBS, and targeting is more advanced, following the availability of better imaging techniques.

QALY differences between DBS and BMT were very small, non-significant, and possibly indicative of the disease-specific quality of life benefits (PDQ-39), which were smaller than in other RCTs (13% in PD SURG versus 24% and 26% in EARLYSTIM and Deuschl (2006), respectively [29,36,89]. The sensitivity of the PD SURG results to changes in QALYs was demonstrated by one-way sensitivity analyses showing ICERs below willingness-to-pay thresholds when 30% higher QALY benefits were assumed for DBS. These aspects may explain differences in cost-effectiveness results between existing studies and the PD SURG analysis.

Our results are in accord with the comparative previous analyses by Dams et al [32] who find DBS to be highly cost-effective in a sensitivity analysis where they considered an “early” population. However, this previous analysis used an early PD population at the start of the model (in terms of the distribution of patients between Hoehn and Yahr stages), applying all other parameter values as per an advanced PD population.

The modelling approach undertaken here has strengths. We developed an economic model using UPDRS scores to represent disease progression, and created a mapping algorithm to provide a link between these scores and patients’ health-related quality of life. The UPDRS-based model reflects the multi-faceted aspects of Parkinson’s disease, and allowed the use and synthesis of clinical data from multiple studies reporting UPDRS scores as endpoints. We used two-year data from the EARLYSTIM trial to inform the initial model inputs [36], and supplemented this with data identified via a systematic review to project long-term outcomes for each treatment option. Device costs made up the majority of the costs in the DBS treatment group; in the BMT group, drug therapy and management of co-morbidities were the main cost drivers. The ICER remained relatively stable when tested in sensitivity analyses; only in a scenario in which the time horizon was limited to five years was an ICER greater than £30,000 per QALY gained observed. Uncertainty in long-term outcomes for each treatment was fully explored and did not lead to substantial changes in the cost-effectiveness results. Probabilistic sensitivity analysis indicated a high probability of DBS being cost-effective, with much of the variability in QALY gains due to the uncertainty in long-term disease progression rates and the long-term relationship between UPDRS scores and quality of life.

We recognise that our analysis has limitations. The time horizon of 15 years was chosen as the base case in order to capture modelling of long-term health and economic outcomes of a population with early complications at the start of the model and which develop into more advanced disease at later model stages. A lifetime horizon was not considered robust enough given the limitations in the data. The patient data used came from different sources, the longest of which provided 5 year follow-up information after DBS. Thus, heterogeneity of the data sources and the lack of actual patient data beyond 5 years are limitations.

As with all health economic modelling exercises, the analysis required a number of assumptions to be made, in particular with regard to long-term UPDRS outcomes for different treatments. Wherever assumptions were needed, they were conservative, for example assuming identical long-term UPDRS progression rates for DBS and BMT patients, with the exception of UPDRS IV, in which a longer-term benefit for DBS was applied. Further long-term outcomes data are needed to fully validate these assumptions. The use of a new mapping algorithm to derive EQ-5D utilities from UPDRS scores also introduced uncertainty, as its applicability to other datasets has not been evaluated. Nevertheless, the algorithm had high internal validity and builds on existing algorithms by capturing the relationship between a broader range of UPDRS domain scores and quality of life.

Alternative therapies are available for the management of PD with motor complications. In particular, sub-cutaneous apomorphine infusion (CSAI) and continuous duodenal levodopa carbidopa infusion (CDLCI) have been the focus of studies in patients with advanced PD [91–103]. However, evidence regarding the efficacy of their use among patients with early motor symptoms is lacking, and for this reason we excluded these treatment options from this analysis. Existing economic evidence indicates that CDLCI and CSAI are more costly than both DBS and BMT, [104–106] suggesting that DBS would be a more cost-effective alternative to both of these treatments. With availability of more robust comparative clinical data, a formal economic evaluation is needed comparing all treatment options in patients with early complications.

Clinicians are faced with having to make treatment decisions for patients presenting with motor complications on a continuum of care. In particular, strict inclusion criteria apply for patients to undergo DBS, and most available data refer to DBS of the subthalamic nucleus. Many patients in whom this treatment is contraindicated may benefit from CDLCI which is typically a treatment of advanced and very advanced PD.

In conclusion, we modelled cost-effectiveness of DBS in PD patients with early motor complications over a time horizon of 15 years building on evidence from patients with advanced disease. Using a novel mapping algorithm to link disease progression with health-related quality of life, and two-year follow-up data from the EARLYSTIM trial [36], our analysis concludes that the incremental cost-effectiveness ratio of DBS compared to BMT is acceptable based on current thresholds. Our findings are line with the evidence for cost-effectiveness of DBS in advanced PD and provide support from an economical standpoint for the extension of existing policy recommendations to make DBS available to patients with PD with early motor complications.

## Supporting Information

S1 PRISMA Checklist PRISMA 2009 Checklist–Reports of 4 systematic reviews informing DBS CE model. (PDF)

S1 Appendix Appendix–Systematic review and data analyses. (PDF)

S1 Table Parameter input values, sources and distributions for probabilistic sensitivity analysis. (DOCX)

## References

[1] National Institute of Neurological Disorders and Stroke. What is Parkinson’s Disease? [Internet]. 2011. Available: http://www.ninds.nih.gov/disorders/parkinsons_disease/detail_parkinsons_disease.htm.

[2] Ontario Medical Advisory Secretariat. Deep brain stimulation for Parkinson’s disease and other movement disorders [Internet]. 2005. Available: http://www.ncbi.nlm.nih.gov/pmc/articles/PMC3382386/pdf/ohtas-05-56.pdf. PMC3382386 23074471

[3] Pringsheim T, Jette N, Frolkis A, Steeves TDL. The prevalence of Parkinson’s disease: A systematic review and meta-analysis. Movement Disorders [Internet]. 2014;29(13):1583–90. Available: doi: http://doi.wiley.com/10.1002/mds.25945 24976103 10.1002/mds.25945

[4] Horstink M, Tolosa E, Bonuccelli U, Deuschl G, Friedman a, Kanovsky P, et al Review of the therapeutic management of Parkinson’s disease. Report of a joint task force of the European Federation of Neurological Societies and the Movement Disorder Society-European Section. Part I: Early (uncomplicated) Parkinson's disease. European Journal of Neurology. 2006;13(11):1170–85. 17038031 10.1111/j.1468-1331.2006.01547.x

[5] Alves G, Wentzel-Larsen T, Aarsland D, Larsen JP. Progression of motor impairment and disability in Parkinson disease: A population-based study. Neurology. 2005;65(9):1436–41. 16275832 10.1212/01.wnl.0000183359.50822.f2

[6] Leibson CL, Maraganore DM, Bower JH, Ransom JE, O’Brien PC, Rocca WA. Comorbid conditions associated with Parkinson’s disease: A population-based study. Movement Disorders [Internet]. 2006;21(4):446–55. Available: http://doi.wiley.com/10.1002/mds.20685. 16161155 10.1002/mds.20685

[7] Riedel O, Klotsche J, Spottke a, Deuschl G, Förstl H, Henn F, et al Cognitive impairment in 873 patients with idiopathic Parkinson’s disease: Results from the German Study on Epidemiology of Parkinson's Disease with Dementia (GEPAD). Journal of Neurology. 2008;255(2):255–64. 10.1007/s00415-008-0720-2 18204803

[8] Schrag A, Hovris A, Morley D, Quinn N, Jahanshahi M. Young- versus older-onset Parkinson’s disease: Impact of disease and psychosocial consequences. Movement Disorders. 2003;18(11):1250–6. 14639664 10.1002/mds.10527

[9] Winter Y, von Campenhausen S, Popov G, Reese JP, Balzer-Geldsetzer M, Kukshina A, et al Social and clinical determinants of quality of life in Parkinson’s disease in a Russian cohort study. Parkinsonism & related disorders [Internet]. 2010;16(4):243–8. Available: http://www.sciencedirect.com/science/article/pii/S1353802009003022. 20022549 10.1016/j.parkreldis.2009.11.012

[10] Quittenbaum BH, Grahn B. Quality of life and pain in Parkinson’s disease: a controlled cross-sectional study. Parkinsonism Relat Disord [Internet]. 2004;10(3):129–36. Available: http://www.ncbi.nlm.nih.gov/pubmed/15036166. 15036166 10.1016/j.parkreldis.2003.12.001

[11] Riazi A, Hobart JC, Lamping DL, Fitzpatrick R, Freeman J a, Jenkinson C, et al Using the SF-36 measure to compare the health impact of multiple sclerosis and Parkinson’s disease with normal population health profiles. Journal of neurology, neurosurgery, and psychiatry. 2003;74(6):710–4. 12754336 10.1136/jnnp.74.6.710 PMC1738466

[12] Schrag A, Jahanshahi M, Quinn N. What contributes to quality of life in patients with Parkinson’s disease? Journal of neurology, neurosurgery, and psychiatry. 2000;69(3):308–12. 10945804 10.1136/jnnp.69.3.308 PMC1737100

[13] Rahman S, Griffin HJ, Quinn NP, Jahanshahi M. Quality of life in Parkinson’s disease: The relative importance of the symptoms. Movement Disorders [Internet]. 2008;23(10):1428–34. Available: doi: http://doi.wiley.com/10.1002/mds.21667 18543333 10.1002/mds.21667

[14] Visser M, van Rooden SM, Verbaan D, Marinus J, Stiggelbout AM, van Hilten JJ. A comprehensive model of health-related quality of life in Parkinson’s disease. Journal of Neurology [Internet]. 2008;255(10):1580–7. Available: http://link.springer.com/10.1007/s00415-008-0994-4. 10.1007/s00415-008-0994-4 18821041

[15] Marras C, McDermott MP, Rochon PA, Tanner CM, Naglie G, Lang AE. Predictors of deterioration in health-related quality of life in Parkinson’s disease: Results from the DATATOP trial. Movement Disorders. 2008;23(5):653–9. 18076084 10.1002/mds.21853

[16] Woodford H, Walker R. Emergency hospital admissions in idiopathic’s Parkinson's disease. Movement Disorders. 2005;20(9):1104–8. 15884038 10.1002/mds.20485

[17] Spottke AE, Reuter M, Machat O, Bornschein B, Von Campenhausen S, Berger K, et al Cost of illness and its predictors for Parkinson’s disease in Germany. PharmacoEconomics. 2005;23(8):817–36. 16097843 10.2165/00019053-200523080-00007

[18] Findley L, Aujla M, Bain PG, Baker M, Beech C, Bowman C, et al Direct economic impact of Parkinson’s disease: A research survey in the United Kingdom. Movement Disorders. 2003;18(10):1139–45. 14534917 10.1002/mds.10507

[19] Dengler I, Leukel N, Meuser T, Jost WH. Prospektive Erfassung der direkten und indirekten Kosten des idiopathischen Parkinson-Syndroms. Der Nervenarzt [Internet]. 2006;77(10):1204–9. Available: http://link.springer.com/10.1007/s00115-006-2150-7. 17004081 10.1007/s00115-006-2150-7

[20] Vossius C, Gjerstad M, Baas H, Larsen JP. Drug costs for patients with Parkinson’s disease in two different European countries. Acta neurologica Scandinavica. 2006;113(4):228–32. 16542161 10.1111/j.1600-0404.2005.00574.x

[21] Péchevis M, Clarke CE, Vieregge P, Khoshnood B, Deschaseaux-Voinet C, Berdeaux G, et al Effects of dyskinesias in Parkinson’s disease on quality of life and health-related costs: A prospective European study. European Journal of Neurology. 2005;12(12):956–63. 16324089 10.1111/j.1468-1331.2005.01096.x

[22] Dodel RC, Singer M, Köhne-Volland R, Szucs T, Rathay B, Scholz E, et al The economic impact of Parkinson’s disease. An estimation based on a 3-month prospective analysis. PharmacoEconomics. 1998;14(3):299–312. 10186468 10.2165/00019053-199814030-00006

[23] McCrone P, Allcock LM, Burn DJ. Predicting the cost of Parkinson’s disease. Movement Disorders [Internet]. 2007;22(6):804–12. Available: http://doi.wiley.com/10.1002/mds.21360. 17290462 10.1002/mds.21360

[24] Keränen T, Kaakkola S, Sotaniemi K, Laulumaa V, Haapaniemi T, Jolma T, et al Economic burden and quality of life impairment increase with severity of PD. Parkinsonism and Related Disorders. 2003;9(3):163–8. 12573872 10.1016/s1353-8020(02)00097-4

[25] Maurel F, Lilliu H, Le Pen C. [Social and economic cost of L-Dopa-induced dyskinesias in patients with Parkinson’s disease]. Revue neurologique [Internet]. 2001 5 [cited 2015 Nov 17];157(5):507–14. Available: http://www.ncbi.nlm.nih.gov/pubmed/11438770. 11438770

[26] Schrag A, Banks P. Time of loss of employment in Parkinson’s disease. Movement Disorders. 2006;21(11):1839–43. 16941456 10.1002/mds.21030

[27] Hagell P, Nordling S, Reimer J, Grabowski M, Persson U. Resource use and costs in a Swedish cohort of patients with Parkinson’s disease. Movement disorders: official journal of the Movement Disorder Society [Internet]. 2002;17(6):1213–20. Available: http://www.ncbi.nlm.nih.gov/pubmed/12465059. 12465059 10.1002/mds.10262

[28] National Institute for Health and Care Excellence. Parkinson’s Disease in Over 20s: Diagnosis and Management [Internet]. 2006. Available: http://www.nice.org.uk/guidance/cg35/resources/parkinsons-disease-in-over-20s-diagnosis-and-management-975388237765.

[29] Deuschl G, Schade-Brittinger C, Krack P, Volkmann J, Schäfer H, Bötzel K, et al A Randomized Trial of Deep-Brain Stimulation for Parkinson’s Disease. N Engl J Med. 2006;9355:896–908. 10.1056/NEJMoa060281 16943402

[30] Weaver F, Follett K, Stern M, Hur K. Bilateral deep brain stimulation vs best medical therapy for patients with advanced Parkinson disease. the American Medical [Internet]. 2009; Available: http://jama.ama-assn.org/content/301/1/63.short.

[31] Williams A, Gill S, Varma T, Jenkinson C, Quinn N, Mitchell R, et al Deep brain stimulation plus best medical therapy versus best medical therapy alone for advanced Parkinson’s disease (PD SURG trial): a randomised, open-label trial. The Lancet Neurology. 2010;9(6):581–91. 10.1016/S1474-4422(10)70093-4 20434403 PMC2874872

[32] Dams J, Siebert U, Bornschein B, Volkmann J, Deuschl G, Oertel WH, et al Cost-effectiveness of deep brain stimulation in patients with Parkinson’s disease. Movement Disorders. 2013;28(6):763–71. 10.1002/mds.25407 23576266

[33] Eggington S, Valldeoriola F, Chaudhuri KR, Ashkan K, Annoni E, Deuschl G. The cost-effectiveness of deep brain stimulation in combination with best medical therapy, versus best medical therapy alone, in advanced Parkinson’s disease. Journal of Neurology. 2014;261(1):106–16. 10.1007/s00415-013-7148-z 24158271 PMC3895185

[34] Valldeoriola F, Morsi O, Tolosa E, Rumià J, Martí MJ, Martínez-Martín P. Prospective comparative study on cost-effectiveness of subthalamic stimulation and best medical treatment in advanced Parkinson’s disease. Movement Disorders. 2007;22(15):2183–91. 17724747 10.1002/mds.21652

[35] National Collaborating Centre for Chronic Conditions. Parkinson’s Disease: National clinical guideline for diagnosis and management in primary and secondary care. [Internet]. 2006. Available: https://www.nice.org.uk/guidance/cg35/evidence/full-guideline-194930029.

[36] Schuepbach WMM, Rau J, Knudsen K, Volkmann J, Krack P, Timmermann L, et al Neurostimulation for Parkinson’s Disease with Early Motor Complications. New England Journal of Medicine [Internet]. 2013;368(7):610–22. Available: doi: http://www.nejm.org/doi/abs/10.1056/NEJMoa1205158 23406026 10.1056/NEJMoa1205158

[37] Schuepbach WMM, Maltête D, Houeto JL, Du Montcel ST, Mallet L, Welter ML, et al Neurosurgery at an earlier stage of Parkinson disease: A randomized, controlled trial. Neurology. 2007;68(4):267–71. 17151341 10.1212/01.wnl.0000250253.03919.fb

[38] Caro JJ, Briggs AH, Siebert U, Kuntz KM. Modeling good research practices—overview: a report of the ISPOR-SMDM Modeling Good Research Practices Task Force—1. Value in health. 2012;15(6):796–803. 10.1016/j.jval.2012.06.012 22999128

[39] Siebert U, Alagoz O, Bayoumi AM, Jahn B. State-Transition Modeling: A Report of the ISPOR-SMDM Modeling Good Research Practices Task Force-3. Value in Health. 2012;15:812–20. 10.1016/j.jval.2012.06.014 22999130

[40] National Institute for Health and Care Excellence. Guide to the methods of technology appraisal [Internet]. Available: http://publications.nice.org.uk/pmg9. 27905712

[41] Brooks DJ, Leinonen M, Kuoppamäki M, Nissinen H. Five-year efficacy and safety of levodopa/DDCI and entacapone in patients with Parkinson’s disease. Journal of neural transmission (Vienna, Austria: 1996) [Internet]. 2008 Jun [cited 2015 Dec 1];115(6):843–9. Available: http://www.ncbi.nlm.nih.gov/pubmed/18259682. 10.1007/s00702-008-0025-8 18259682

[42] Jankovic J, Kapadia AS. Functional decline in Parkinson disease. Archives of neurology. 2001;58(10):1611–5. 11594919 10.1001/archneur.58.10.1611

[43] LeWitt PA, Boroojerdi B, Surmann E, Poewe W. Rotigotine transdermal system for long-term treatment of patients with advanced Parkinson’s disease: results of two open-label extension studies, CLEOPATRA-PD and PREFER. Journal of neural transmission (Vienna, Austria: 1996) [Internet]. 2013 7 [cited 2015 Dec 1];120(7):1069–81. Available: http://www.pubmedcentral.nih.gov/articlerender.fcgi?artid=3687107&tool=pmcentrez&rendertype=abstract. 10.1007/s00702-012-0925-5 PMC3687107 23208198

[44] Reinoso G, Allen JC, Au W-L, Seah S-H, Tay K-Y, Tan LCS. Clinical evolution of Parkinson’s disease and prognostic factors affecting motor progression: 9-year follow-up study. European journal of neurology: the official journal of the European Federation of Neurological Societies [Internet]. 2015 3 [cited 2015 Nov 6];22(3):457–63. Available: http://www.ncbi.nlm.nih.gov/pubmed/24888502. 10.1111/ene.12476 24888502

[45] Krack P, Batir A, Van Blercom N, Chabardes S, Fraix V, Ardouin C, et al Five-year follow-up of bilateral stimulation of the subthalamic nucleus in advanced Parkinson’s disease. The New England journal of medicine. 2003;349(20):1925–34. 14614167 10.1056/NEJMoa035275

[46] Schuepbach WMM, Chastan N, Welter ML, Houeto JL, Mesnage V, Bonnet AM, et al Stimulation of the subthalamic nucleus in Parkinson’s disease: a 5 year follow up. Journal of neurology, neurosurgery, and psychiatry. 2005;76(12):1640–4. 16291886 10.1136/jnnp.2005.063206 PMC1739461

[47] Wider C, Pollo C, Bloch J, Burkhard PR, Vingerhoets FJG. Long-term outcome of 50 consecutive Parkinson’s disease patients treated with subthalamic deep brain stimulation. Parkinsonism & related disorders. 2008;14(2):114–9. 17822940 10.1016/j.parkreldis.2007.06.012

[48] Gervais-Bernard H, Xie-Brustolin J, Mertens P, Polo G, Klinger H, Adamec D, et al Bilateral subthalamic nucleus stimulation in advanced Parkinson’s disease: Five year follow-up. Journal of Neurology. 2009;256(2):225–33. 10.1007/s00415-009-0076-2 19242649

[49] Moro E, Lozano AM, Pollak P, Agid Y, Rehncrona S, Volkmann J, et al Long-term results of a multicenter study on subthalamic and pallidal stimulation in Parkinson’s disease. Movement Disorders. 2010;25(5):578–86. 10.1002/mds.22735 20213817

[50] Fasano A, Romito LM, Daniele A, Piano C, Zinno M, Bentivoglio AR, et al Motor and cognitive outcome in patients with Parkinson’s disease 8 years after subthalamic implants. Brain. 2010;133(9):2664–76. 10.1093/brain/awq221 20802207

[51] Zibetti M, Merola A, Rizzi L, Ricchi V, Angrisano S, Azzaro C, et al Beyond nine years of continuous subthalamic nucleus deep brain stimulation in Parkinson’s disease. Movement Disorders. 2011;26(13):2327–34. 10.1002/mds.23903 22012750

[52] Merola A, Espay AJ, Romagnolo A, Bernardini A, Rizzi L, Rosso M, et al Advanced therapies in Parkinson’s disease: Long-term retrospective study. Parkinsonism & Related Disorders [Internet]. Elsevier Ltd; 2016; Available: http://linkinghub.elsevier.com/retrieve/pii/S1353802016301675. 10.1016/j.parkreldis.2016.05.015 27215392

[53] Office for National Statistics. National Life Tables, United Kingdom [Internet]. Available: http://www.ons.gov.uk/ons/rel/lifetables/national-life-tables/2012-2014/ref-uk.xls.

[54] Peretz C, Chillag-Talmor O, Linn S, Gurevich T, El-Ad B, Silverman B, et al Parkinson’s disease patients first treated at age 75 years or older: A comparative study. Parkinsonism & related disorders [Internet]. 2014;20(1):69–74. Available: http://www.ncbi.nlm.nih.gov/pubmed/24183677. 24183677 10.1016/j.parkreldis.2013.09.020

[55] Williams-Gray CH, Mason SL, Evans JR, Foltynie T, Brayne C, Robbins TW, et al The CamPaIGN study of Parkinson’s disease: 10-year outlook in an incident population-based cohort. Journal of Neurology, Neurosurgery & Psychiatry [Internet]. 2013;84(11):1258–64. Available: http://jnnp.bmj.com/cgi/doi/10.1136/jnnp-2013-305277. 10.1136/jnnp-2013-305277 23781007

[56] Duarte J, García Olmos LM, Mendoza A, Clavería LE. The natural history of Parkinson’s disease in the province of Segovia: mortality in a longitudinal study (20-year follow-up). Acta neurologica Scandinavica [Internet]. 2013;127(5):295–300. Available: http://www.ncbi.nlm.nih.gov/pubmed/22957805. 10.1111/ane.12003 22957805

[57] Macleod AD, Taylor KSM, Counsell CE. Mortality in Parkinson’s disease: a systematic review and meta-analysis. Movement disorders: official journal of the Movement Disorder Society [Internet]. 2014;29(13):1615–22. Available: http://www.ncbi.nlm.nih.gov/pubmed/24821648. 24821648 10.1002/mds.25898

[58] Marras C, McDermott MP, Rochon PA, Tanner CM, Naglie G, Rudolph A, et al Survival in Parkinson disease: thirteen-year follow-up of the DATATOP cohort. Neurology. 2005;64(1):87–93. 15642909 10.1212/01.WNL.0000148603.44618.19

[59] Forsaa E, Larsen J, Alves G. What predicts mortality in Parkinson disease? Neurology. 2010;75:1270–6. 10.1212/WNL.0b013e3181f61311 20921512

[60] Skorvanek M, Rosenberger J, Rajnicova I, van Dijk J, Groothoff J, Gdovinova Z. Fatigue is not an independent predictor of mortality in Parkinson’s disease. Parkinsonism & Related Disorders [Internet]. 2012;18(Suoplement 1):S21–2. Available: http://www.sciencedirect.com/science/article/pii/S1353802011701626.22166437

[61] Ashburn A, Stack E, Pickering RM, Ward CD. A community-dwelling sample of people with Parkinson ‘ s disease: characteristi… 2001;277–81. 10.1093/ageing/30.1.47 11322672

[62] Bloem BR, Grimbergen YAM, Cramer M, Willemsen M, Zwinderman AH. Prospective assessment of falls in Parkinson’s disease. Journal of Neurology. 2001;248(11):950–8. 11757958 10.1007/s004150170047

[63] Cheng K-Y, Lin W-C, Chang W-N, Lin T-K, Tsai N-W, Huang C-C, et al Factors associated with fall-related fractures in Parkinson’s disease. Parkinsonism & related disorders [Internet]. 2014 1 [cited 2015 Dec 2];20(1):88–92. Available: http://www.ncbi.nlm.nih.gov/pubmed/24134900. 24134900 10.1016/j.parkreldis.2013.09.024

[64] Contreras A, Grandas F. Risk of Falls in Parkinson’s Disease: A Cross-Sectional Study of 160 Patients. Parkinson’s Disease [Internet]. 2012;2012:1–10. Available: http://www.hindawi.com/journals/pd/2012/362572/. 10.1155/2012/362572 PMC3265111 22292126

[65] Gazibara T, Pekmezovic T, Kisic Tepavcevic D, Tomic A, Stankovic I, Kostic VS, et al Fall frequency and risk factors in patients with Parkinson’s disease in Belgrade, Serbia: a cross-sectional study. Geriatrics & gerontology international [Internet]. 2015 4 [cited 2015 Dec 2];15(4):472–80. Available: http://www.ncbi.nlm.nih.gov/pubmed/24774885. 24774885 10.1111/ggi.12300

[66] Kataoka H, Tanaka N, Eng M, Saeki K, Kiriyama T, Eura N, et al Risk of falling in Parkinson’s disease at the Hoehn-Yahr stage III. European neurology. 2011;66(5):298–304. 10.1159/000331635 22057308

[67] Kerr GK, Worringham CJ, Cole MH, Lacherez PF, Wood JM, Silburn PA. Predictors of future falls in Parkinson disease. Neurology [Internet]. 2010 7 13 [cited 2015 Dec 2];75(2):116–24. Available: http://www.ncbi.nlm.nih.gov/pubmed/20574039. 10.1212/WNL.0b013e3181e7b688 20574039

[68] Latt MD, Lord SR, Morris JGL, Fung VSC. Clinical and physiological assessments for elucidating falls risk in Parkinson’s disease. Movement disorders: official journal of the Movement Disorder Society [Internet]. 2009;24(9):1280–9. Available: http://www.ncbi.nlm.nih.gov/pubmed/19425059. 19425059 10.1002/mds.22561

[69] Lindholm B, Hagell P, Hansson O, Nilsson MH. Prediction of falls and/or near falls in people with mild Parkinson’s disease. PloS one [Internet]. 2015 1 [cited 2015 Dec 2];10(1):e0117018 Available: http://www.pubmedcentral.nih.gov/articlerender.fcgi?artid=4311993&tool=pmcentrez&rendertype=abstract. 10.1371/journal.pone.0117018 25635687 PMC4311993

[70] Matinolli M, Korpelainen JT, Sotaniemi KA, Myllylä V V, Korpelainen R. Recurrent falls and mortality in Parkinson’s disease: a prospective two-year follow-up study. Acta neurologica Scandinavica [Internet]. 2011 3 [cited 2015 Dec 2];123(3):193–200. Available: http://www.ncbi.nlm.nih.gov/pubmed/20545629. 10.1111/j.1600-0404.2010.01386.x 20545629

[71] Parashos SA, Wielinski CL, Giladi N, Gurevich T. Falls in Parkinson disease: analysis of a large cross-sectional cohort. Journal of Parkinson’s disease [Internet]. 2013 1 [cited 2015 Dec 2];3(4):515–22. Available: http://www.ncbi.nlm.nih.gov/pubmed/24113557. 10.3233/JPD-130249 24113557

[72] Rudzińska M, Bukowczan S, Stożek J, Zajdel K, Mirek E, Chwała W, et al The incidence and risk factors of falls in Parkinson disease: prospective study. Neurologia i Neurochirurgia Polska [Internet]. 2013;47(5):431–7. Available: http://www.sciencedirect.com/science/article/pii/S0028384314604357. 24166564 10.5114/ninp.2013.38223

[73] Voss TS, Elm JJ, Wielinski CL, Aminoff MJ, Bandyopadhyay D, Chou KL, et al Fall frequency and risk assessment in early Parkinson’s disease. Parkinsonism & related disorders [Internet]. 2012 8 [cited 2015 Dec 2];18(7):837–41. Available: http://www.pubmedcentral.nih.gov/articlerender.fcgi?artid=3424355&tool=pmcentrez&rendertype=abstract. 22542094 10.1016/j.parkreldis.2012.04.004 PMC3424355

[74] Wielinski CL, Erickson-Davis C, Wichmann R, Walde-Douglas M, Parashos SA. Falls and injuries resulting from falls among patients with Parkinson’s disease and other parkinsonian syndromes. Movement disorders: official journal of the Movement Disorder Society [Internet]. 2005 4 [cited 2015 Dec 2];20(4):410–5. Available: http://www.ncbi.nlm.nih.gov/pubmed/15580552. 15580552 10.1002/mds.20347

[75] Wood BH, Bilclough JA, Bowron A, Walker RW. Incidence and prediction of falls in Parkinson’s disease: a prospective multidisciplinary study. Journal of neurology, neurosurgery, and psychiatry. 2002;72(6):721–5. 12023412 10.1136/jnnp.72.6.721 PMC1737913

[76] Kent S, Gray A, Schlackow I, Jenkinson C, McIntosh E. Mapping from the Parkinson’s Disease Questionnaire PDQ-39 to the Generic EuroQol EQ-5D-3L: The Value of Mixture Models. Medical Decision Making [Internet]. 2015;1–10. Available: http://mdm.sagepub.com/cgi/doi/10.1177/0272989X15584921. 10.1177/0272989X15584921 25926283

[77] Kind P, Hardman G, Macran S. UK population norms for EQ-5D. Working Papers [Internet]. Centre for Health Economics, University of York; 1999 [cited 2016 Jan 8]; Available: http://www.mendeley.com/catalog/uk-population-norms-eq5d/.

[78] Martínez-Martín P, Rodríguez-Blázquez C, Forjaz MJ, Álvarez-Sánchez M, Arakaki T, Bergareche-Yarza a., et al Relationship between the MDS-UPDRS domains and the health-related quality of life of Parkinson’s disease patients. European Journal of Neurology. 2014;21(3):519–24. 10.1111/ene.12349 24447695

[79] Kadastik-Eerme L, Rosenthal M, Paju T, Muldmaa M, Taba P. Health-related quality of life in Parkinson’s disease: a cross-sectional study focusing on non-motor symptoms. Health and Quality of Life Outcomes [Internet]. Health and Quality of Life Outcomes; 2015;13(1):83 Available: http://hqlo.biomedcentral.com/articles/10.1186/s12955-015-0281-x. 26088201 10.1186/s12955-015-0281-x PMC4474578

[80] Dams J, Klotsche J, Bornschein B, Reese JP, Balzer-Geldsetzer M, Winter Y, et al Mapping the EQ-5D index by UPDRS and PDQ-8 in patients with Parkinson’s disease. Health and quality of life outcomes [Internet]. 2013 1 [cited 2015 Dec 3];11:35 Available: http://www.pubmedcentral.nih.gov/articlerender.fcgi?artid=3662160&tool=pmcentrez&rendertype=abstract. 10.1186/1477-7525-11-35 23497005 PMC3662160

[81] NHS England. National Tariff Payment System 2015–16 [Internet]. 2015. Available: https://www.gov.uk/government/uploads/system/uploads/attachment_data/file/379578/Annex_5a.xlsx.

[82] British Medical Association & Royal Pharmaceutical Society. British National Formulary, 70 Pharmaceutical Press; 2015.

[83] Curtis L. Unit costs of health and social care, 2014. 2014.

[84] Medtronic Neuromodulation Price List. 2010.

[85] Medtronic internal data on file: MRCS NDHF1481-142128.

[86] Murphy A. Economic evaluations for health technologies with an evolving evidence base: a case study of transcatheter aortic valve implantation [Internet]. 2013. Available: http://theses.gla.ac.uk/4061/.

[87] Weir S, Samnaliev M, Kuo T-C, Choitir CN, Tierney T, Taylor R, et al. Analyses of healthcare resource utilization in Parkinson’s disease in the United Kingdom using CPRD. Internal Report. 2016.

[88] McCabe C, Claxton K, Culyer AJ. The NICE cost-effectiveness threshold: What it is and what that means. PharmacoEconomics. 2008;26(9):733–44. 18767894 10.2165/00019053-200826090-00004

[89] McIntosh E, Gray A, Daniels J, Gill S, Ives N, Jenkinson C, et al Cost-utility analysis of deep brain stimulation surgery plus best medical therapy versus best medical therapy in patients with Parkinson’s: Economic evaluation alongside the PD SURG trial. Movement disorders: official journal of the Movement Disorder Society [Internet]. 2016 2 5 [cited 2016 Feb 7]; Available: http://www.ncbi.nlm.nih.gov/pubmed/26846185. 10.1002/mds.26423 26846185

[90] SocialStyrelsen. Nationella riktlinjer för vård vid multipel skleros (MS) och Parkinsons sjukdom–Stöd för styrning och ledning–Remissversion 2016, Tillstånds- och åtgärdslista [Internet]. 2016. Available: http://www.socialstyrelsen.se/publikationer2016/2016-2-1.

[91] Drapier S, Gillioz A-S, Leray E, Péron J, Rouaud T, Marchand A, et al Apomorphine infusion in advanced Parkinson’s patients with subthalamic stimulation contraindications. Parkinsonism & Related Disorders [Internet]. Elsevier Ltd; 2012;18(1):40–4. Available: http://linkinghub.elsevier.com/retrieve/pii/S1353802011002586. 21890396 10.1016/j.parkreldis.2011.08.010

[92] Antonini A, Mancini F, Canesi M, Zangaglia R, Isaias IU, Manfredi L, et al Duodenal levodopa infusion improves quality of life in advanced Parkinson’s disease. Neurodegenerative Diseases. 2008;5(3–4):244–6. 10.1159/000113714 18322402

[93] Antonini a, Odin P, Opiano L, Tomantschger V, Pacchetti C, Pickut B, et al Effect and safety of duodenal levodopa infusion in advanced Parkinson’s disease: a retrospective multicenter outcome assessment in patient routine care. Journal of neural transmission (Vienna, Austria: 1996) [Internet]. 2013;120(11):1553–8. Available: http://www.ncbi.nlm.nih.gov/pubmed/23595879. 10.1007/s00702-013-1026-9 23595879

[94] Antonini A, Yegin A, Preda C, Bergmann L, Poewe W. Global long-term study on motor and non-motor symptoms and safety of levodopa-carbidopa intestinal gel in routine care of advanced Parkinson’s disease patients; 12-month interim outcomes. Parkinsonism & related disorders [Internet]. 2015 3 [cited 2015 Nov 1];21(3):231–5. Available: http://www.ncbi.nlm.nih.gov/pubmed/25585993. 25585993 10.1016/j.parkreldis.2014.12.012

[95] Fernandez HH, Standaert DG, Hauser RA, Lang AE, Fung VSC, Klostermann F, et al Levodopa-carbidopa intestinal gel in advanced Parkinson’s disease: Final 12-month, open-label results. Movement Disorders [Internet]. 2015;30(4):500–9. Available: doi: http://doi.wiley.com/10.1002/mds.26123 25545465 10.1002/mds.26123 PMC4674978

[96] Martinez-Martin P, Reddy P, Katzenschlager R, Antonini A, Todorova A, Odin P, et al EuroInf: A multicenter comparative observational study of apomorphine and levodopa infusion in Parkinson’s disease. Movement disorders: official journal of the Movement Disorder Society [Internet]. 2014;00(00):1–7. Available: http://www.ncbi.nlm.nih.gov/pubmed/25382161. 10.1002/mds.26067 25382161

[97] Pålhagen SE, Dizdar N, Hauge T, Holmberg B, Jansson R, Linder J, et al Interim analysis of long-term intraduodenal levodopa infusion in advanced Parkinson disease. Acta neurologica Scandinavica [Internet]. 2012;126(6):e29–33. Available: http://www.ncbi.nlm.nih.gov/pubmed/22690905. 10.1111/j.1600-0404.2012.01689.x 22690905

[98] Sensi M, Preda F, Trevisani L, Contini E, Gragnaniello D, Capone J, et al Emerging issues on selection criteria of levodopa carbidopa infusion therapy: Considerations on outcome of 28 consecutive patients. Journal of Neural Transmission [Internet]. 2014;121(6):633–42. Available: http://www.ncbi.nlm.nih.gov/pubmed/24398781\nhttp://www.embase.com/search/results?subaction=viewrecord&from=export&id=L52947243\n 10.1007/s00702-013-1153-3\nhttp://sfx.bibl.ulaval.ca:9003/sfx_local?sid=EMBASE&issn=14351463&id=doi:10.1007/. 10.1007/s00702-013-1153-3 24398781

[99] Olanow CW, Kieburtz K, Odin P, Espay AJ, Standaert DG, Fernandez HH, et al Continuous intrajejunal infusion of levodopa-carbidopa intestinal gel for patients with advanced Parkinson’s disease: a randomised, controlled, double-blind, double-dummy study. The Lancet Neurology [Internet]. 2014 2 [cited 2016 Jan 11];13(2):141–9. Available: http://www.ncbi.nlm.nih.gov/pubmed/24361112. 10.1016/S1474-4422(13)70293-X 24361112 PMC4643396

[100] Slevin JT, Fernandez HH, Zadikoff C, Hall C, Eaton S, Dubow J, et al Long-term safety and maintenance of efficacy of levodopa-carbidopa intestinal gel: an open-label extension of the double-blind pivotal study in advanced Parkinson’s disease patients. Journal of Parkinson’s disease [Internet]. 2015 1 [cited 2015 Nov 13];5(1):165–74. Available: http://www.ncbi.nlm.nih.gov/pubmed/25588353. 10.3233/JPD-140456 25588353

[101] Cáceres-Redondo MT, Carrillo F, Lama MJ, Huertas-Fernández I, Vargas-González L, Carballo M, et al Long-term levodopa/carbidopa intestinal gel in advanced Parkinson’s disease. Journal of Neurology [Internet]. 2014;261(3):561–9. Available: http://link.springer.com/10.1007/s00415-013-7235-1. 10.1007/s00415-013-7235-1 24477490

[102] Honig H, Antonini A, Martinez-Martin P, Forgacs I, Faye GC, Fox T, et al Intrajejunal levodopa infusion in Parkinson’s disease: a pilot multicenter study of effects on nonmotor symptoms and quality of life. Movement disorders: official journal of the Movement Disorder Society. 2009;24(10):1468–74. 19425079 10.1002/mds.22596

[103] Karlsborg M, Korbo L, Regeur L, Glad a. Duodopa pump treatment in patients with advanced Parkinson’s disease. Dan Med Bull [Internet]. 2010;57(6):A4155 Available: http://www.ncbi.nlm.nih.gov/pubmed/20515603. 20515603

[104] Valldeoriola F, Puig-Junoy J, Puig-Peiró R. Cost analysis of the treatments for patients with advanced Parkinson’s disease: SCOPE study. Journal of medical economics [Internet]. 2013;16(2):191–201. Available: http://www.ncbi.nlm.nih.gov/pubmed/23035627. 10.3111/13696998.2012.737392 23035627

[105] Walleser Autiero S, Eggington S, Valyi A. Cost comparison of deep brain stimulation (DBS) and continued subcutaneous apomorphine infusion (CSAI) in patients with advanced Parkinson’s disease. In: International Society for Pharmacoeconomics and Outcomes Research. 2014. 10.1016/j.jval.2014.08.881 27200927

[106] Eggington S, Valldeoriola F, Walleser S. Cost comparison of deep brain stimulation and levodopa-carbidopa intestinal gel in patients with advanced Parkinson’s disease. In: 33rd Health Economics Congress of the Spanish Health Economics Association. 2013.

