# Supplementary material for: Deep Brain Stimulation for Parkinson’s Disease with Early Motor Complications: A UK Cost-Effectiveness Analysis
Source: PLoS One. 2016 Jul 21;11(7):e0159340. doi: 10.1371/journal.pone.0159340 (PMC4956248; doi:10.1371/journal.pone.0159340)
Supplement: S1 PRISMA Checklist — (PDF) [file pone.0159340.s001.pdf]

COST-EFFECTIVENESS ANALYSIS

DEEP BRAIN STIMULATION IN PARKINSON'S DISEASE

WITH EARLY MOTOR COMPLICATIONS

APPENDIX

SYSTEMATIC REVIEW & DATA ANALYSES

The analysis was commissioned and financed by Medtronic International Trading Sàrl

Version 2.0

April 2016

TABLE OF CONTENTS

APPENDIX A. SYSTEMATIC REVIEWS 7

A.1. CSAI / CDLCI data 7

A.2. Long-term DBS effectiveness 28

A.3. Long-term progression in BMT studies 48

A.4. Mortality 55

APPENDIX B. DATA ANALYSES FOR MODEL INPUT 61

B.1. CSAI and CDLCI short-term effectiveness (first 2 years in the model) 61

B.2. Long-term effectiveness/progression 71

B.3. Adverse events 76

B.4. Withdrawal 78

B.5. CSAI dose 81

B.6. Falls 82

B.7. Mortality 87

REFERENCES 91

INDEX OF FIGURES

Figure 1. Overview of studies selection: PRISMA flowchart – CSAI/CDLCI data 12
Figure 2. Overview of studies selection: PRISMA flowchart – long-term DBS effectiveness 41
Figure 3. Overview of studies selection: PRISMA flowchart – long-term progression in BMT studies 51
Figure 4. Overview of studies selection: PRISMA flowchart – mortality data 58

INDEX OF TABLES

Table 1. Search strategy in Medline database – CSAI / CDLCI effectiveness 8
Table 2. Search strategy in Embase database – CSAI / CDLCI effectiveness 9
Table 3. Search strategy in Cochrane Library database – CSAI / CDLCI effectiveness 10
Table 4. Selection criteria for clinical studies on CSAI / CDLCI effectiveness in Parkinson Disease 11
Table 5. Summary of systematic search for CSAI / CDLCI effectiveness 12
Table 6. Studies initially included in the analysis and final selection – CSAI 14
Table 7. Studies initially included in the analysis and final selection – CDLCI 15
Table 8 CSAI studies included in final analysis – short-term effectiveness 17
Table 9 Study characteristic – CSAI short-term effectiveness 17
Table 10. CSAI effectiveness – UPDRS – data extraction 18
Table 11 CSAI studies included in final analysis – other parameters 19
Table 12. CSAI withdrawal – data extraction 20
Table 13. CSAI related adverse events – skin nodules / skin reaction – data extraction 20
Table 14. Daily doses of CSAI – data extraction 21
Table 15 CDLCI studies included in final analysis – short-term effectiveness 22
Table 16 Study characteristic – CDLCI short-term effectiveness 22
Table 17. CDLCI effectiveness – UPDRS – data extraction 24
Table 18 CDLCI studies included in final analysis – other parameters 26
Table 19. CDLCI withdrawal – data extraction 26
Table 20. CDLCI related adverse events – peritonitis – data extraction 27
Table 21. Search strategy in Medline database – DBS effectiveness 28
Table 22. Search strategy in Embase database – DBS effectiveness 29
Table 23. Search strategy in Cochrane Library database – DBS effectiveness 29
Table 24. Selection criteria for clinical studies on DBS effectiveness in Parkinson Disease 30
Table 25. Summary of systematic search for DBS effectiveness studies 31
Table 26. Studies initially included in the analysis and final selection – long-term DBS effectiveness 31
Table 27 Long-term DBS effectiveness studies included in the analysis 42
Table 28 Study characteristic – DBS long-term effectiveness 42
Table 29. DBS long-term effectiveness - UPDRS I – data extraction 45
Table 30. DBS long-term effectiveness - UPDRS II (on med / on stim) – data extraction 45
Table 31. DBS long-term effectiveness - UPDRS III (on med / on stim) – data extraction 46
Table 32. DBS long-term effectiveness - UPDRS IV – data extraction 47
Table 33. Search strategy in Medline database – natural disease progression 48
Table 34. Selection criteria for studies on Parkinson's Disease progression in BMT treated patients 49
Table 35. Summary of systematic search for PD progression in BMT treated patients 50
Table 36 Summary of systematic search for PD progression in BMT treated patients – Additional search 50
Table 37 Parkinson's Disease progression in BMT treated patients in final analysis 52
Table 38. Studies on BMT progression not included in final calculation 52
Table 39 Study characteristic – BMT long-term progression 54
Table 40. Long-term UPDRS progression – data extraction 55
Table 41. Search strategy for mortality data in Medline database – systematic reviews 55
Table 42. Search strategy for mortality data in Medline database – primary studies 56
Table 43. Selection criteria for studies on mortality data 57
Table 44. Summary of systematic search for mortality data – additional search 57
Table 45. Summary of systematic search for mortality data – studies included in MacLeod 2014 review 57

Table 46 Studies reporting association between UPDRS and mortality included in final analysis 59
Table 47. Studies on mortality not included in final calculation (included after initial selection) 59
Table 48 Association between UPDRS III and mortality – data extraction 60
Table 49. CSAI effectiveness– UPDRS I – data included in the analysis...61
Table 50. CSAI effectiveness – UPDRS I – CE model assumptions 61
Table 51. CSAI effectiveness– UPDRS II – data included in the analysis...62
Table 52. CSAI effectiveness – UPDRS II – CE model assumptions 62
Table 53. CSAI effectiveness– UPDRS III – data included in the analysis...62
Table 54. CSAI effectiveness – UPDRS III – CE model assumptions 63
Table 55. CSAI effectiveness– UPDRS IV – data included in the analysis 63
Table 56. CSAI effectiveness – UPDRS IV – relative effectiveness (1 year vs 2 years) data 64
Table 57. CSAI effectiveness – UPDRS IV – CE model assumptions..64
Table 58. CDLCI effectiveness– UPDRS I – data included in the analysis 65
Table 59. CDLCI effectiveness – UPDRS I – CE model assumptions..65
Table 60. CDLCI effectiveness– UPDRS II – data included in the analysis 65
Table 61. CDLCI effectiveness – UPDRS II – relative effectiveness (treatment initiation vs 1 year) data 66
Table 62. CDLCI effectiveness – UPDRS II – relative effectiveness (1 year vs 2 years) data 67
Table 63. CDLCI effectiveness – UPDRS II – CE model assumptions...67
Table 64. CDLCI effectiveness in clinical studies – UPDRS III – data included in the analysis..........................67
Table 65. CDLCI effectiveness – UPDRS III – relative effectiveness (treatment initiation vs 1 year) data 68
Table 66. CDLCI effectiveness – UPDRS III – relative effectiveness (1 year vs 2 years) data 69
Table 67. CDLCI effectiveness – UPDRS III – CE model assumptions..69
Table 68. CDLCI effectiveness– UPDRS IV – data included in the analysis...69
Table 69. CDLCI effectiveness – UPDRS IV – relative effectiveness (1 year vs 2 years) data 70
Table 70. CDLCI effectiveness – UPDRS IV – CE model assumptions 70
Table 71. Long-term UPDRS progression in BMT studies – data used in the analysis.....................................71
Table 72. DBS long-term effectiveness - UPDRS I – data used in the analysis 72
Table 73. DBS long-term effectiveness - UPDRS II (on med / on stim) – data used in the analysis 72
Table 74. DBS long-term effectiveness - UPDRS III (on med / on stim) – data used in the analysis 73
Table 75. DBS long-term effectiveness - UPDRS IV – data used in the analysis..73
Table 76. Long-term UPDRS progression – results from regression analyses in DBS studies 74
Table 77. Long-term UPDRS progression – results of the analysis..75
Table 78 Long-term UPDRS progression – model input data 76
Table 79. Adverse event (skin nodules / skin reaction) in clinical trials – CSAI treatment.................................77
Table 80. Occurrence of peritonitis during CDLCI treatment – data from clinical studies..................................78
Table 81. Discontinuation of CSAI treatment – data included in the analysis...78
Table 82. Discontinuation of CSAI treatment – model input data 79
Table 83. Discontinuation of CDLCI treatment – data included in the analysis 80
Table 84 Discontinuation of CDLCI treatment – model input data..81
Table 85. Daily doses of CSAI in clinical studies..82
Table 86. Characteristics of studies included in falls data analysis 83
Table 87. Falls frequency – data included in the analysis 85
Table 88. Frequency of injurious falls – data included in the analysis 86
Table 89. Association between UPDRS III and falls frequency: Odds ratio – data included in the analysis 86
Table 90 Data on falls in PD – model input data 86
Table 91. UK life tables for general adult population..87
Table 92 Association between UPDRS III and mortality – data included in the analysis and model input data 90

APPENDIX A. SYSTEMATIC REVIEWS

The following sections present detailed information on the systematic reviews of evidence that informed the economic analysis.

At all stages of each review the study selection was performed independently by two analysts. Verification at the level of abstracts and titles was carried out in such a way that all reports deemed useful by at least one of the analysts were enrolled to the next stage. In case of disagreements during the verification based on the full-text publications, the final decision on inclusion or exclusion was agreed by discussion and consensus.

Extraction of data from the studies included in the analysis was carried out by one of the analysts. Then the extraction was verified by a second analyst.

A.1. CSAI / CDLCI data

A.1.1. Search strategy

In order to identify data on effectiveness and safety of CSAI and CDLCI a systematic search was performed. Due to the fact that search was performed at the initial phase of the project, when model structure and data included in the analysis were unknown, broad inclusion criteria were defined (details in Table 4). Once the model structure was decided, selection criteria were narrowed to include only studies reporting CSAI/CDLCI effectiveness measured in terms of UPDRS scores.

Additionally, studies reporting data on safety, withdrawal and CSAI dose were selected from the studies initially included in full text analysis.

The following databases were searched for papers published from the inception of each database to 2015:

- Medline (via PubMed),
- Embase,
- The Cochrane Library.

The search was performed on 11th February 2015. Search strategies are outlined in Table 1, Table 2 and Table 3.

Table 3Table 1.
Search strategy in Medline database – CSAI / CDLCI effectiveness

Search	Search terms	Results
#1	Parkinson's disease	77 361
#2	"Parkinson Disease"[Mesh]	48 070
#3	Parkinson*	95 011
#4	parkinsonism OR (parkinsonian syndrome)	64 465
#5	paralysis agitans	57 763
#6	(#1 OR #2 OR #3 OR #4 OR #5)	96 372
#7	(apomorphine OR apomorfine)	11 324
#8	"Apomorphine"[Mesh]	8531
#9	“apomorphine hydrochloride”	95
#10	("Apo go" OR "Apo-go")	4
#11	Britaject OR Dacepton OR Apokyn OR Apokinon OR Ixense OR Spontane OR Uprima	11 339
#12	non-selective dopamine agonist	366
#13	#7 OR #8 OR #9 OR #10 OR #11 OR #12	11 586
#14	infusion	196 243
#15	continuous infusion	32 082
#16	CSI	2967
#17	subcutaneous	118 734
#18	(#14 OR #15 OR #16 OR #17)	310 377
#19	#13 AND #18	967
#20	Duodopa	478
#21	"carbidopa, levodopa drug combination"[Supplementary Concept]	446
#22	(carbidopa/levodopa) OR levocarb	157
#23	levodopa AND carbidopa	2207
#24	#20 OR #21 OR #22 OR #23	2224
#25	(infusion OR gel) OR (jejun* OR intrajejun* OR duoden* or intraduoden* OR intestinal OR intraintestinal OR enteral)	1 153 466
#26	#24 AND #25	230
#27	#19 OR #26	1161
#28	#6 AND #27	614
**Search strategy executed on 11th February 2015**		

Table 2.
Search strategy in Embase database – CSAI / CDLCI effectiveness

Search	Search terms	Results
**#1**	parkinsons AND ('disease'/exp OR disease) AND [embase]/lim	1022
**#2**	'parkinson disease'/exp OR 'parkinson disease' AND [embase]/lim	89 073
**#3**	parkinson* AND [embase]/lim	126 492
**#4**	'parkinsonism'/exp OR parkinsonism OR (parkinsonian AND ('syndrome'/exp OR syndrome)) AND [embase]/lim	29 576
**#5**	paralysis AND agitans AND [embase]/lim	105
**#6**	#1 OR #2 OR #3 OR #4 OR #5	126 522
**#7**	'apomorphine'/exp OR apomorphine OR 'apomorfine'/exp OR apomorfine AND [embase]/lim	17 499
**#8**	'apomorphine'/exp OR 'apomorphine' AND [embase]/lim	17 498
**#9**	'apomorphine hydrochloride'/exp OR 'apomorphine hydrochloride' AND [embase]/lim	16 478
**#10**	'apo go'/exp OR 'apo go' OR 'apo-go'/exp OR 'apo-go' AND [embase]/lim	16 470
**#11**	britaject OR dacepton OR 'apokyn'/exp OR apokyn OR 'apokinon'/exp OR apokinon OR 'ixense'/exp OR ixense OR spontane OR 'uprima'/exp OR uprima AND [embase]/lim	17 381
**#12**	'non selective' AND ('dopamine'/exp OR dopamine) AND ('agonist'/exp OR agonist) AND [embase]/lim	305
**#13**	#7 OR #8 OR #9 OR #10 OR #11 OR #12	18 605
**#14**	'infusion'/exp OR infusion AND [embase]/lim	257 682
**#15**	continuous AND ('infusion'/exp OR infusion) AND [embase]/lim	58 590
**#16**	csi AND [embase]/lim	4294
**#17**	'subcutaneous'/exp OR subcutaneous AND [embase]/lim	287 651
**#18**	#14 OR #15 OR #16 OR #17	531 563
**#19**	#13 AND #18	4189
**#20**	'duodopa'/exp OR duodopa AND [embase]/lim	4732
**#21**	levocarb AND [embase]/lim	6
**#22**	'levodopa'/exp OR levodopa AND ('carbidopa'/exp OR carbidopa) AND [embase]/lim	8167
**#23**	#20 OR #21 OR #22	8172
**#24**	'infusion'/exp OR infusion OR 'gel'/exp OR gel OR jejun* OR intrajejun* OR duoden* OR intraduoden* OR intestinal OR intraintestinal OR enteral AND [embase]/lim	879 414
**#25**	#23 AND #24	675
**#26**	#19 OR #25	4669
**#27**	#6 AND #26	1770
	**Search strategy executed on 11th February 2015**	

Table 3.
Search strategy in Cochrane Library database – CSAI / CDLCI effectiveness

Search	Search terms	Results
#1	Parkinson's disease	3415
#2	MeSH descriptor: [Parkinson Disease] explode all trees	2307
#3	Parkinson*	5017
#4	parkinsonism OR (parkinsonian syndrome)	1067
#5	paralysis agitans	4
#6	(#1 OR #2 OR #3 OR #4 OR #5)	5017
#7	(apomorphine OR apomorfine)	281
#8	MeSH descriptor: [Apomorphine] explode all trees	156
#9	"apomorphine hydrochloride"	18
#10	("Apo go" OR "Apo-go")	0
#11	Britaject OR Dacepton OR Apokyn OR Apokinon OR Ixense OR Spontane OR Uprima	52
#12	non-selective dopamine agonist	11
#13	#7 OR #8 OR #9 OR #10 OR #11 OR #12	341
#14	infusion	30 308
#15	continuous infusion	7975
#16	CSI	208
#17	subcutaneous	11 779
#18	(#14 OR #15 OR #16 OR #17)	40 615
#19	#13 AND #18	84
#20	Duodopa	3
#21	levocarb	0
#22	levodopa AND carbidopa	449
#23	#20 OR #21 OR #22	449
#24	(infusion OR gel) OR (jejun* OR intrajejun* OR duoden* or intraduoden* OR intestinal OR intraintestinal OR enteral)	54 220
#25	#23 AND #24	38
#26	#19 OR #25	120
#27	#6 AND #26	70
	**Search strategy executed on 11th February 2015**	

A.1.2. Studies selection

All studies identified within search process were initially assessed based on title and abstracts. Papers not meeting predefined inclusion criteria as indicated by title and abstracts were excluded.

In Table 4 below selection criteria defined according to PICO scheme are shown.

Table 4.
Selection criteria for clinical studies on CSAI / CDLCI effectiveness in Parkinson Disease

Criterion	Inclusion	Exclusion
**Population**	• Adult patients with Parkinson's disease	• Other
**Intervention**	• CSAI<br>• CDLCI	• Other
**Comparators**	• Not relevant	–
**Outcomes**	• Efficacy:<br>• *UPDRS score,*<br>• *Hoehn and Yahr scale,*<br>• *'off" state time,*<br>• *Dyskinesia,*<br>• *QoL measured by PDQ-39, PDQ-8, EQ-5D,*<br>• Safety, mortality.	• Lack of interesting outcomes
**Study design**	• RCT,<br>• Observational studies,<br>• Registries,<br>• Case series.	• Cross-over studies, if no results were presented before patients cross,<br>• Follow-up < 1 week,<br>• Case series or studies with < 10 patients.
**Other**	• Primary studies,<br>• Publications available in full-text,<br>• Publications in English.	• Reviews, systematic reviews, meta-analysis,<br>• Comments, editorials, letters,<br>• Conference abstracts (unless concern results of primary study independently identified in search).
**Publication date**	• Not relevant (up to date of the search - 11th February 2015)	–

Forty one studies (described in 49 publications) were included after full text analysis based on selection criteria. Additionally for 3 of finally included studies datasets on clinicaltrials.gov website for 3 studies were found and analysed. [1–6].

For the purposes of the economic analysis, additional selection was done and studies were excluded from final calculations of effectiveness due to one of the following reasons:

- no data on UPDRS or UPDRS not measured in ON-meds condition,
- insufficient data on UPDRS (only at baseline or no baseline values),
- results presented as medians,
- results presented at non-specified time points, e.g. after mean or median follow-up.

Studies were also included in final analysis if any of the following data was reported: CSAI mean daily dose, frequency of adverse events (specifically skin nodules / skin reaction for CSAI and peritonitis for CDLCI) and withdrawal rates.

A brief summary of all 3 searches and results of studies selection is presented in the table below. The sequential phases of the review process are also illustrated in the PRISMA flow diagram (Figure 1).

Table 5.
Summary of systematic search for CSAI / CDLCI effectiveness

Search date	Database	No. of abstracts returned	Initial selection		Final no. of included studies
			No. included in full text analysis	No. of included studies	
11th February 2015	Pubmed, Embase, Cochrane	2,454	280	41 studies<br>49 publications<br>3 additional from CT	30 studies<br>38 publications<br>3 additional from CT

CT – clinicaltrials.gov; in 3 studies data both for CSAI and CDLCI

Figure 1.
Overview of studies selection: PRISMA flowchart – CSAI/CDLCI data

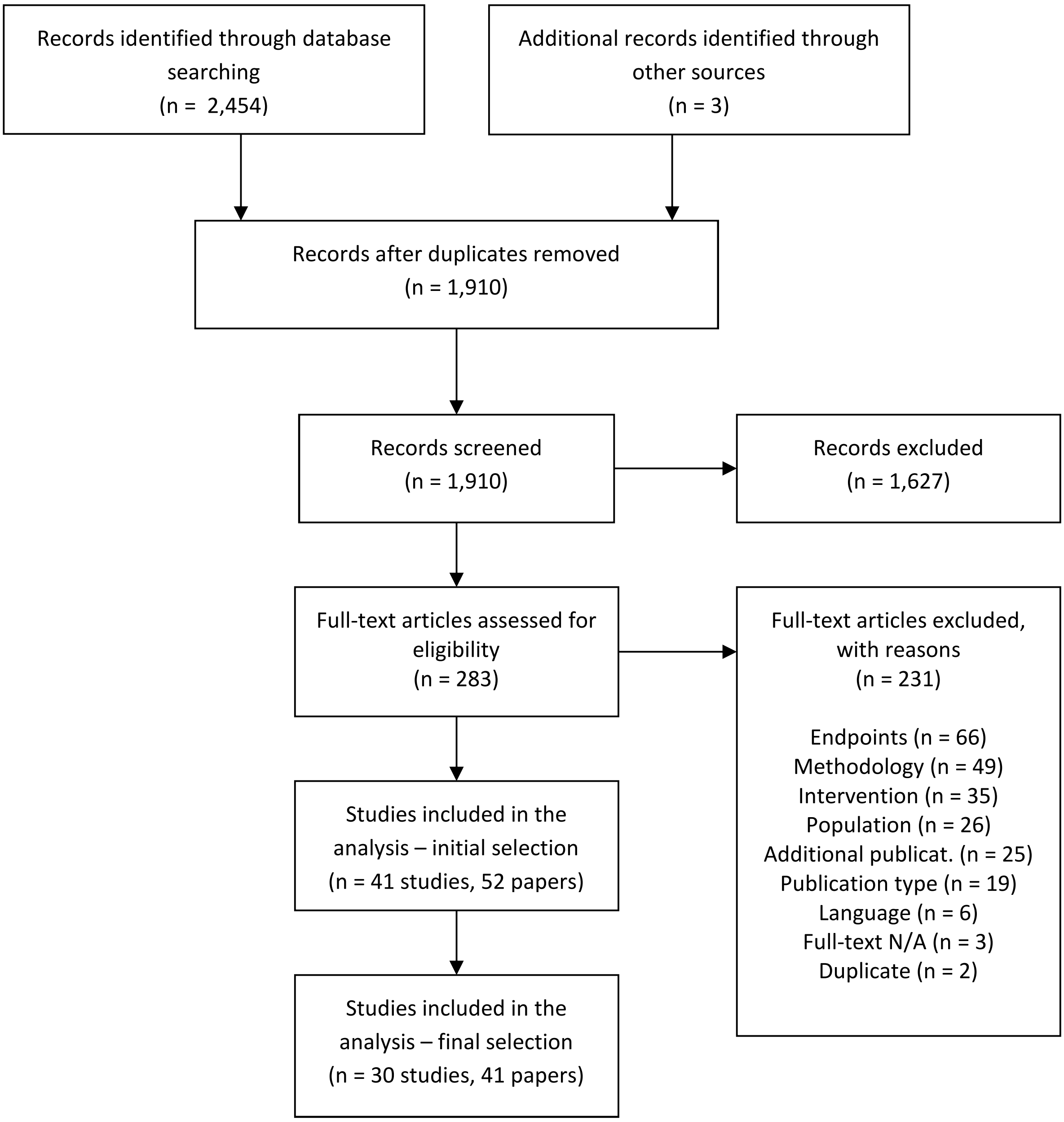

In Table 6 and Table 7 a brief summary of the type of data identified in all 41 initially included studies is presented. 16 studies were identified initially for CSAI, 23 for CDLCI and 2 studies reported both

CSAI and CDLCI data (Elia 2012 [7], Martinez-Martin 2014 [8]). No long term (follow-up longer than 2 years) effectiveness studies for CSAI and CDLCI meeting inclusion criteria were identified.

The list of studies included in final analysis for particular data categories together with data extracted from the studies are presented in the following sections:

- CSAI effectiveness – section A.1.3.1,
- other CSAI parameters – section A.1.3.2,
- CDLCI effectiveness – section A.1.3.3,
- other CDLCI parameters – section A.1.3.4.

Table 6.
Studies initially included in the analysis and final selection – CSAI

Study	Initial selection	Final selection		
		Effectiveness	Safety[b]	Other
**Colzi 1998 [9]**	off-time, dyskinesia	OUT: (no UPDRS)	OUT	-
**De Gaspari 2006 / Antonini 2011 [10, 11]**	UPDRS, off-time, safety	IN[a]	IN	APO dose, treatment withdrawal
**Drapier 2012 [12]**	UPDRS, H&Y, dyskinesia, safety	IN	IN	APO dose, treatment withdrawal
**Elia 2012 [7]**	UPDRS, off-time, safety	OUT (mean follow-up)	IN	-
**Garcia-Ruiz 2008 [13]**	UPDRS, off-time, dyskinesia, safety	OUT (mean follow-up)	IN	APO dose
**Hughes 1993 / Frankel 1990 [14, 15]**	off-time, safety	OUT: (no UPDRS)	IN	APO dose, treatment withdrawal
**Kanovsky 2002 [16]**	UPDRS, off-time, dyskinesia, safety	IN for IV<br>OUT for III[c]	OUT	-
**Katzenschlager 2005 [17]**	off-time, dyskinesia, safety	OUT: (no UPDRS)	IN	APO dose, treatment withdrawal
**Manson 2002 [18]**	off-time, dyskinesia, safety	OUT: (no UPDRS)	OUT	APO dose, treatment withdrawal
**Martinez-Martin 2011 [19]**	UPDRS, Qol	IN for IV<br>OUT for III[c]	OUT	-
**Martinez-Martin 2014 [8]**	UPDRS, Qol, safety		OUT	APO dose, treatment withdrawal
**Morgante 2004 / di Rosa 2003 [20, 21]**	off-time, safety	OUT: (no UPDRS)	IN	APO dose, treatment withdrawal
**Pietz 1998 [22]**	H&Y, off-time, dyskinesia, safety	OUT: (no UPDRS)	IN	-
**Pinter 1998 [23]**	off-time	OUT: (no UPDRS)	OUT	-
**Poewe 1993 [24, 25]**	off-time, safety	OUT: (no UPDRS)	IN	APO dose, treatment withdrawal
**Stibe 1988 [26]**	off-time, safety	OUT: (no UPDRS)	IN	APO dose
**Stocchi 2001 [27]**	dyskinesia, safety	OUT: (no UPDRS)	OUT	APO dose
**Todorova 2013 [28]**	UPDRS, Qol, safety	IN	OUT	APO dose, treatment withdrawal

a) only 1 year data; additional results after mean follow-up presented; b) included if detailed data on skin nodules occurrence reported; c) unclear whether UPDRS III was measured ON-meds or OFF-meds

Table 7.
Studies initially included in the analysis and final selection – CDLCI

Study	Available data	Inclusion in final CUA		
		Effectiveness [a]	Safety	Other
**Antonini 2008 [29]**	UPDRS, off-time, dyskinesia, Qol	IN for III-IV<br>OUT for II[b]	OUT	-
**Antonini 2013 [30]**	UPDRS, off-time, dyskinesia, Qol, safety	IN	IN	-
**Antonini 2014 (GLORIA) [31]**	UPDRS, off-time, dyskinesia, Qol, safety	IN	OUT	treatment withdrawal
**Caceres-Redondo 2014 [32]**	UPDRS, off-time, dyskinesia, Qol, safety	IN	OUT	-
**Devos 2009 [33]**	UPDRS, H&Y, safety	OUT (insufficient data)	IN	-
**Eggert 2008 [34]**	off-time, dyskinesia, safety	OUT: (no UPDRS)	OUT	treatment withdrawal
**Elia 2012 [7]**	UPDRS, off-time, safety	OUT (mean follow-up)	OUT	-
**Fasano 2012 [35]**	UPDRS, off-time, Qol, safety	OUT (mean follow-up)	OUT	-
**Fernandez 2014 [1, 4, 36, 37]**	UPDRS, off-time, dyskinesia, Qol, safety	IN for I–III<br>OUT for IV (mean follow-up)	IN	treatment withdrawal
**Foltynie 2013 [38]**	off-time, dyskinesia, Qol, safety	OUT: (no UPDRS)	OUT	-
**Honig 2009 [39]**	UPDRS, off-time, Qol	IN	OUT	-
**Isacson 2008 [40]**	off-time, Qol	OUT: (no UPDRS)	OUT	-
**Karlsborg 2010 [41]**	UPDRS, safety	IN	OUT	-
**Lundqvist 2014 [42]**	UPDRS, H&Y, off-time, dyskinesia, safety, Qol	OUT: (no UPDRS)	OUT	-
**Martinez-Martin 2014 [8]**	UPDRS, Qol, safety	IN for IV<br>OUT for III[b]	IN	-
**Meppelink 2011 [43]**	UPDRS, Qol, safety	OUT (unclear measure condition)	OUT	-
**Merola 2011 [44]**	UPDRS, off-time, dyskinesia, safety	OUT (mean follow-up)	OUT	-
**Nyholm 2008 [45]**	dyskinesia, safety	OUT: (no UPDRS)	IN	treatment withdrawal

Study	Available data	Inclusion in final CUA		
		Effectiveness [a]	Safety	Other
**Olanow 2014 / Slevin 2015 [2, 3, 5, 6]**	UPDRS, off-time, dyskinesia, Qol, safety	IN for I–III<br>OUT for IV (insufficient data)	OUT	treatment withdrawal
**Palhagen 2012 [46]**	UPDRS, Qol, safety	IN for III-IV<br>OUT for II[b]	IN	-
**Pickut 2014 [47]**	UPDRS, H&Y, safety	OUT (mean follow-up)	OUT	-
**Sensi 2014 [48]**	UPDRS, H&Y, off-time, safety	IN	IN	-
**Zibetti 2013a [49]**	UPDRS	OUT (mean follow-up)	OUT	-
**Zibetti 2013b [50]**	UPDRS, Qol, safety	OUT (mean follow-up)	OUT	-
**Zibetti 2014 [51]**	UPDRS, off-time, dyskinesia, Qol, safety	OUT (mean follow-up)	IN	-

a) only data for specified time point are included; in some studies also other time point results available; b) due to unclear measurement condition – it was not reported if measured within ON med state

A.1.3. Data extraction

A.1.3.1 CSAI – effectiveness (UPDRS)

Studies included in the analysis for particular UPDRS scores are listed in Table 8. A brief description of the studies is provided in Table 9.

Table 8
CSAI studies included in final analysis – short-term effectiveness

Parameter	Short-term effectiveness			
	UPDRS I	UPDRS II	UPDRS III	UPDRS IV
**Publications included**	Drapier 2012	Drapier 2012	Drapier 2012,<br>de Gaspari 2006<br>Todorowa 2013	Drapier 2012<br>Kanovsky 2002<br>Martinez-Martin 2011<br>Martinez-Martin 2014<br>Todorowa 2013
**Total number of publications / studies**	**1**	**1**	**3**	**5**

Table 9
Study characteristic – CSAI short-term effectiveness

Study	Study design	Follow-up	No of patients	Baseline patients characteristics			
				Age [mean (SD)]	Duration of PD [years (SD)]	Hoehn & Yahr (SD)	LEDD [mg (SD)]
**de Gaspari 2006**	**Design:** nonrandomized<br>**Aim:** To compare clinical and neuropsychological outcome following CSAI and STN-DBS in advanced PD patients<br>**Population:** Italy<br>**Comparator:** STN-DBS	12 months	13	59 (13)	10 (5)	≥3	665.98 (215)
**Drapier 2012**	**Design:** nonrandomized, prospective<br>**Aim:** to evaluate the efficacy and cognitive safety of CSAI<br>**Population:** France	12 months	23	48.4 (10.5)	13.9 (8.2)	ON: 2.3 (0.9)<br>OFF: 4.2 (1.0)	1 372.2 (325.1)
**Kanovsky 2002**	**Design:** nonrandomized<br>**Aim:** To assess the effect of smaller amounts of CSAI<br>**Population:** Czech Republic	2 years	12	64.3 (9.2)	14.4 (6.3)	4.5	1 650 (570)
**Martinez-Martin 2011**	**Design:** nonrandomized, multicenter, observational<br>**Aim:** Assessing effects of CSAI therapy on non-motor symptoms and health-related quality of life<br>**Population:** European<br>**Comparator:** control (best conventional therapy)	Mean (SD): 12.5 (11.5) months	17	59.5 (11.7)	12.05 (4)	Median 4	1077.81 (446.26)

Study	Study design	Follow-up	No of patients	Baseline patients characteristics			
				Age [mean (SD)]	Duration of PD [years (SD)]	Hoehn & Yahr (SD)	LEDD [mg (SD)]
**Martinez-Martin 2014**	**Design:** nonrandomized, prospective, multicenter, observational<br>**Aim:** Comparison of CSAI with CDLCI using validated motor, nonmotor, and quality-of-life outcome measures.<br>**Population:** European<br>**Comparator:** CDLCI	6 months	43	62.3 (10.6)	14 (4.4)	Median 3	1 934 (374)
**Todorova 2013**	**Design:** nonrandomized, prospective<br>**Aim:** To extend the beneficial effect of 12–14 h waking day CSAI to 24 h therapy by combining with Rotigotine transdermal patch therapy and assess motor and non-motor effects<br>**Population:** United Kingdom	2 years	15	60.3 (11.3)	15.3 (4.8)	ON:<br>8 patients: 3<br>7 patients: 4	Not reported

The data extracted from the studies finally included in the analysis (UPDRS scores and number of patients at different follow-up time points) together with percentage UPDRS changes (versus baseline) calculated for the purposes of the economic analysis are presented in Table 10.

Table 10.
CSAI effectiveness – UPDRS – data extraction

Study	UPDRS score / % change vs baseline (Number of patients)			
	Baseline	6 months	1 year	2 years
**UPDRS I**				
**Drapier 2012**	2.90 (23)		2.40 / -17.24% (23)	
**UPDRS II (on meds)**				
**Drapier 2012**	10.20 (23)		11.00 / 7.84% (23)	
**UPDRS III (on meds)**				
**Drapier 2012**	18.30 (23)		21.80 / 19.1% (23)	
**De Gaspari 2006**	19.50 (13)		19.25 / -1.3% (13)	
**Todorowa 2013**	33.00 (20)	-		14.50 / -56.1% (15)

Study	UPDRS score / % change vs baseline (Number of patients)			
	Baseline	6 months	1 year	2 years
UPDRS IV				
**Drapier 2012**	7.70 (23)	-	6.90 / -10.4% (23)	-
**Kanovsky 2002**	10.80 (12)	5.40 / -50.0% (12)	5.30 / -50.9% (12)	5.40 / -50.0% (12)
**Martinez-Martin 2011**	10.00 (17)	-	3.53 / -64.7% (17)	-
**Martinez-Martin 2014**	10.02 (43)	5.93 / -40.8% (43)	-	-
**Todorowa 2013**	11.30 (20)	-	-	5.20 / -54.0% (15)

Detailed information on further data analysis and final model assumptions are presented in section B.1.1.

A.1.3.2 CSAI – other parameters

Studies included in the analysis for particular data categories are listed in Table 11.

Table 11
CSAI studies included in final analysis – other parameters

Parameter	Withdrawal	Adverse effects	Dose
**Publications included**	Antonini 2011 / de Gaspari 2006<br>di Rosa 2003 / Morgante 2004<br>Drapier 2012<br>Frankel 1990 / Hughes 1993<br>Katzenschlager 2005<br>Manson 2002<br>Martinez-Martin 2014<br>Poewe 1993<br>Todorova 2013	Antonini 2011 / de Gaspari 2006<br>di Rosa 2003 / Morgante 2004<br>Drapier 2012<br>Elia 2012<br>Frankel 1990 / Hughes 1993<br>Garcia-Ruiz 2008<br>Katzenschlager 2005<br>Pietz 1998<br>Poewe 1993<br>Stibe 1988	Antonini 2011 / de Gaspari 2006<br>di Rosa 2003 / Morgante 2004<br>Drapier 2012<br>Frankel 1990 / Hughes 1993<br>Garcia-Ruiz 2008<br>Katzenschlager 2005<br>Manson 2002<br>Martinez-Martin 2014<br>Poewe 1993<br>Stibe 1988<br>Stocchi 2001<br>Todorova 2013
**Total number of publications/studies**	**12/9**	**13/10**	**15/12**

Withdrawal data

The data extracted from the studies included in the analysis is presented in Table 12. Detailed information on final model assumptions is presented in section B.4.1.

Table 12.
CSAI withdrawal – data extraction

Study	Total number of patients	Discontinuation		Follow-up [years]	Withdrawal reasons
		Number of patients	% of patients		
**Antonini 2011 / de Gaspari 2006**	12	6	50.0%	5.00 / 2.50 [a / b]	AEs or no effectiveness
**Drapier 2012**	23	0	0.0%	1.00 [a]	-
**Hughes 1993 / Frankel 1990**	25	6	24.0%	3.04 [b]	AEs (4), other (2)
**Katzenschlager 2005**	12	0	0.0%	0.50 [a]	-
**Manson 2002**	64	8	12.5%	2.82 [b]	AEs (3), patient's decision (2), unable to continue (3)
**Martinez-Martin 2014**	43	0	0.0%	0.50 [a]	-
**Morgante 2004 / di Rosa 2003**	12	1	8.3%	2.00 [a]	Infection
**Poewe 1993**	18	4	22.2%	1.72 [b]	AEs (3), no effectiveness (1)
**Todorova 2013**	20	5	25.0%	2.00 [a]	Skin reaction

a) total follow-up of the study; b) mean follow-up

Adverse events data

Data on skin reaction / skin nodules frequency was retrieved from the studies identified in the systematic review, as these were the most common adverse event for CSAI treatment reported in the literature. Other adverse events reported in the literature were not included in the model, as they were considered too rare (occurring in less than 5% of patients) or not influencing patient's quality of life or cost-generating.

The data extracted from the studies included in the analysis (only studies reporting frequency of skin nodules / skin reaction) is presented in Table 13. Detailed information on final model assumptions is presented in section B.3.1.

Table 13.
CSAI related adverse events – skin nodules / skin reaction – data extraction

Study	Number of patients	Patients with events	% with events	Comment
**Antonini 2011 / de Gaspari 2006**	12	2	17%	Based on Antonini 2011
**Drapier 2012**	23	23	100%	-
**Elia 2012**	10	5	50%	-
**Garcia-Ruiz 2008**	82	56	68%	In 7 patients with severe grade
**Hughes 1993 / Frankel 1990**	25	25	100%	Based on Frankel 1990
**Katzenschlager 2005**	12	9	75%	-
**Morgante 2004 / di Rosa 2003**	10	10	100%	Based on Morgante 2004

Study	Number of patients	Patients with events	% with events	Comment
Pietz 1998	25	25	100%	-
Poewe 1993	18	4	22%	-
Stibe 1988	11	11	100%	-

Additionally in Stocchi 1993 , Stocchi 2001 and Manson 2002 skin nodules / skin reaction occurred in almost all patients but no detailed data were presented

Dosage data

The data extracted from the studies included in the analysis is presented in Table 14. Detailed information on final model assumptions is presented in section B.5.

Table 14.
Daily doses of CSAI – data extraction

Study	Number of patients	Dose per day [mg]		Comment
		Mean	SD	
Antonini 2011 / de Gaspari 2006	12	83.40	19.20	-
Drapier 2012	23	62.60	18.80	-
Garcia-Ruiz 2008	82	72.00	21.38	-
Hughes 1993 / Frankel 1990	22	80.80	31.60[a]	data from Hughes 1993 taken; mean dose after initial stabilization
Katzenschlager 2005	8	84.70	33.13[a]	data form UK patients group
Manson 2002	45	102.50	71.80	data from monotherapy group
Martinez-Martin 2014	43	105.90	23.20	-
Morgante 2004 / di Rosa 2003	12	100.00	39.11[a]	-
Poewe 1993	14	160.00	62.58[a]	data from patients with complete follow-up
Stibe 1988	11	77.00	30.12[a]	-
Stocchi 2001	30	51.60	34.80	-
Todorova 2013	20	105.30	23.90	-

a) SD value not reported within the study; calculated based on relation of SD to mean from other studies (39.11%)

A.1.3.3 CDLCI – effectiveness

Studies included in the analysis for particular UPDRS scores are listed in Table 15. Brief description of the studies is provided in Table 16.

Table 15
CDLCI studies included in final analysis – short-term effectiveness

Parameter	Short-term effectiveness			
	UPDRS I	UPDRS II	UPDRS III	UPDRS IV
**Publications included**	Fernandez 2014<br>Olanov 2014 / Slevin 2015	Antonini 2013<br>Antonini 2014 (GLORIA)<br>Caceres-Redondo 2014<br>Fernandez 2014<br>Olanov 2014 / Slevin 2015	Antonini 2008<br>Antonini 2013<br>Antonini 2014 (GLORIA)<br>Caceres-Redondo 2014<br>Fernandez 2014<br>Honig 2009<br>Karlsborg 2010<br>Olanow 2014 / Slevin 2015<br>Palhagen 2012<br>Sensi 2014	Antonini 2008<br>Caceres-Redondo 2014<br>Honig 2009<br>Martinez-Martin 2014<br>Palhagen 2012<br>Sensi 2014
**Total number of publications/studies**	**3/2**	**6/5**	**11/10**	**6**

Table 16
Study characteristic – CDLCI short-term effectiveness

Study	Study design	Follow-up	No of patients	Baseline patients characteristics			
				Age [mean (SD)]	Duration of PD [years (SD)]	Hoehn & Yahr (SD)	LEDD [mg (SD)]
**Antonini 2008**	**Design:** nonrandomized, prospective, multicenter<br>**Aim:** To assess the effectiveness of CDLCI on quality of life as well as motor features in patients with advanced PD<br>**Population:** Italy	2 years	22	Not reported	Not reported	≥3	Not reported
**Antonini 2013**	**Design:** nonrandomized, retrospective, multicenter, cohort<br>**Aim:** Assessment of long-term safety and outcome of chronic treatment with CDLCI **Population:** European	Up to 2 years	98	68.9 (14.6)	14.9 (6.6)	OFF: 3.9 (1.0)	973 (636)
**Antonini 2014 (GLORIA)**	**Design:** nonrandomized, observarional, multicenter<br>**Aim:** To record long-term effectiveness of advanced PD patients undergoing CDLCI infusion<br>**Population:** 18 countries (17 european and Australia)	1 year	172	66.5 (9.3)	12.6 (6.6)	2.8 (0.8)	Not reported
**Caceres-Redondo 2014**	**Design:** nonrandomized, single center<br>**Aim:** investigate the motor and cognitive outcome of CDLCI treatment in advanced PD<br>**Population:** Spain	2 years	16	64.5 (9.0)	14.1 (3.9)	ON: 2.4 (0.5)<br>OFF: 3.7 (0.8)	1 306.1 (337.6)

Study	Study design	Follow-up	No of patients	Baseline patients characteristics			
				Age [mean (SD)]	Duration of PD [years (SD)]	Hoehn & Yahr (SD)	LEDD [mg (SD)]
**Fernandez 2014**	**Design:** nonrandomized, prospective, multicenter **Aim:** To provide long-term safety and efficacy data for advanced PD patients **Population:** 16 countries	54 weeks	354	64.1 (9.1)	12.5 (5.5)	Not reported	1 082.9 (582.1)
**Honig 2009**	**Design:** nonrandomized, prospective, observational, multicenter **Aim:** Assessing effects of CDLCI on nonmotor symptoms of advanced PD and on the relationship with motor changes and health related quality of life **Population:** United Kingdom, Germany, Italy	6 months	22	58.6 (9.1)	15.3 (5.9)	OFF: 3.75 (0.84)	Not reported
**Karlsborg 2010**	**Design:** nonrandomized **Aim:** To report the effect of Duodopa treatment on PD patients' "on" state as measured by the UPDRS III and their motor fluctuations and dyskinesias. **Population:** Denmark	Mean for 12 patients: 16.3 (15.7)	14	64.9 (13.5)	16.2 (7.4)	3.3 (0.7)	1316.5 (845.7)
**Martinez-Martin 2014**	**Design:** nonrandomized, prospective, multicenter, observational **Aim:** Comparison of CSAI with CDLCI using validate motor, nonmotor, and quality-of-life outcome measures. **Population:** European **Comparator:** CSAI	6 months	44	62.7 (9.1)	16.1 (6.7)	Median: 4	2 017 (857.2)
**Olanow 2014**	**Design:** RCT, prospective, multicentre, **Aim:** To assess safety and efficacy of CDLCI in patients with advanced PD **Population:** Germany, New Zealand, USA **Comparator:** placebo	12 weeks	37	63.7 (9.5)	10.0 (4.6)	Not reported	1005.4 (373.6)
**Slevin 2015**	**Design:** open-label extension of RCT **Aim:** To examine long-term safety, efficacy and quality of life of CDLCI **Population:** Germany, New Zealand, USA **Comparator:** placebo	1 year	33	63.6 (9.0)	10.07 (4.84)	Not reported	Not reported
**Palhagen 2012**	**Design:** nonrandomized, prospective **Aim:** To investigate clinical and health-related quality of life effects of CDLCI **Population:** Norway, Sweden	12 months	27	64.6 (6.4)	12.6	Not reported	Not reported

Study	Study design	Follow-up	No of patients	Baseline patients characteristics			
				Age [mean (SD)]	Duration of PD [years (SD)]	Hoehn & Yahr (SD)	LEDD [mg (SD)]
**Sensi 2014**	**Design:** nonrandomized, prospective, single center<br>**Aim:** To detect any predictive factor to identify the best candidates for CDLCI therapy<br>**Population:** Italy	32.47 (9.47) months	17	67.6 (6.1)	15.47 (4.04)	3.17 (0.8)	1158.9 (334.5)

The data extracted from the studies included in the analysis (UPDRS scores and number of patients at different follow-up time points) together with percentage UPDRS changes (versus baseline) calculated for the purposes of the economic analysis are presented in Table 17.

Table 17.
CDLCI effectiveness – UPDRS – data extraction

Study	UPDRS score / % change vs baseline (Number of patients)					
	Baseline	4 weeks	12 weeks	6 months	1 year	2 years
**UPDRS I**						
**Fernandez 2014**	2.20 (288)	-	-	-	2.20 / 0.00% (272[a])	-
**Olanov 2014 / Slevin 2015[b]**	1.80 (36)	-	1.60 / -11.1% (36)	-	2.10 / 16.7% (33)	-
**Olanov 2014 / Slevin 2015[c]**	1.80 (33)	-	-	-	2.50 / 38.9% (26)	-
**UPDRS II (on meds)**						
**Antonini 2013**	14.78 (73)	-	-	10.64 / -28.1% (53)	11.83 / -20.0% (43)	13.95 / -5.7% (33)
**Antonini 2014 (GLORIA)**	16.50 (172)	-	-	14.40 / 12.7% (69)	13.40 / -18.8% (56)	-
**Caceres-Redondo 2014**	14.50 (16)	-	-	-	-	16.50 / 13.8% (16)
**Fernandez 2014**	17.40 (293)	11.80 / -32.2% (286)	12.00 / -31.0% (279)	12.10 / -30.5% (269)	13.20 / -24.1% (251)	-
**Olanov 2014 / Slevin 2015[b]**	11.60 (36)	-	9.80 / -15.5% (35)	-	12.10 / 4.3% (33)	-
**Olanov 2014 / Slevin 2015[c]**	11.80 (33)	-	-	-	10.80 / -8.5% (26)	-

Study	UPDRS score / % change vs baseline (Number of patients)					
	Baseline	4 weeks	12 weeks	6 months	1 year	2 years
**UPDRS III (on meds)**						
**Antonini 2008**	24.60 (22)	-	-	-	23.80<br>-3.3% (22)	24.80<br>0.8% (22)
**Antonini 2013**	25.34 (73)	-	-	22.58<br>-10.9% (55)	23.33<br>-7.9% (47)	27.05<br>6.7% (29)
**Antonini 2014 (GLORIA)**	26.50 (172)	-	-	23.50<br>-11.3% (87)	23.20<br>-12.5% (74)	-
**Caceres-Redondo 2014**	27.20 (16)	-	-	-	-	29.50<br>8.5% (16)
**Fernandez 2014**	28.80 (291)	19.00<br>-34.0% (286)	20.00<br>-30.6% (279)	19.70<br>-31.6% (269)	20.80<br>-27.8% (251)	-
**Honig 2009**	19.10 (22)	-	-	11.60<br>-39.3% (22)	-	-
**Karlsborg 2010**	36.80 (12)	20.40<br>-44.6% (12)	-	-	-	-
**Olanow 2014 / Slevin 2015[b]**	18.10 (36)	-	16.60<br>-8.3% (35)	-	19.60<br>8.3% (33)	-
**Olanow 2014 / Slevin 2015[c]**	22.50 (33)	-	-	-	22.00<br>-2.2% (25)	-
**Palhagen 2012**	24.40 (27)	-	-	-	21.50<br>-11.9% (25)	-
**Sensi 2014**	35.50 (17)	-	-	33.40<br>-5.9% (17)	-	-
**UPDRS IV**						
**Antonini 2008**	8.40 (22)	-	-	-	6.40<br>-23.8% (22)	6.60<br>-21.4% (22)
**Caceres-Redondo 2014**	8.70 (16)	-	-	-	-	6.70<br>-23.0% (16)
**Honig 2009**	10.50 (22)	-	-	4.50<br>-57.1% (22)	-	-
**Martinez-Martin 2014**	9.93 (44)	-	-	4.36<br>-56.1% (44)	-	-
**Palhagen 2012**	9.40 (27)	-	-	-	5.70<br>-39.4% (25)	-

Study	UPDRS score<br>% change vs baseline (Number of patients)					
	Baseline	4 weeks	12 weeks	6 months	1 year	2 years
**Sensi 2014**	8.40 (17)	-	-	5.60<br>-33.3% (17)	-	-

a) number of patients not reported, assumed the same as number of patients that completed 1-year follow-up;
b) patients randomized to CDLCI at baseline; c) patients randomized to BMT at baseline who started CDLCI after 12 weeks

Detailed information on further data analysis and final model assumptions are presented in section B.1.2.

A.1.3.4 CDLCI – other parameters

Studies included in the analysis for particular data categories are listed in Table 18.

Table 18
CDLCI studies included in final analysis – other parameters

Parameter	Withdrawal	Adverse effects
**Publications included**	Antonini 2014 (GLORIA)<br>Eggert 2008<br>Fernandez 2014<br>Nyholm 2008<br>Palhagen 2012<br>Slevin 2005	Antonini 2013d<br>Devos 2009<br>Fernandez 2014<br>Martinez-Martin 2014<br>Palhagan 2012<br>Sensi 2014<br>Zibetti 2014
**Total number of publications**	**6**	**7**

Withdrawal data

The data extracted from the studies included in the analysis is presented in Table 19. Detailed information on final model assumptions is presented in section B.4.2.

Table 19.
CDLCI withdrawal – data extraction

Study	Total number of patients	Discontinuation		Comment
		Number of patients	% of patients	
**Test phase / initial treatment period**				
**Antonini 2014 (GLORIA)**	172	8	8.5%	**Test phase:** run-in period – CDLCI infusion via nasoduodenal tube<br>**Discontinuation reason:** not reported in details
**Fernandez 2014**	354	22[a]	6.2%	**Test phase:** nasojejunal (NJ) titration period (2-14 days) and a PEG-J titration period (2-14 days)<br>**Discontinuation reason:** withdrew consent (12), AEs (5), lack of efficacy (5)

Study	Total number of patients	Discontinuation		Comment
		Number of patients	% of patients	
**Nyholm 2008**	58	7	12.1%	**Test phase:** nasodoudenal test period for an average 12 days (range: 3-30)<br>**Discontinuation reason:** not reported in details
**Palhagen 2012**	37	10	27.0%	**Test phase:** initiation nasoduodenal CDLCI + period before permanent CDLCI implantation (3 months)<br>**Discontinuation reason:** withdrew consent (3), AEs (2), lack of efficacy (5)
**1st year of treatment**				
**Eggert 2008**	13	4	30.8%	**Discontinuation reason:** patient's refusing (1), AEs (1), mechanical and physical problems (2)
**Fernandez 2014**	324	37[b]	16.0%	**Discontinuation reason:** withdrew consent (13), AEs (22), lack of efficacy (2)
**Slevin 2005**	29	5	17.2%	**Discontinuation reason:** withdrew consent (13), AEs (22), lack of efficacy (2)
**Subsequent years**				
**Nyholm 2012**	135	31	23.0%[c]	**Discontinuation reason:** AEs or lack of effectiveness<br>**Time period:** mean follow-up 4.2 years; restricted mean treatment time estimated with Kaplan–Meier methodology (censoring at the end of the study or death): 7.79 years

a) additional 8 patients withdrew due to administrative reason or protocol violation. According to methodology not included in calculation
b) additional 15 patients withdrew due to administrative reason or protocol violation. According to methodology not included in calculation
c) cumulative discontinuation rate for the whole follow-up period

Adverse events data

Data on frequency of peritonitis was retrieved from the systematic review, presented in Table 20. Detailed information on final model assumptions is presented in section B.3.2.

Table 20.
CDLCI related adverse events – peritonitis – data extraction

Study	Number of patients	Peritonitis	
		Number of events	Probability of event
**Antonini 2013d**	98	4	4.08%
**Devos 2009**	91	4	4.40%
**Fernandez 2014**	324	9	2.78%
**Martinez-Martin 2014**	44	1	2.27%
**Palhagan 2012**	37	1	2.70%
**Sensi 2014**	28	2	7.14%
**Zibetti 2014**	59	1	1.69%

A.2. Long-term DBS effectiveness

A.2.1. Search strategy

In order to identify data on long-term effectiveness of DBS a systematic literature search was performed. Due to the fact that search was performed at the initial phase of the project, when model structure and data included in the analysis were unknown, broad inclusion criteria were defined (details in Table 24). Once the model structure was decided, selection criteria were narrowed to include only studies reporting long-term DBS effectiveness measured in terms of UPDRS scores.

The following databases were searched for papers published from the inception of each database to 2015:

- Medline (via PubMed),
- Embase,
- The Cochrane Library.

The search was performed on 10th March 2015. Search strategies are outlined in Table 21, Table 22 and Table 23.

Table 21.
Search strategy in Medline database – DBS effectiveness

Search	Search terms	Results
1	Parkinson's disease	77779
2	"Parkinson Disease"[Mesh]	48251
3	Parkinson*	95489
4	(parkinsonism OR (parkinsonian syndrome))	64722
5	paralysis agitans	58046
6	#1 OR #2 OR #3 OR #4 OR #5	96856
7	"Deep Brain Stimulation"[Mesh]	4871
8	DBS	5568
9	globus pallidus interna stimulation	154
10	subthalamic nucleus stimulation	3206
11	#7 OR #8 OR #9 OR #10	8956
12	#6 AND #11	4025
13	((((Control or prospective or comparative* OR cohort* OR "cohort studies" OR (cohort and (study OR studies)) OR (cohort analy*) OR retrospectiv* OR observational OR (observational AND (study OR studies)) OR "cross-sectional" OR "cross sectional" OR "cross sectional" OR "cross-sectional studies" OR "case control studies" OR "Case control" OR ("follow up" and (study OR studies)) OR longitudinal Or retrospective OR (clinical AND (study OR trial)) OR crossover OR "cross-over" OR population-based OR survey OR register OR registry OR "case series"))))	7601004
14	((((("randomized controlled trial") OR (random*) OR (RCT) OR (((singl* OR doubl* OR trebl* OR tripl*) AND (blind* OR mask*)) OR (single blind) OR (double blind) OR (triple blind) OR (placebo) OR (placebo-controlled) OR (blinding) OR (controlled clinical trial) OR (random* AND controlled AND study*) OR (random* AND controlled AND trial*) OR ((random OR randomly) AND (allocation OR allocate*))))))))	1100157

Search	Search terms	Results
15	#13 OR #14	7836229
16	#12 AND #15	2190
**Search strategy executed on 10th March 2015**		

Table 22.
Search strategy in Embase database – DBS effectiveness

Search	Search terms	Results
1	parkinsons AND ('disease' OR 'disease'/exp OR disease) AND [embase]/lim	**1,062**
2	'parkinson disease'/exp OR 'parkinson disease' AND [embase]/lim	89,484
3	parkinson* AND [embase]/lim	**127,144**
4	'parkinsonism' OR 'parkinsonism'/exp OR parkinsonism OR (parkinsonian AND ('syndrome' OR 'syndrome'/exp OR syndrome)) AND [embase]/lim	29,705
5	'paralysis'/exp OR paralysis AND agitans AND [embase]/lim	105
6	#1 OR #2 OR #3 OR #4 OR #5	127,174
7	dbs AND [embase]/lim	8,692
8	'deep brain stimulation'/exp AND [embase]/lim	27,513
9	globus AND pallidus AND interna AND ('stimulation'/exp OR stimulation)	313
10	subthalamic AND nucleus AND ('stimulation'/exp OR stimulation)	5,018
11	#7 OR #8 OR #9 OR #10	31,357
12	#6 AND #11	8,227
13	control OR prospective OR comparative* OR cohort* OR 'cohort studies' OR (cohort AND (study OR studies)) OR (cohort AND analy*) OR retrospectiv* OR observational OR (observational AND (study OR studies)) OR 'cross-sectional' OR 'cross sectional' OR 'cross-sectional studies' OR 'case control studies' OR 'case control' OR ('follow up' AND (study OR studies)) OR longitudinal OR retrospective OR (clinical AND (study OR trial)) OR crossover OR 'cross-over' OR 'population based' OR survey OR register OR registry OR 'case series'	9,546,035
14	'randomized controlled trial' OR random* OR rct OR (singl* OR doubl* OR trebl* OR tripl* AND (blind* OR mask*)) OR (single AND blind) OR (double AND blind) OR (triple AND blind) OR placebo OR 'placebo controlled' OR blinding OR (controlled AND clinical AND trial) OR (random* AND controlled AND study*) OR (random* AND controlled AND trial*) OR (random OR randomly AND (allocation OR allocate*))	1,482,813
15	#13 OR #14	9,810,669
16	#12 AND #15	**4,652**
**Search strategy executed on 10th March 2015**		

Table 23.
Search strategy in Cochrane Library database – DBS effectiveness

Search	Search terms	Results
1	Parkinson's disease	3482
2	MeSH descriptor: [Parkinson Disease] explode all trees	2314
3	Parkinson*	5098
4	parkinsonism or (parkinsonian syndrome)	1084

Search	Search terms	Results
5	paralysis agitans	4
6	#2 or #3 or #4 or #5	5098
7	MeSH descriptor: [Deep Brain Stimulation] explode all trees	199
8	DBS	286
9	globus pallidus interna stimulation	17
10	subthalamic nucleus stimulation	190
11	#7 or #8 or #9 or #10	435
12	#7 and #11	247
**Search strategy executed on 10th March 2015**		

A.2.2. Study selection

All studies identified in the search were initially assessed based on title and abstracts applying predefined selection criteria listed below (Table 24). In a second step, publications initially included based on title and abstract screening, were assessed based on the full article. Papers not meeting the inclusion criteria were excluded from further evaluation.

In the table below selection criteria defined according to PICO scheme are shown.

Table 24.
Selection criteria for clinical studies on DBS effectiveness in Parkinson Disease

Criterion	Inclusion	Exclusion
**Population**	• Adult patients with Parkinson's disease	• Other
**Intervention**	• Deep brain stimulation	• Other
**Comparators**	• Not relevant - any comparator	–
**Outcomes**	• Efficacy:<br>• *UPDRS score,*<br>• *Hoehn and Yahr scale,*<br>• *'off" state time,*<br>• *Dyskinesia,*<br>• *LEDD,*<br>• *QoL measured by PDQ-39, PDQ-8, EQ-5D,*<br>• Safety, mortality.	• Lack of interesting outcomes
**Study design**	• RCT,<br>• Observational studies,<br>• Registries,<br>• Case series.	• Cross-over studies, if no results were presented before patients cross,<br>• Case series or studies with < 20 patients.

Criterion	Inclusion	Exclusion
**Other**	• Primary studies,<br>• Publications available in full-text,<br>• Publications in English.	• Reviews, systematic reviews, meta-analysis,<br>• Comments, editorials, letters,<br>• Conference abstracts (unless concern results of primary study independently identified in search),<br>• Data not allowing to calculate change per given time period.
**Publication date**	• Not relevant (up to date of the search - 10th March 2015)	–

For the purposes of economic analysis, additional selection criteria were taken into account, and only studies meeting the following criteria were included:

- Studies reporting UPDRS measured ON-meds,
- Studies with results presented as mean (not median),
- Studies reporting UPDRS scores at least at two follow-up time-points with first of them at least 2 year after treatment initiation.

A brief summary of all 3 searches and results of studies selection is presented below (Table 25, Table 26). The sequential phases of the review process are also illustrated in the flow diagram (Figure 2).

The list of studies included in final analysis together with data extracted from the studies are presented in section A.2.3.

Table 25.
Summary of systematic search for DBS effectiveness studies

Search date	Database	No. of abstracts returned	Initial selection		Final no. of included studies
			No. included in full text analysis	No. of included studies	
10th March 2015	Pubmed, Embase, Cochrane	7,089	751	190 papers (Table 26)	19 papers (Table 27)

Table 26.
Studies initially included in the analysis and final selection – long-term DBS effectiveness

Study	Initial selection	Final selection	Data included in analysis			
			UPDRS I	UPDRS II	UPDRS III	UPDRS IV
**Abboud 2015 [52]**	Qol	OUT (no UPDRS data)	-	-	-	-
**Accolla 2007 [53]**	UPDRS, H&Y, off/dysk, LEDD	OUT (no UPDRS data in two appropriate time points)	-	-	-	-
**Aviles-Olmos 2014 [54]**	UPDRS, off/dysk, LEDD, Qol, safety	IN	-	✓	✓	-
**Aybek 2007 [55]**	UPDRS, LEDD	IN (with Wider 2008)	-	-	✓	-

Study	Initial selection	Final selection	Data included in analysis			
			UPDRS I	UPDRS II	UPDRS III	UPDRS IV
**Baba 2012 [56]**	UPDRS, off/dysk, LEDD, safety	OUT (no UPDRS data in two appropriate time points)	-	-	-	-
**Baizabal Carvallo 2012 [57]**	Mortality	OUT (no UPDRS data)	-	-	–	-
**Bang 2014 [58]**	LEDD, safety	OUT (no UPDRS data)	-	-	-	-
**Bannier 2009 [59]**	UPDRS, H&Y, off/dysk, LEDD	OUT (no UPDRS data in two appropriate time points)	-	-	-	-
**Barichella 2003 [60]**	UPDRS, off/dysk, LEDD, safety	OUT (no UPDRS data in two appropriate time points)	-	-	-	-
**Boviatsis 2010 [61]**	Safety	OUT (no UPDRS data)	-	-	–	-
**Burdick 2010 [62]**	Safety	OUT (no UPDRS data)	-	-	–	-
**Capecci 2005 [63]**	UPDRS, off/dysk, LEDD, safety	OUT (no UPDRS data in two appropriate time points)	-	-	-	-
**Castelli 2010 [64]**	UPDRS, LEDD,	OUT (no UPDRS data in two appropriate time points)	-	-	-	-
**Castrioto 2013 [65]**	UPDRS, off/dysk, LEDD	OUT (no UPDRS data in two appropriate time points / no on med / on stim data)	-	-	-	-
**Castrioto 2015 [66]**	UPDRS, LEDD,	OUT (UPDRS III only baseline data)	-	-	-	-
**Chandran 2014 [67]**	UPDRS, H&Y, off/dysk, LEDD	OUT (no UPDRS data in two appropriate time points)	-	-	-	-
**Chiou 2015 [68]**	UPDRS, LEDD, safety	OUT (no UPDRS data in two appropriate time points or only baseline values)	-	-	-	-
**Cilia 2007 [69]**	UPDRS, LEDD	OUT (no UPDRS data in two appropriate time points)	-	-	-	-
**Cilia 2009 [70]**	UPDRS, LEDD	OUT (no UPDRS data in two appropriate time points)	-	-	-	-
**Cohen 2007 [71]**	LEDD	OUT (no UPDRS data)	-	-	-	-
**Cury 2014 [72]**	UPDRS, H&Y	OUT (no UPDRS data in two appropriate time points / no on med / on stim data)	-	-	-	-
**Daniele 2003 [73]**	UPDRS, LEDD, safety	OUT (no UPDRS data in two appropriate time points)	-	-	-	-
**Daniels 2011 [74]**	UPDRS, off/dysk, LEDD, Qol	OUT (no UPDRS data in two appropriate time points)	-	-	-	-
**Davis 2006 [75]**	UPDRS	OUT (no UPDRS data in two appropriate time points)	-	-	-	-
**Deep-brain stimulation ...2001 [76]**	UPDRS, off/dysk, LEDD, safety	OUT (no UPDRS data in two appropriate time points)	-	-	-	-
**DeLong 2014 [77]**	Safety	OUT (no UPDRS data)	-	-	–	-

Study	Initial selection	Final selection	Data included in analysis			
			UPDRS I	UPDRS II	UPDRS III	UPDRS IV
**Derost 2007 [78]**	UPDRS, H&Y, off/dysk, LEDD, Qol, safety	OUT (no UPDRS data in two appropriate time points or data unclear)	-	-	-	-
**Derrey 2008 [79]**	UPDRS, H&Y, off/dysk, LEDD, safety	OUT (no UPDRS data in two appropriate time points / no on med / on stim data)	-	-	-	-
**Derrey 2010 [80]**	UPDRS, off/dysk, safety	OUT (no UPDRS data in two appropriate time points)	-	-	-	-
**Deuschl 2006 [81]**	UPDRS, LEDD, Qol, safety	OUT (no UPDRS data in two appropriate time points)	-	-	-	-
**Doshi 2003 [82]**	H&Y, safety	OUT (no UPDRS data)	-	-	-	-
**Drapier 2005 [83]**	UPDRS, Qol, safety	OUT (no UPDRS data in two appropriate time points / no on med / on stim data)	-	-	-	-
**Erola 2005 [84]**	Qol	OUT (no UPDRS data)	-	-	-	-
**Erola 2006 [85]**	UPDRS, H&Y, off/dysk, LEDD, safety	OUT (no UPDRS data in two appropriate time points)	-	-	-	-
**Esselink 2004 [86]**	safety	OUT (no UPDRS data)	-	-	-	-
**Esselink 2009 [87]**	UPDRS, safety	OUT (only medians)	-	-	-	-
**Eusebio 2013 [88]**	UPDRS, H&Y, off/dysk, LEDD	OUT (no UPDRS data in two appropriate time points or only baseline UPDRS value)	-	-	-	-
**Evidente 2011 [89]**	UPDRS, LEDD	OUT (no UPDRS data in two appropriate time points / no on med / on stim data)	-	-	-	-
**Falowski 2012 [90]**	Safety	OUT (no UPDRS data)	-	-	–	-
**Fasano 2010 [91]**	UPDRS, LEDD, safety	IN	-	-	✓	-
**Fenoy 2014 [92]**	Safety	OUT (no UPDRS data)	-	-	–	-
**Ferraye 2008 [93]**	UPDRS, LEDD, safety	OUT (no UPDRS data in two appropriate time points)	-	-	-	-
**Ferraye 2013 [94]**	UPDRS, LEDD	OUT (no UPDRS data in two appropriate time points)	-	-	-	-
**Figueiras-Mendez 2002 [95]**	UPDRS, safety	OUT (no UPDRS data in two appropriate time points)	-	-	-	-
**Fluchere 2014 [96]**	UPDRS, H&Y, off/dysk, LEDD, safety	OUT (no UPDRS data in two appropriate time points)	-	-	-	-
**Follett 2010 [97]**	UPDRS, LEDD, Qol, safety	OUT (the same group of patients in Weaver 2012)	-	-	-	-
**Ford 2004 [98]**	UPDRS, H&Y, off/dysk, LEDD, safety	OUT (no UPDRS data in two appropriate time points)	-	-	-	-
**Foubert-Samier 2012 [99]**	UPDRS, LEDD	OUT (no UPDRS data in two appropriate time points / no on med / on stim data)	-	-	-	-

Study	Initial selection	Final selection	Data included in analysis			
			UPDRS I	UPDRS II	UPDRS III	UPDRS IV
**Fraix 2006 [100]**	UPDRS, off/dysk, LEDD, safety	OUT (no UPDRS data in two appropriate time points)	-	-	-	-
**Funkiewiez 2006 [101]**	UPDRS, LEDD	OUT (no UPDRS data in two appropriate time points)	-	-	-	-
**Gan 2007 [102]**	UPDRS, safety	IN (with Gervais-Bernard 2009)	✓	✓	✓	✓
**George 2012 [103]**	H&Y, LEDD	OUT (no UPDRS data)	-	-	-	-
**Gervais-Bernard 2009 [104]**	UPDRS, LEDD, safety	IN (with Gan 2007)	✓	✓	✓	✓
**Gomez-Esteban 2008 [105]**	UPDRS, Qol	OUT (no UPDRS data in two appropriate time points)	-	-	-	-
**Guehl 2006 [106]**	UPDRS, off/dysk, LEDD, safety	OUT (no UPDRS data in two appropriate time points)	-	-	-	-
**Guo 2013 [107]**	UPDRS	OUT (no UPDRS data in two appropriate time points / no on med / on stim data)	-	-	-	-
**Hamasaki 2010 [108]**	UPDRS, LEDD, safety	OUT (no UPDRS data in two appropriate time points)	-	-	-	-
**Hamel 2003 [109]**	UPDRS	OUT (no UPDRS data in two appropriate time points)	-	-	-	-
**Hariz 2008 [110]**	Safety	OUT (no UPDRS data)	-	-	–	-
**Harries 2012 [111]**	UPDRS, off/dysk, safety	OUT (no UPDRS data in two appropriate time points)	-	-	-	-
**Herzog 2003 [112]**	UPDRS, H&Y, off/dysk, safety	OUT (no UPDRS data in two appropriate time points or baseline data only)	-	-	-	-
**Herzog 2009 [113]**	UPDRS	OUT (no UPDRS data in two appropriate time points / no on med / on stim data)	-	-	-	-
**Houeto 2000 [114]**	UPDRS, H&Y, off/dysk, LEDD, safety	OUT (no UPDRS data in two appropriate time points)	-	-	-	-
**Houeto 2006 [115]**	UPDRS, LEDD, Qol, safety	OUT (no UPDRS data in two appropriate time points or only baseline data)	-	-	-	-
**Hung 2013 [116]**	UPDRS, H&Y, LEDD, safety	IN (UPDRS I, IV) OUT (II, III no on med / on stim data)	-	-	-	-
**Jaggi 2004 [117]**	UPDRS, H&Y, off/dysk, LEDD, safety	OUT (no UPDRS data in two appropriate time points)	-	-	-	-
**Janssen 2014 [118]**	UPDRS, LEDD	IN	✓	✓	✓	✓
**Jiang 2013 [119]**	UPDRS, LEDD	OUT (no UPDRS data in two appropriate time points / no on med / on stim data)	-	-	-	-
**Kalteis 2006 [120]**	UPDRS, LEDD, safety	OUT (no UPDRS data in two appropriate time points)	-	-	-	-

Study	Initial selection	Final selection	Data included in analysis			
			UPDRS I	UPDRS II	UPDRS III	UPDRS IV
**Kenney 2007 [121]**	Safety	OUT (no UPDRS data)	-	-	–	-
**Kim 2012 [122]**	UPDRS, H&Y, LEDD,	OUT (no UPDRS data in two appropriate time points)	-	-	-	-
**Kim 2013 [123]**	UPDRS, H&Y, LEDD	IN	✓	✓	✓	✓
**Kim 2015 [124]**	UPDRS, H&Y, LEDD, safety	OUT (data not clear, UPDRS values outside scale)	-	-	-	-
**Kishore 2010 [125]**	UPDRS, H&Y, off/dysk, LEDD, safety	IN	✓	✓	✓	–
**Kleiner-Fisman 2003 [126]**	UPDRS, LEDD, safety	OUT (median values)	-	-	-	-
**Koike 2008 [127]**	UPDRS, LEDD	OUT (no UPDRS data in two appropriate time points)	-	-	-	-
**Krack 2003 [128]**	UPDRS, off/dysk, LEDD, safety	IN	-	✓	✓	-
**Krause 2004 [129]**	UPDRS, H&Y, off/dysk, LEDD	OUT (no UPDRS data in two appropriate time points)	-	-	-	-
**Kumar 2000 [130]**	UPDRS, off/dysk, LEDD, safety	OUT (no UPDRS data in two appropriate time points)	-	-	-	-
**Lagrange 2002 [131]**	UPDRS, off/dysk, LEDD	OUT (no UPDRS data in two appropriate time points)	-	-	-	-
**Langner-Lemercier 2015 [132]**	UPDRS, H&Y, LEDD, Qol	OUT (no UPDRS data in two appropriate time points)	-	-	-	-
**Lee 2008 [133]**	UPDRS, H&Y, LEDD, safety	OUT (no UPDRS data in two appropriate time points)	-	-	-	-
**Lefaucheur 2008 [134]**	UPDRS, H&Y, off/dysk, LEDD, safety	OUT (no UPDRS data in two appropriate time points)	-	-	-	-
**Lewis 2014 [135]**	UPDRS, LEDD, Qol, safety	OUT (no UPDRS data in two appropriate time points / no on med / on stim data)	-	-	-	-
**Lewis 2015 [136]**	UPDRS, LEDD, Qol, safety	OUT (no UPDRS data in two appropriate time points / no on med / on stim data)	-	-	-	-
**Lhommee 2012 [137]**	UPDRS, off/dysk, LEDD	OUT (no UPDRS data in two appropriate time points)	-	-	-	-
**Li 2013 [138]**	UPDRS, LEDD, safety	IN	-	✓	-	-
**Liang 2006 [139]**	UPDRS, off/dysk, safety	OUT (no UPDRS data in two appropriate time points)	-	-	-	-
**Liang 2012 [140]**	UPDRS, H&Y, off/dysk, safety	OUT (no UPDRS data in two appropriate time points / no on med / on stim data)	-	-	-	-
**Lilleeng 2014 [141]**	Safety, mortality	OUT (no UPDRS data)	-	-	–	-

Study	Initial selection	Final selection	Data included in analysis			
			UPDRS I	UPDRS II	UPDRS III	UPDRS IV
**Limousin 1998 [142]**	UPDRS, off/dysk, LEDD, safety	OUT (no UPDRS data in two appropriate time points)	-	-	-	-
**Liu 2013 [143]**	UPDRS, LEDD, off/dysk.	OUT (excluded by clinicians)	-	-	-	-
**Locke 2011 [144]**	UPDRS, H&Y	OUT (no UPDRS data in two appropriate time points / no on med / on stim data)	-	-	-	-
**Lokkegaard 2007 [145]**	LEDD	OUT (no UPDRS data)	-	-	-	-
**Lyons 2004 [146]**	Safety	OUT (no UPDRS data)	-	-	–	-
**Lyons 2006 [147]**	UPDRS, off/dysk, LEDD	OUT (no UPDRS data in two appropriate time points)	-	-	-	-
**Merola 2012 [148]**	UPDRS, LEDD,	OUT (no UPDRS data in two appropriate time points)	-	-	-	-
**Merola 2014 [149]**	UPDRS, H&Y, LEDD	IN (UPDRS IV) OUT (UPDRS II, III not clear)	-	-	-	-
**Minguez-Castellanos 2005 [150]**	UPDRS, H&Y, off/dysk, LEDD, safety	OUT (no UPDRS data in two appropriate time points)	-	-	-	-
**Monteiro 2014 [151]**	UPDRS, LEDD, safety	OUT (no UPDRS data in two appropriate time points)	-	-	-	-
**Montel 2009 [152]**	UPDRS, H&Y, LEDD, Qol	OUT (no UPDRS data in two appropriate time points)	-	-	-	-
**Nazzaro 2011 [153]**	UPDRS, LEDD, Qol, safety	OUT (no UPDRS data in two appropriate time points)	-	-	-	-
**Ngoga 2014 [154]**	Mortality	OUT (no UPDRS data)	-	-	–	-
**Nilsson 2005 [155]**	LEDD	OUT (no UPDRS data)	-	-	-	-
**Nunta-aree 2010 [156]**	UPDRS, off/dysk, LEDD, safety	OUT (no UPDRS data in two appropriate time points or unclear results)	-	-	-	-
**Odekerken 2013 [157]**	UPDRS, off/dysk, LEDD, safety	OUT (no UPDRS data in two appropriate time points)	-	-	-	-
**Ogura 2004 [158]**	H&Y, off/dysk	OUT (no UPDRS data)	-	-	-	-
**Okun 2012 [159]**	UPDRS, H&Y, off/dysk, LEDD, Qol, safety	OUT (no UPDRS data in two appropriate time points)	-	-	-	-
**Okun 2014 [160]**	UPDRS, LEDD	OUT (no UPDRS data in two appropriate time points)	-	-	-	-
**Ondo 2006 [161]**	UPDRS, LEDD, safety	OUT (no UPDRS data in two appropriate time points / no on med / on stim data)	-	-	-	-
**Ory-Magne 2007 [162]**	UPDRS, off/dysk, LEDD, Qol, safety	OUT (no UPDRS data in two appropriate time points / no on med / on stim data)	-	-	-	-

Study	Initial selection	Final selection	Data included in analysis			
			UPDRS I	UPDRS II	UPDRS III	UPDRS IV
**Ostergaard 2002 [163]**	UPDRS, H&Y, off/dysk, LEDD, safety	OUT (no UPDRS data in two appropriate time points)	-	-	-	-
**Ostergaard 2006 [164]**	UPDRS, H&Y, off/dysk, LEDD, safety	OUT (no UPDRS data in two appropriate time points)	-	-	-	-
**Otaka 2010 [165]**	UPDRS, LEDD	OUT (no UPDRS data in two appropriate time points)	-	-	-	-
**Oyama 2012 [166]**	UPDRS, off/dysk, LEDD	OUT (no UPDRS data in two appropriate time points / no on med / on stim data)	-	-	-	-
**Paek 2008 [167]**	UPDRS, H&Y, off/dysk, LEDD, safety	OUT (no UPDRS data in two appropriate time points)	-	-	-	-
**Paek 2011 [167]**	UPDRS, H&Y, off/dysk, LEDD, safety	OUT (no UPDRS data in two appropriate time points)	-	-	-	-
**Paek 2011 [168]**	UPDRS, H&Y, off/dysk, LEDD	OUT (no UPDRS data in two appropriate time points)	-	-	-	-
**Paek 2013 [169]**	UPDRS, H&Y, off/dysk, LEDD, safety	IN	-	-	✓	-
**Paluzzi 2006 [170]**	Safety	OUT (no UPDRS data)	-	-	–	-
**Patel 2015 [171]**	Safety	OUT (no UPDRS data)	-	-	–	-
**Pellaprat 2014 [172]**	UPDRS, LEDD, Qol, safety	OUT (no UPDRS data in two appropriate time points / no on med / on stim data)	-	-	-	-
**Perriol 2006 [173]**	UPDRS, H&Y, LEDD	OUT (no UPDRS data in two appropriate time points / no on med / on stim data)	-	-	-	-
**Piboolnurak 2007 [174]**	UPDRS, off/dysk, LEDD, safety	IN	-	✓	✓	-
**Portman 2006 [175]**	UPDRS, off/dysk, LEDD, safety	OUT (no UPDRS data in two appropriate time points)	-	-	-	-
**Price 2011 [176]**	UPDRS	OUT (no UPDRS data in two appropriate time points / no on med / on stim data)	-	-	-	-
**Reese 2012 [177]**	UPDRS, H&Y	OUT (no on med / on stim data)	-	-	-	-
**Rizzone 2014 [178]**	UPDRS, off/dysk, LEDD, safety	OUT (no UPDRS data in two appropriate time points)	-	-	-	-
**Rocha 2014 [179]**	Mortality	OUT (no UPDRS data)	-	-	–	-
**Rodriguez-Oroz 2005 [180]**	UPDRS, off/dysk, LEDD, safety	OUT (no UPDRS data in two appropriate time points)	-	-	-	-

Study	Initial selection	Final selection	Data included in analysis			
			UPDRS I	UPDRS II	UPDRS III	UPDRS IV
**Romito 2002 [181]**	UPDRS, H&Y, off/dysk, LEDD, safety	OUT (no UPDRS data in two appropriate time points / no on med / on stim data)	-	-	-	-
**Romito 2003 [182]**	UPDRS, H&Y, off/dysk, LEDD, safety	OUT (unclear measurement condition)	-	-	-	-
**Romito 2009 [183]**	UPDRS, off/dysk, LEDD, safety	IN (only UPDRS II) OUT (UPDRS III no on med / on stim data)	-	✓	–	-
**Schlaier 2014 [184]**	UPDRS, LEDD, safety	OUT (no UPDRS data in two appropriate time points)	-	-	-	-
**Schupbach 2005 [185]**	UPDRS, off/dysk, LEDD, safety	IN	✓	✓	✓	✓
**Schupbach 2007 [186]**	UPDRS, LEDD, safety	OUT (no UPDRS data in two appropriate time points)	-	-	-	-
**Seijo 2007 [187]**	Safety	OUT (no UPDRS data)	-	-	–	-
**Seijo 2014 [188]**	Safety	OUT (no UPDRS data)	-	-	–	-
**Simonin 2009 [189]**	UPDRS, off/dysk	OUT (no UPDRS data in two appropriate time points)	-	-	-	-
**Smeding 2006 [190]**	UPDRS, H&Y, safety	OUT (no UPDRS data in two appropriate time points / no on med / on stim data)	-	-	-	-
**Smeding 2011 [191]**	UPDRS, H&Y, safety	OUT (no UPDRS data in two appropriate time points / no on med / on stim data)	-	-	-	-
**Soulas 2011 [192]**	UPDRS, Qol, safety	OUT (no UPDRS data in two appropriate time points / no on med / on stim data)	-	-	-	-
**Soulas 2012 [193]**	UPDRS, LEDD, Qol,	OUT (no UPDRS data in two appropriate time points / no on med / on stim data)	-	-	-	-
**Sung 2013 [194]**	UPDRS	OUT (no UPDRS data in two appropriate time points)	-	-	-	-
**Tagliati 2010 [195]**	UPDRS, LEDD, safety	OUT (no UPDRS data in two appropriate time points / no on med / on stim data)	-	-	-	-
**Tai 2010 [196]**	UPDRS, H&Y, LEDD, safety	OUT (no UPDRS data in two appropriate time points)	-	-	-	-
**Tanei 2009 [197]**	UPDRS, H&Y, LEDD, safety	OUT (no UPDRS data in two appropriate time points)	-	-	-	-
**Tang 2015 [198]**	UPDRS, safety	OUT (no UPDRS data in two appropriate time points or only baseline UPDRS value)	-	-	-	-
**Tavella 2002 [199]**	UPDRS, LEDD, safety	OUT (no UPDRS data in two appropriate time points / no on med / on stim data)	-	-	-	-
**Temel 2007 [200]**	UPDRS, LEDD, safety	OUT (no UPDRS data in two appropriate time points)	-	-	-	-

Study	Initial selection	Final selection	Data included in analysis			
			UPDRS I	UPDRS II	UPDRS III	UPDRS IV
**Tir 2007 [201]**	UPDRS, off/dysk, LEDD, safety	OUT (no UPDRS data in two appropriate time points)	-	-	-	-
**Toft 2011 [202]**	UPDRS, LEDD, safety	IN	-	-	✓	-
**Troche 2014 [203]**	UPDRS, LEDD	OUT (no UPDRS data in two appropriate time points)	-	-	-	-
**Troster 2003 [204]**	UPDRS, LEDD, Qol	OUT (no UPDRS data in two appropriate time points)	-	-	-	-
**Tsai 2009 [205]**	UPDRS, H&Y, LEDD, safety	OUT (no on med / on stim data or unclear data)	-	-	-	-
**Tsai 2012 [206]**	UPDRS, H&Y, LEDD, safety	OUT only baseline data or unclear measurement condition)	-	-	-	-
**Tykocki 2013 [207]**	UPDRS, LEDD, Qol	OUT (UPDRS III only baseline)	-	-	-	-
**Tykocki 2013 [208]**	UPDRS, LEDD, Qol	OUT (no UPDRS data in two appropriate time points)	-	-	-	-
**Umemura 2011 [209]**	Safety	OUT (no UPDRS data)	-	-	–	-
**Vercruysse 2014 [210]**	UPDRS, H&Y, off/dysk, LEDD, Qol	OUT (no UPDRS data in two appropriate time points or unclear data)	-	-	-	-
**Vergani 2010 [211]**	Safety	OUT (no UPDRS data)	-	-	–	-
**Vesper 2007 [212]**	UPDRS, H&Y, LEDD, safety	OUT (no UPDRS data in two appropriate time points / on med / on stim data)	-	-	-	-
**Vingerhoets 1999 [213]**	H&Y, LEDD	OUT (no UPDRS data)	-	-	-	-
**Vingerhoets 2002 [214]**	UPDRS, off/dysk, LEDD, safety	OUT (unclear measurement condition)	-	-	-	-
**Visser-Vandewalle 2003 [215]**	UPDRS, off/dysk, LEDD, safety	OUT (no UPDRS data in two appropriate time points)	-	-	-	-
**Visser-Vandewalle 2005 [216]**	UPDRS, off/dysk, LEDD, safety	OUT (no UPDRS data in two appropriate time points)	-	-	-	-
**Voges 2006 [217]**	Safety	OUT (no UPDRS data)	-	-	–	-
**Volkmann 2009 [218]**	UPDRS, off/dysk, safety	OUT (no UPDRS data in two appropriate time points)	-	-	-	-
**Walker 2009 [219]**	UPDRS, off/dysk, LEDD, Qol, safety	OUT (no UPDRS data in two appropriate time points)	-	-	-	-
**Wang 2009 [220]**	UPDRS	OUT (unclear data)	-	-	-	-
**Weaver 2009 [221]**	UPDRS, H&Y, off/dysk, LEDD, Qol, safety	OUT (no UPDRS data in two appropriate time points)	-	-	-	-

Study	Initial selection	Final selection	Data included in analysis			
			UPDRS I	UPDRS II	UPDRS III	UPDRS IV
**Weaver 2012 [222]**	UPDRS, off/dysk, LEDD, Qol, safety	IN (NCT00056563 / NCT01076452 study)	✓	✓	✓	✓
**Welter 2014 [223]**	UPDRS, LEDD, safety	OUT (no UPDRS data in two appropriate time points)	-	-	-	-
**Wenzelburger 2003 [224]**	UPDRS, off/dysk, LEDD	OUT (no UPDRS data in two appropriate time points)	-	-	-	-
**Wider 2006 [225]**	UPDRS, LEDD	OUT (no UPDRS data in two appropriate time points)	-	-	-	-
**Wider 2008 [226]**	UPDRS, off/dysk, LEDD, safety	IN (with Aybek 2007)	✓	✓	✓	-
**Williams 2010 [227]**	UPDRS, off/dysk, LEDD, Qol, safety	OUT (no UPDRS data in two appropriate time points)	-	-	-	-
**Winkler 2013 [228]**	LEDD	OUT (no UPDRS data)	-	-	-	-
**Witjas 2007 [229]**	UPDRS, H&Y, off/dysk, LEDD	OUT (no UPDRS data in two appropriate time points)	-	-	-	-
**Witt 2011 [230]**	UPDRS, off/dysk, LEDD, Qol,	OUT (no UPDRS data in two appropriate time points)	-	-	-	-
**Witt 2013 [231]**	UPDRS, H&Y, LEDD	OUT (only baseline data for UPDRS III)	-	-	-	-
**Yamada 2006 [232]**	UPDRS, LEDD	OUT (no UPDRS data in two appropriate time points)	-	-	-	-
**Yamada 2007 [233]**	UPDRS, off/dysk, LEDD,	OUT (no UPDRS data in two appropriate time points)	-	-	-	-
**Yamada 2008 [234]**	UPDRS, LEDD, safety	OUT (no UPDRS data in two appropriate time points)	-	-	-	-
**Yamada 2009 [235]**	UPDRS, LEDD, safety	OUT (no UPDRS data in two appropriate time points)	-	-	-	-
**Yamada 2010 [236]**	UPDRS, LEDD	OUT (no UPDRS data in two appropriate time points)	-	-	-	-
**Zahodne 2009 [237]**	UPDRS, LEDD, Qol	OUT (no UPDRS data in two appropriate time points)	-	-	-	-
**Zangaglia 2009 [238]**	UPDRS, H&Y, LEDD	OUT (no UPDRS data in two appropriate time points)	-	-	-	-
**Zibetti 2007 [239]**	UPDRS, off/dysk, safety	OUT (no UPDRS data in two appropriate time points / no on med / on stim data)	-	-	-	-
**Zibetti 2009 [240]**	UPDRS, LEDD	OUT (no UPDRS data in two appropriate time points / no on med / on stim data)	-	-	-	-

Off – off time, dysk – dyskinesia, LEDD – levodopa equivalent daily dose

Figure 2.
Overview of studies selection: PRISMA flowchart – long-term DBS effectiveness

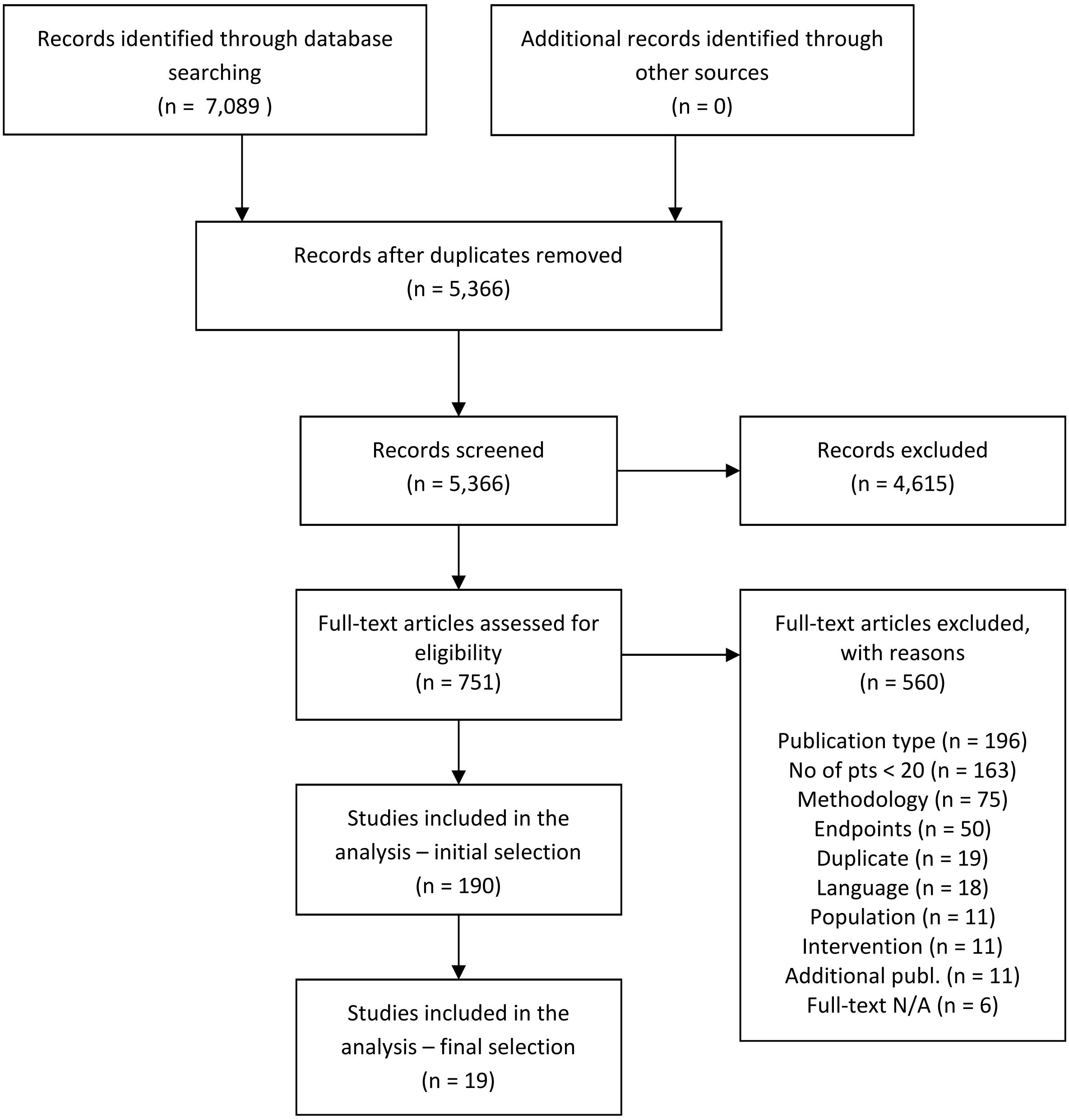

A.2.3. Data extraction

Studies included in the analysis for particular UPDRS scores are listed in Table 27. Brief description of the studies is provided in Table 28.

Table 27
Long-term DBS effectiveness studies included in the analysis

Parameter	UPDRS I	UPDRS II	UPDRS III	UPDRS IV
**Publications included**	Weaver 2012<br>Gan 2007 / Gervais-Bernard 2009<br>Hung 2013<br>Janssen 2014<br>Kim 2013<br>Kishore 2010<br>Schupbach 2005<br>Wider 2008	Aviles-Olmos 2014<br>Weaver 2012<br>Gan 2007 / Gervais-Bernard 2009<br>Janssen 2014<br>Kim 2013<br>Kishore 2010<br>Krack 2003<br>Li 2013<br>Piboolnurak 2007<br>Romito 2009<br>Schupbach 2005<br>Wider 2008	Aviles-Olmos 2014<br>Aybek 2007 / Wider 2008<br>Fasano 2010<br>Weaver 2012<br>Gan 2007 / Gervais-Bernard 2009<br>Janssen 2014<br>Kim 2013<br>Kishore 2010<br>Krack 2003<br>Paek 2013<br>Piboolnurak 2007<br>Schupbach 2005<br>Toft 2011	Weaver 2012<br>Gan 2007 / Gervais-Bernard 2009<br>Hung 2013<br>Janssen 2014<br>Kim 2013<br>Merola 2014<br>Schupbach 2005
**Total number of publications/studies**	**9/8**	**13/12**	**15/13**	**8/7**

Table 28
Study characteristic – DBS long-term effectiveness

Study	Study design	Follow-up	No of patients	Baseline patients characteristics			
				**Age [mean (SD)]**	**Duration of PD [years (SD)]**	**Hoehn & Yahr (SD)**	**LEDD [mg (SD)]**
**Aviles-Olmos 2014**	**Design:** nonrandomized, prospective<br>**Aim:** To report the long term outcome in a cohort of individuals who underwent STN DBS<br>**Population:** United Kingdom	8 years	41	56.2 (8.4)	12.9 (5.8)	Not reported	1 471 (515)
**Aybek 2007**	**Design:** nonrandomized, prospective<br>**Aim:** to evaluate the long-term cognitive profile and the incidence of dementia in a cohort of PD patients treated by STN-DBS<br>**Population:** Switzerland	3 years	57	63.8 (8)	15.7 (5)	Not reported	1 097.5 (523.0)
**Wider 2008**	**Design:** nonrandomized, prospective, single center<br>**Aim:** To describe the long-term outcome in advanced PD patients treated with STN-DBS.<br>**Population:** Switzerland	5 years	50	64.9 (7.6)	14.4 (4.9)	Not reported	1128 (493)
**Fasano 2010**	**Design:** nonrandomized, prospective, single center<br>**Aim:** To provides long-term assessment of patients who underwent STN-DBS<br>**Population:** Italy	8 years<br>Mean: 96 (3,1) months	20	56.9 (7.2)	13.7 (4.8)	≥3	1 418.2 (782.8)

Study	Study design	Follow-up	No of patients	Baseline patients characteristics			
				Age [mean (SD)]	Duration of PD [years (SD)]	Hoehn & Yahr (SD)	LEDD [mg (SD)]
**Weaver 2012**	**Design:** RCT, prospective, multicenter<br>**Aim:** to compare long-term outcomes of DBS of GPi and STN for PD patients<br>**Population:** USA	3 years	159	60.53 (8.57)	Not reported	OFF: 3.3 (0.8)	1323.18 (557.05)
**Gan 2007**	**Design:** RCT, prospective<br>**Aim:** To assess the long-term efficacy and safety of STN-DBS in patients with advanced PD.<br>**Population:** France	3 years	36	55.4 (8.3)	12.5 (4.0)	Not reported	1 228.3 (648.9)
**Gervais-Bernard 2009**	**Design:** RCT, prospective<br>**Aim:** To assess the long-term efficacy and safety of STN-DBS in patients with advanced PD.<br>**Population:** France	5 years	23	55.1 (7.2)	12.9 (3.2)	Not reported	1 188 (465)
**Hung 2013**	**Design:** nonrandomized, prospective<br>**Aim:** Assessing which target symptoms have long-term effects from STN-DBS.<br>**Population:** Taiwan.	7 years	120	49.5 (11.6)	9.8 (5.1)	3.3 (0.9)	779.1 (389.5)
**Janssen 2014**	**Design:** nonrandomized, observational, prospective, cohort study<br>**Aim:** To provide an analysis of motor and cognitive outcome after STN DBS<br>**Population:** Netherlands	Mean: 89.6 (40.6) months<br>10 years	26	58.0 (6.9)	12.7 (5.1)	Not reported	824 (479)
**Kim 2013**	**Design:** nonrandomized, prospective<br>**Aim:** To analyze long-term follow-up data of STN DBS cases and to identify the factors related to outcomes<br>**Population:** South Korea	7 years<br>Mean: 57.48 months	52	57.60 (10.58)	10.25 (4.84)	3.07 (0.76)	957.16 (487.10)
**Kishore 2010**	**Design:** nonrandomized, prospective<br>**Aim:** Assessing stability of effects of STN stimulation in PD<br>**Population:** India	5 years	45	55.4 (10.9)	11.1 (5.7)	ON: 2.3 (0.5)<br>OFF: 3.7 (0.9)	669.8 (359.7)
**Krack 2003**	**Design:** nonrandomized, prospective<br>**Aim:** To report long-term outcomes of STN-DBS therapy.<br>**Population:** France	5 years	49	55.0 (7.5)	14.6 (5.0)	Not reported	1 409 (605)
**Li 2013**	**Design:** nonrandomized, prospective<br>**Aim:** To evaluate the outcome of bilateral STN stimulation to PD patients<br>**Population:** China	8 years	31	53.5 (11.7)	7.86	Stage: 2-4	967.8 (381.3)

Study	Study design	Follow-up	No of patients	Baseline patients characteristics			
				Age [mean (SD)]	Duration of PD [years (SD)]	Hoehn & Yahr (SD)	LEDD [mg (SD)]
**Merola 2014**	**Design:** observational, nonrandomized, retrospective<br>**Aim:** To analyze the role of PD-MCI in patients treated with STN-DBS<br>**Population:** Italy	5 years	174	60.31 (6.72)	13.91 (4.75)	ON: 2.62 (0.81)<br>OFF: 3.46 (0.99)	1 063.59 (409.73)
**Paek 2013**	**Design:** nonrandomized, retrospective<br>**Aim:** to compare the long-term clinical outcomes of advanced PD patients following bilateral STN-DBS in terms of the positioning of their electrodes<br>**Population:** South Korea	3 years	41	61.9 (7.9)	13.7 (4.1)	ON: 2.3 (0.7)<br>OFF: 3.1 (1.0)	896.8 (423.7)
**Piboolnurak 2007**	**Design:** nonrandomized, prospective<br>**Aim:** To evaluate changes in the l-dopa response over time in STN-DBS<br>**Population:** Canada	5 years	33	53.4 (8.3)	13.5 (4.7)	Not reported	1 267.2 (521.2)
**Romito 2009**	**Design:** nonrandomized, prospective<br>**Aim:** To reduce and stabilize dopaminergic medication after STN stimulation<br>**Population:** Italy	5 years	20	56.4 (6.9)	14.3 (6.2)	ON: 2.6 (0.8)<br>OFF: 4.4 (0.9)	1 457.6 (785.6)
**Schupbach 2005**	**Design:** nonrandomized, prospective<br>**Aim:** To provides a follow up data of PD patients treated with stimulation of the STN<br>**Population:** France	5 years	37	54.9 (9.1)	15.2 (5.3)	Median: 5 (4-5)	1 468 (811)
**Toft 2011**	**Design:** nonrandomized, retrospective, single center.<br>**Aim:** To report long-term data of STN-DBS treatment efficacy and mortality<br>**Population:** Norway	Mean: 3.3 years<br>5 years	144	60.3 (7.8)	11.0 (4.8)	Not reported	991 (462)

The data extracted from the studies included in the analysis is presented below (Table 29, Table 30, Table 31, Table 32).

Table 29.
DBS long-term effectiveness - UPDRS I – data extraction

Study	Publication(s)	Mean UPDRS I (no of patients)								
		Baseline	1 yrs.	2 yrs.	3 yrs.	4 yrs.	5 yrs.	6 yrs.	7 yrs.	10 yrs.
Gan 2007 / Gervais-Bernard 2009	Gan 2007	1.30 (36)	1.70 (36)	N/D	2.40 (36)	N/D	N/D	N/D	N/D	N/D
	Gervais-Bernard 2009	0.83 (23)	1.48 (23)	N/D	N/D	N/D	2.57 (23)	N/D	N/D	N/D
Hung 2013		4.60 (120)	2.90 (88)	2.90 (60)	N/D	N/D	3.30 (31)	N/D	3.80 (17)	N/D
Kim 2013		3.60 (52)	2.90 (52)	2.90 (52)	3.20 (52)	3.60 (<52)[a]	4.40 (<52)[a]	4.50 (<52)[a]	3.00 (<52)[a]	N/D
Kishore 2010		1.30 (45)	1.30 (45)	N/D	1.20 (36)	N/D	2.10 (29)	N/D	N/D	N/D
Weaver 2012		2.79 (159)	N/D	2.88 (157)	3.23 (159)	N/D	N/D	N/D	N/D	N/D
Schupbach 2005		2.30 (37)	N/D	2.00 (32)	N/D	N/D	3.30 (30)	N/D	N/D	N/D
Janssen 2014		1.80 (26)	1.40 (26)	N/D	N/D	N/D	3.10 (18)	N/D	N/D	4.20 (12)
Wider 2008		1.80 (37)	N/D	2.60 (36)	N/D	N/D	3.70 (37)	N/D	N/D	N/D

a) exact number of patients not reported (for the purposes of the analysis 52 assumed at all time points)

Table 30.
DBS long-term effectiveness - UPDRS II (on med / on stim) – data extraction

Study	Publication(s)	Mean UPDRS II (no of patients)									
		Baseline	1 yrs.	2 yrs.	3 yrs.	4 yrs.	5 yrs.	6 yrs.	7 yrs.	8 yrs.	10 yrs.
Aviles-Olmos 2014		6.20 (41)	7.50 (41)	N/D	N/D	N/D	13.20 (41)	N/D	N/D	15.20 (12)	N/D
Gan 2007 / Gervais-Bernard 2009	Gan 2007	4.50 (36)	8.50 (36)	N/D	10.40 (36)	N/D	N/D	N/D	N/D	N/D	N/D
	Gervais-Bernard 2009	4.70 (23)	7.65 (23)	N/D	N/D	11.35 (23)	N/D	N/D	N/D	N/D	N/D
Krack 2003		7.30 (49)	7.40 (43)	N/D	10.70 (40)	N/D	14.00 (39)	N/D	N/D	N/D	N/D
Kim 2013		14.63 (52)	10.15 (52)	10.30 (52)	10.70 (52)	11.10 (<52)[a]	12.50 (<52)[a]	12.80 (<52)[a]	12.70 (<52)[a]	N/D	N/D

Study	Publication(s)	Mean UPDRS II (no of patients)									
		Baseline	1 yrs.	2 yrs.	3 yrs.	4 yrs.	5 yrs.	6 yrs.	7 yrs.	8 yrs.	10 yrs.
Kishore 2010		9.50 (49)	5.90 (45)	N/D	7.30 (36)	N/D	7.10 (29)	N/D	N/D	N/D	N/D
Li 2013		8.80 (31)	6.40 (31)	N/D	N/D	N/D	10.90 (31)	N/D	N/D	16.70 (29)	N/D
Weaver 2012		18.92 (159)	N/D	14.97 (155)	17.50 (157)	N/D	N/D	N/D	N/D	N/D	N/D
Piboolnurak 2007		11.20 (33)	N/D	N/D	13.20 (33)	N/D	14.60 (17)	N/D	N/D	N/D	N/D
Romito 2009		10.90 (20)	7.50 (20)	N/D	8.90 (20)	N/D	8.60 (20)	N/D	N/D	N/D	N/D
Schupbach 2005		11.50 (37)	N/D	10.00 (32)	N/D	N/D	14.30 (30)	N/D	N/D	N/D	N/D
Janssen 2014		11.30 (26)	8.60 (23)	N/D	N/D	N/D	14.30 (18)	N/D	N/D	N/D	20.40 (12)
Wider 2008		10.00 (37)	N/D	12.80 (36)	N/D	N/D	18.90 (37)	N/D	N/D	N/D	N/D

a) exact number of patients not reported (for the purposes of the analysis 52 assumed at all time points)

Table 31.
DBS long-term effectiveness - UPDRS III (on med / on stim) – data extraction

Study	Publication(s)	Mean UPDRS III (no of patients)									
		Baseline	1 yrs.	2 yrs.	3 yrs.	4 yrs.	5 yrs.	6 yrs.	7 yrs.	8 yrs.	10 yrs.
Aviles-Olmos 2014		14.00 (41)	14.00 (41)	N/D	N/D	N/D	23.90 (41)	N/D	N/D	28.70 (12)	N/D
Fasano 2010		24.50 (20)	21.30 (20)	N/D	22.90 (20)	N/D	21.40 (20)	N/D	N/D	26.90 (20)	N/D
Gan 2007 / Gervais-Bernard 2009	Gan 2007	14.10 (36)	12.50 (36)	N/D	12.50 (36)	N/D	N/D	N/D	N/D	N/D	N/D
	Gervais-Bernard 2009	14.83 (23)	10.34 (23)	N/D	N/D	N/D	13.17 (23)	N/D	N/D	N/D	N/D
Krack 2003		14.30 (49)	11.40 (43)	N/D	15.30 (40)	N/D	21.10 (39)	N/D	N/D	N/D	N/D
Kim 2013		21.70 (52)	17.00 (52)	17.70 (52)	18.50 (52)	18.50 (<52)[a]	22.10 (<52)[a]	21.80 (<52)[a]	22.10 (<52)[a]	N/D	N/D
Kishore 2010		16.20 (45)	13.90 (45)	N/D	14.70 (36)	N/D	16.50 (29)	N/D	N/D	N/D	N/D
Weaver 2012		21.26 (159)	N/D	19.43 (146)	21.96 (143)	N/D	N/D	N/D	N/D	N/D	N/D
Paek 2013		19.20 (41)	15.00 (41)	14.20 (41)	19.50 (41)	N/D	N/D	N/D	N/D	N/D	N/D

Study	Publication(s)	Mean UPDRS III (no of patients)									
		Baseline	1 yrs.	2 yrs.	3 yrs.	4 yrs.	5 yrs.	6 yrs.	7 yrs.	8 yrs.	10 yrs.
Piboolnurak 2007		22.40 (33)	N/D	N/D	23.00 (33)	N/D	24.80 (17)	N/D	N/D	N/D	N/D
Schupbach 2005		17.80 (37)	N/D	10.10 (32)	N/D	N/D	17.90 (30)	N/D	N/D	N/D	N/D
Janssen 2014		21.20 (26)	13.00 (26)	N/D	N/D	N/D	21.70 (18)	N/D	N/D	N/D	28.70 (12)
Toft 2011		13.20 (131)	12.50 (131)	15.40 (110)	18.10 (89)	19.50 (52)	22.40 (32)	N/D	N/D	N/D	N/D
Wider / Aybek study	Aybek 2007	22.70 (43)	N/D	N/D	25.10 (43)	N/D	N/D	N/D	N/D	N/D	N/D
	Wider 2008	24.30 (37)	N/D	27.70 (36)	N/D	N/D	30.60 (37)	N/D	N/D	N/D	N/D

a) exact number of patients not reported (for the purposes of the analysis 52 assumed at all time points)

Table 32.
DBS long-term effectiveness - UPDRS IV – data extraction

Study	Publication(s)	Mean UPDRS IV (no of patients)								
		Baseline	1 yrs.	2 yrs.	3 yrs.	4 yrs.	5 yrs.	6 yrs.	7 yrs.	10 yrs.
Gan 2007 / Gervais-Bernard 2009	Gan 2007	9.00 (36)	1.90 (36)	N/D	3.10 (36)	N/D	N/D	N/D	N/D	N/D
	Gervais-Bernard 2009	8.30 (23)	1.24 (23)	N/D	N/D	N/D	3.26 (23)	N/D	N/D	N/D
Hung 2013		5.50 (120)	1.70 (88)	1.80 (60)	N/D	N/D	1.90 (31)	N/D	1.80 (17)	N/D
Kim 2013		7.63 (52)	4.10 (52)	4.40 (52)	4.60 (52)	4.80 (<52) [a]	6.00 (<52) [a]	6.40 (<52) [a]	5.40 (<52) [a]	N/D
Merola 2014		8.03 (174)	2.20 (174)	N/D	2.72 (174)	N/D	3.32 (174)	N/D	N/D	N/D
Weaver 2012		9.03 (157)	N/D	4.56 (122)	4.21 (124)	N/D	N/D	N/D	N/D	N/D
Schupbach 2005		12.70 (37)	N/D	3.20 (32)	N/D	N/D	5.10 (30)	N/D	N/D	N/D
Janssen 2014		7.20 (26)	1.50 (26)	N/D	N/D	N/D	2.40 (18)	N/D	N/D	2.30 (12)

a) exact number of patients not reported (for the purposes of the analysis 52 assumed at all time points)

A.3. Long-term progression in BMT studies

A.3.1. Search strategy

In order to identify data on long-term progression of UPDRS outcomes (for modelling of "natural disease progression") a systematic search of "BMT" studies was performed in Medline (via PubMed). "BMT" was defined as any medical intervention (except DBS, CSAI, CDLCI), and intervention studies of PD medication, or population-based studies, were included. The search in Medline database was performed on 12th February 2015. The search was performed in the initial phase of the project, when model structure and data included in the analysis were unknown. Therefore, broad inclusion criteria were defined (details in Table 34).

An additional non-systematic search via google and in ISPOR database [241] was done. As this was non-systematic no specific strategy was used.

Table 33.
Search strategy in Medline database – natural disease progression

Search	Search terms	Results
**#6**	#4 AND #5	578
**#5**	longterm OR long-term OR "long term" OR natural OR progress OR progression OR decline OR history OR course OR predict OR prediction OR fall OR advancement OR evolution OR evolve OR transition	3,570,509
**#4**	#1 AND #2 AND #3	1,502
**#3**	UPDRS OR "Unified Parkinson's Disease Rating Scale" or (Hoehn and (Yahr's or Yahr)) OR PDQ OR "Parkinson's Disease Questionnaire" OR EuroQol OR EQ5D OR EQ-5D OR "EQ 5D"	9,424
**#2**	epidemiol* OR cross-section OR cross-sectional OR "cross sectional" OR register OR population-based OR prospective OR retrospective OR registry OR registries OR observational OR longitudinal OR database	2,843,637
**#1**	(Parkinson's disease) OR ("Parkinson Disease"[Mesh]) OR (Parkinson*) OR (parkinsonism OR (parkinsonian syndrome)) OR (paralysis agitans)	96,372
	**Search strategy executed on 12th February 2015**	

A.3.2. Study selection

All studies identified within search process were initially assessed based on title and abstracts. Papers not meeting predefined inclusion criteria as indicated by title and abstracts were excluded.

A summary of primary selection criteria is presented in Table 34.

Table 34.
Selection criteria for studies on Parkinson's Disease progression in BMT treated patients

Criterion	Inclusion	Exclusion
**Population**	• Patients with Parkinson's Disease	• Other disease
**Intervention**	• Any medical intervention (except those listed in exclusion criteria); if specific intervention assessed, study included only if results reported in the period beyond first two years from treatment initiation	• DBS, CSAI, CDLCI
**Data type**	• Disease severity / progression measured as:<br>• *UPDRS,*<br>• *H&Y,*<br>• *QoL change (PDQ-39, EQ-5D)*<br>• *off time, motor complications occurrence, dyskinesias severity, etc.*<br>• Data reported as:<br>• *annual change in parameter value,*<br>• *probability of progression / transition between health states,*<br>• *any data allowing to calculate progression rate within time*	• Lack of data specified in inclusion criteria
**Study design**	• Any type of study	-
**Publication date**	• Not relevant (up to date of search – 12th February 2015)	-

Data from 36 publications were included after full text analysis based on selection criteria. At this stage of the search, references of the retrieved publications were also checked and a non-systematic search was done (Table 36). As a result, 30 additional papers were included.

For the purposes of economic analysis, additional selection was done and only studies reporting data on UPDRS progression were finally included. Additional selection criteria, in order to have data to derive UPDRS progression rates, were:

- UPDRS scores measured ON-meds,
- at least 2-year follow-up (excluding initial period of treatment – if specific intervention assessed, first 2 years of treatment excluded from the analysis – to eliminate short-term effectiveness),
- studies performed in early / recently diagnosed PD patient were excluded (the exception is Reinosso 2014 study [242] – performed in recently diagnosed PD patients, but results reported separately for period 7-9 years from diagnosis).

A brief summary of the search and results of study selection is presented in Table 35 and Table 36.

Table 35.
Summary of systematic search for PD progression in BMT treated patients

Search date	Database	No. of abstracts returned	Initial selection		Final no. of included studies
			No. included in full text analysis	No. of included studies	
12th February 2015	Pubmed	578	75	36	5

Table 36
Summary of systematic search for PD progression in BMT treated patients – Additional search

Data source	No. of considered studies (initial selection)	Final no. of included studies
References of the retrieved publications	24	1
Other (google, ISPOR)	6	0

Figure 3.
Overview of studies selection: PRISMA flowchart – long-term progression in BMT studies

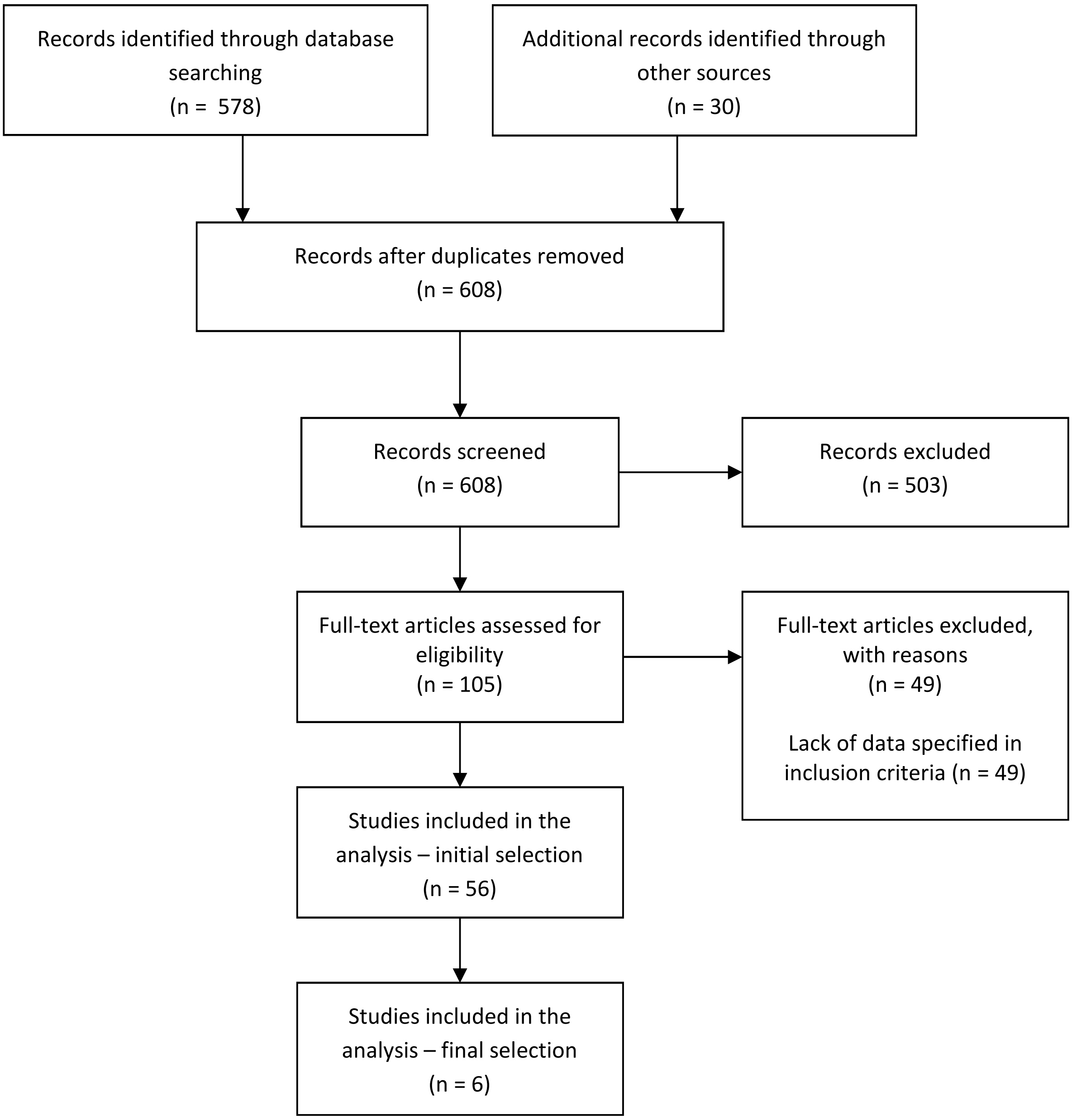

No data for UPDRS IV progression were identified. The studies included in the final analysis for UPDRS I-III are listed in Table 37.

Table 37
Parkinson's Disease progression in BMT treated patients in final analysis

Parameter	Disease progression measured by:			
	UPDRS I	UPDRS II	UPDRS III	UPDRS IV
**Publications included**	Jankovic 2001 [243]	Alves 2005 [244]<br>Jankovic 2001 [243]<br>Le Witt 2013 [245]	Alves 2005 [244]<br>Jankovic 2001 [243]<br>Le Witt 2013 [245]<br>Reinosso 2014 [242]<br>Brooks 2008 [246]<br>Schrag 2007 [247]	-
**Total number of publications / studies**	**1**	**3**	**6**	**0**

The publications included after initial selection, but finally excluded from the analysis are listed in Table 38.

Table 38.
Studies on BMT progression not included in final calculation

Publication	Reason for exclusion
**Antonini 2012 [248]**	population of early PD
**Bloem 2001 [249]**	no data on UPDRS progression
**Chan 2007 [250]**	data on total UPDRS only
**Cheng 2014 [251]**	no data on UPDRS progression
**Chia 1992 [252]**	no data on UPDRS progression
**Di Rocco 1996 [253]**	no data on UPDRS progression
**Duarte 2015 [254]**	not enough data for calculation
**Evans 2011 [255]**	no data on UPDRS progression
**Factor 2001 [256]**	short follow-up
**Foltynie 2004 [257]**	no data on UPDRS progression
**Gago 2009 [258]**	not enough data for calculation
**Gazibara 2014 [259]**	no data on UPDRS progression
**Goetz 1988 [260]**	no data on UPDRS progression
**Guimaraes 2005 [261]**	not enough data for calculation
**Hauser 2006 [262]**	no data on UPDRS progression
**Hauser 2007 [263]**	not enough data for calculation
**Hely 1999 [264]**	no data on UPDRS progression
**Hely 2005 [265]**	no data on UPDRS progression
**Hiorth 2013 [266]**	no data on UPDRS progression
**Hiorth 2014 [267]**	no data on UPDRS progression
**Hoehn 1983 [268]**	no data on UPDRS progression
**Holford 2006 [269]**	data on total UPDRS only
**Janssen 2014 [118]**	DBS study

Publication	Reason for exclusion
**Johnson 2013 [270]**	no data on UPDRS progression
**Johnson 2013 [271]**	no data on UPDRS progression
**Klotsche 2011 [272]**	not enough data for calculation
**Lang 2013 [273]**	modified UPDRS
**Larsen 1999 [274]**	early PD
**Lindholm 2015 [275]**	no data on UPDRS progression
**Liou 2008 [276]**	no data on UPDRS progression
**Lopez 2010 [277]**	population of early PD
**Matinolli 2011 [278]**	no data on UPDRS progression
**Muller 2000 [279]**	no data on UPDRS progression
**Pickering 2007 [280]**	no data on UPDRS progression
**Poewe 2006 [281]**	no data on UPDRS progression
**Rajput 2009 [282]**	no data on UPDRS progression
**Rascol 2000 [283]**	early PD
**Roos 1996 [284]**	no data on UPDRS progression
**Rudzinska 2008 [285]**	no data on UPDRS progression
**Rudzinska 2013 [286]**	no data on UPDRS progression
**Rudzinska 2013 [287]**	no data on UPDRS progression
**Sato 2006 [288]**	no data on UPDRS progression
**Schrag 2000 [289]**	no data on UPDRS progression
**Schrag 2009 [290]**	short follow-up
**Stocchi 2002 [291]**	data on graph. hard to read in details
**Velseboer 2013 [292]**	no data on UPDRS progression
**Vu 2012 [293]**	not enough data for calculation
**Williams-Gray 2013 [294]**	no data on UPDRS progression
**Zhao 2010 [295]**	no data on UPDRS progression
**Zhao 2011 [296]**	no data on UPDRS progression

A.3.3. Data extraction

Brief description of the studies included in the analysis is provided in Table 39.

Table 39
Study characteristic – BMT long-term progression

Study		Study design	Follow-up	No of patients	Baseline patients characteristics		
					Age [mean (SD)]	Duration of PD [years (SD)]	Hoehn & Yahr (SD)
**Alves 2005**		**Design:** nonrandomized, prospective, **Aim:** To investigate risk factors and the rate of progression of motor symptoms and disability in a population-based cohort of patients with PD. **Population:** Norway	8 years	232	73.5 (8.5)	9.1 (5.7)	2.8 (1.1)
**Brooks 2008**		**Design:** retrospective, pooled data from four studies **Aim:** To evaluate the efficacy and safety of long-term levodopa/DDCI and entacapone therapy in patients with PD **Population:** As in four analyzed studies	5 years	649	63.1 (9.4)	9.5 (5.2)	2.4 (0.7)
**Jankovic 2001**		**Design:** prospective, single center **Aim:** To determine the overall rate of functional decline and to assess the progression of different signs of PD **Population:** Texas/USA	6.36 years	297	55.1	6.5	Not reported
**Le Witt 2013**	**SP516**	**Design:** open-label extension of RCT studies, prospective **Aim:** To evaluate the safety, tolerability and efficacy of the dopaminergic agonist, rotigotine, in patients with advanced PD. **Population:** 19 countries (17 in SP 516, and 2 in SP 715	Up to 4 years	395	64.4 (9.2)	8.5 (4.6)	Not reported
	**SP715**		up to 6 years	258	66.4 (9.6)	7.8 (4.5)	
**Reinoso 2014**		**Design:** retrospective **Aim:** To understand the natural clinical evolution of treated PD patients and to identify the variables that predict greater progression in these patients. **Population:** Singapore	9 years	576	63.67 (10.39)	Not reported	2.13 (0.69)
**Schrag 2007**		**Design:** nonrandomized, prospective **Aim:** To assess the rate of clinical progression of PD **Population:** United Kingdom	4 years	124	Not reported	Not reported	2.2 (1.0)

The data extracted from the studies included in the analysis is presented in Table 40.

Table 40.
Long-term UPDRS progression – data extraction

Study	UPDRS change [points per year]				Note
	I	II	III	IV	
**Alves 2005**	-	1.900	3.300	-	General PD group; data reported directly in publication
**Brooks 2008**	-	-	1.000	-	Patients from clinical study treated with entacapone; data reported directly within study; value refers to period when initial improvement run out
**Jankovic 2001**	0.167	0.560	0.704	-	General PD group; data reported directly in publication
**LeWitt 2013**	-	1.799	1.967	-	Patients from clinical studies treated with levodopa or rotigotine; baseline value (since disease start progress and at the end of follow-up) taken from graph; annual change for 2 groups calculated and then averaged
**Reinosso 2014**	-	-	2.120	-	Data from period 7-9 years after treatment initiation; data reported directly in publication
**Schrag 2000**	-	-	0.378	-	Data from clinic based group with Hoehn&Yahr score 3-5; in study percentage change was reported (0.35%) and here is transformed into point change (0.35% x 108 [UPDRS III range])

Final model assumptions are presented in section B.2.

A.4. Mortality

A.4.1. Search strategy

In order to identify data on mortality in PD patients Medline database (via PubMed) was systematically searched. Search was performed in two steps:

1. Firstly, other systematic reviews were identified;
2. Secondly, a systematic search was done in order to identify studies publicized after date of search in latest review identified in step 1.

Detailed search strategy for other systematic reviews is presented in Table 41. This search was done on 25th March 2015.

Table 41.
Search strategy for mortality data in Medline database – systematic reviews

Search	Query	Items found
#5	(#1 AND #2 AND #3) AND “systematic review”	30
#4	#1 AND #2 AND #3	1432
#3	epidemiol* OR cross-section OR cross-sectional OR "cross sectional" OR register OR population-based OR prospective OR retrospective OR registry OR registries OR observational OR longitudinal OR database	2873113
#2	mortality OR survival OR death	1826331

Search	Query	Items found
#1	(Parkinson's disease) OR ("Parkinson Disease"[Mesh]) OR (Parkinson*) OR (parkinsonism OR (parkinsonian syndrome)) OR (paralysis agitans)	97201
	**Search strategy executed on 25th March 2015**	

Out of 30 abstracts identified in the search, 2 publications (describing systematic reviews) were included in full text analysis [297, 298]. The latest systematic review identified was performed by MacLeod 2014. As MacLeod 2014 review was also more comprehensive than the second systematic review identified [297], only MacLeod 2014 review was included in the analysis. Detailed methodology and search strategy used by MacLeod 2014 is presented in the publication.

As the date of last search in MacLeod 2014 review was October 2012, we did an additional search in order to identify other studies published after this date. In order to minimize possible search bias a limitation for studies published since January 2012 was done. Search strategy is presented in Table 42. The date of this search was 23th April 2015. Additionally, a non-systematic search via google was done.

Table 42.
Search strategy for mortality data in Medline database – primary studies

Search	Query	Items found
#5	(#1 AND #2 AND #3) Filters: Publication date from 2012/01/01	348
#4	#1 AND #2 AND #3	1443
#3	epidemiol* OR cross-section OR cross-sectional OR "cross sectional" OR register OR population-based OR prospective OR retrospective OR registry OR registries OR observational OR longitudinal OR database	2894977
#2	mortality OR survival OR death	1837299
#1	(Parkinson's disease) OR ("Parkinson Disease"[Mesh]) OR (Parkinson*) OR (parkinsonism OR (parkinsonian syndrome)) OR (paralysis agitans)	97726
	**Search strategy executed on 23th April 2015**	

A.4.2. Study selection

All studies identified within the Medline database search (studies published from 2012 onwards) were first analysed based on predefined selection criteria described in Table 43.

Once model structure was decided selection criteria were narrowed to include only studies examining association between UPDRS and mortality rate. At this stage, the studies included in MacLeod 2014 review were analysed. The information provided in MacLeod 2014 review allowed to identify relevant studies (assessing dependence between UPDRS and mortality), and so the study selection was based on information provided in MacLeod 2014 review. Similarly, an additional non-systematic

search via google was aimed at identifying only studies assessing relationship between UPDRS and mortality.

Table 43.
Selection criteria for studies on mortality data

Criterion	Inclusion	Exclusion
**Population**	• Patients with Parkinson's disease	• Other
**Data type**	• Data allowing to calculate mortality during treatment with one of following intervention: DBS, BMT, CSAI, CDLCI.<br>• Data allowing for determining relationship between mortality and progression assessed with one of following: H&Y, UPDRS.<br>• Data allowing to calculate mortality in general with PD.	• Lack of data specified in inclusion criteria.
**Study design**	• Observational studies,<br>• Registries.	• RCT studies,<br>• Case series.
**Publication date / other criteria**	• Not relevant (up to date of the search - 23th April 2015)<br>• Publicized in English	–

A brief summary of additional search is presented in Table 44.

Table 44.
Summary of systematic search for mortality data – additional search

Data source	No. of abstracts returned	Initial selection		Final no. of included studies
		No. included in full text analysis	No. of included studies	
**Medline (additional search)**	348	43	18	0

Details on selection of studies included in MacLeod 2014 review are presented in Table 45.

Table 45.
Summary of systematic search for mortality data – studies included in MacLeod 2014 review

Data source	No. of studies included in the review	No. included in full text analysis	Final no. of included studies
**MacLeod 2014 review**	88 unique studies described in 176 publications	3[a]	2

a) only publications reporting information on relationship between UPDRS and mortality (as indicated by information provided in MacLeod 2014 review) were included

Additionally one study was identified in non-systematic search via google and this study was also included in the analysis [299].

The diagram below (Figure 4) provides information on selection process for:

- 3 publications (out of 176) included in MacLeod 2014 review, that were selected for full text analysis based on information provided in MacLeod 2014 review,
- 348 publications identified through additional search in Pubmed (via Medline), to identify studies published after MacLeod 2014.,
- 1 publication identified in non-systematic search via google.

Figure 4.
Overview of studies selection: PRISMA flowchart – mortality data

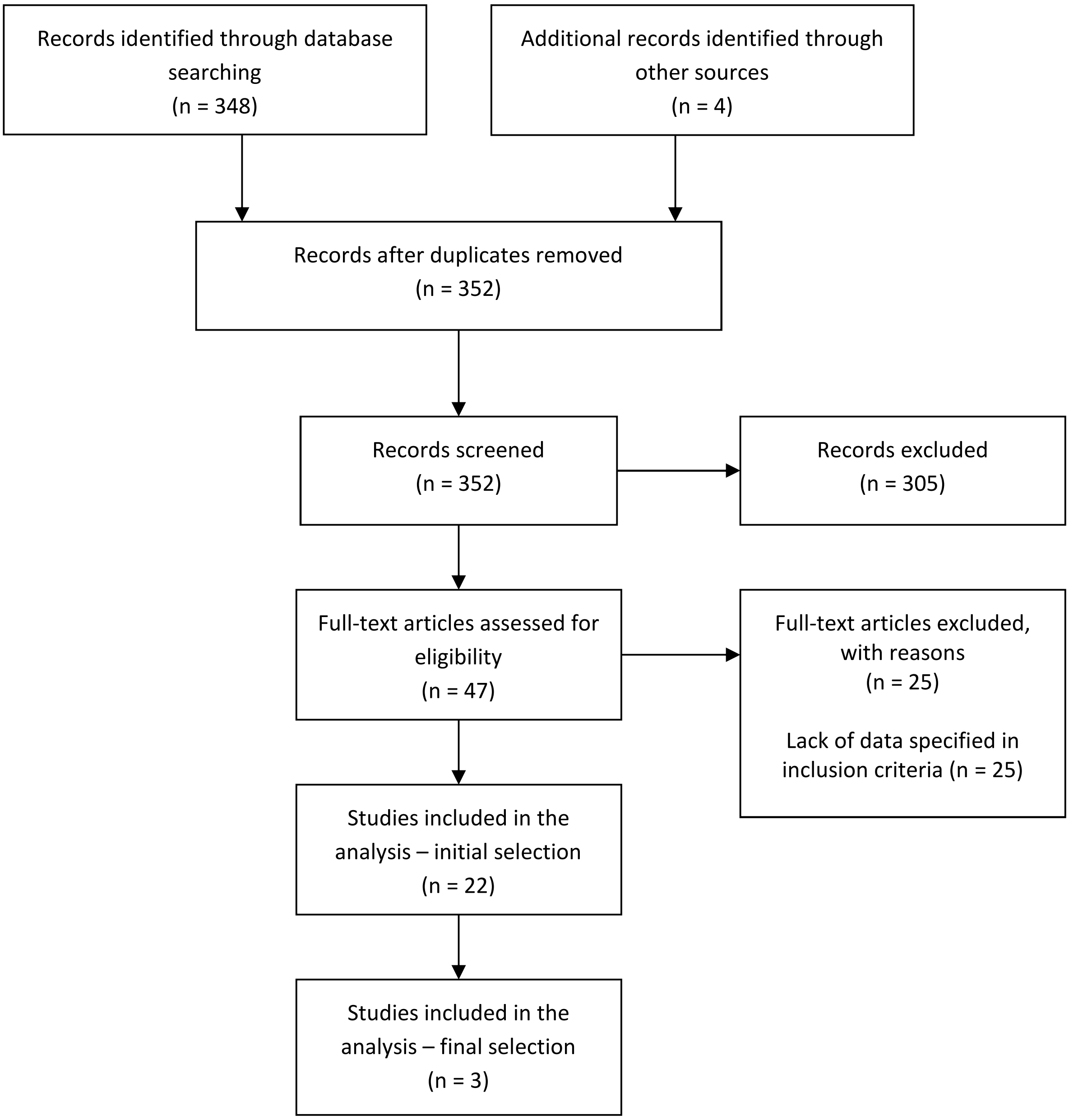

List of publications finally included in the analysis is presented in Table 46.

Table 46
Studies reporting association between UPDRS and mortality included in final analysis

Parameter	Mortality
**Publications included**	Marras 2005 [300], Forsaa 2010 [301], Skorvanek 2013 [299]
**Total number of publications**	**3**

List of publications included after initial selection, but finally excluded from the analysis is presented in Table 47.

Table 47.
Studies on mortality not included in final calculation (included after initial selection)

Publication	Reason of exclusion
**Jones 2012 [302]**	association between UPDRS and mortality not examined in the study
**Auyeung 2012 [303]**	association between UPDRS and mortality not examined in the study
**Benito-Leon 2014 [304]**	association between UPDRS and mortality not examined in the study
**Chillag-Talmor 2013 [305]**	association between UPDRS and mortality not examined in the study
**Desesquelles 2014 [306]**	association between UPDRS and mortality not examined in the study
**Duarte 2013 [307]**	association between UPDRS and mortality not examined in the study
**Duarte 2015 [254]**	association between UPDRS and mortality not examined in the study
**Frandsen 2014 [308]**	association between UPDRS and mortality not examined in the study
**Hobson 2010 [309]**	association between UPDRS and mortality not examined in the study
**Hoyert 2012 [310]**	association between UPDRS and mortality not examined in the study
**Kaltenboeck 2012 [311]**	association between UPDRS and mortality not examined in the study
**Mackenbach 2014 [312]**	association between UPDRS and mortality not examined in the study
**Merola 2014 [149]**	association between UPDRS and mortality not examined in the study
**Morgan 2014 [313]**	association between UPDRS and mortality not examined in the study
**Oosterveld 2015 [314]**	association between UPDRS and mortality examined, but reported results not useful for current analysis purposes
**Peretz 2014 [315]**	association between UPDRS and mortality not examined in the study
**Vu 2012 [293]**	association between UPDRS and mortality examined, but only total UPDRS or UPDRS II considered, whereas in the model only association between UPDRS III and mortality was taken into account (as most commonly reported)
**Williams-Gray 2013 [294]**	association between UPDRS and mortality not examined in the study
**Willis 2012 [316]**	association between UPDRS and mortality not examined in the study

A.4.3. Data extraction

Studies finally included in the analysis together with data extracted from the studies are presented in Table 48.

Table 48
Association between UPDRS III and mortality – data extraction

Study	No of patients	Hazard ratio per 10 points increase	Comment
**Marras 2005**	800	1.30	associations between baseline variables of interest and time to death
**Forsaa 2010**	230	1.25	associations between variables with mortality adjustment for age
**Skorvanek 2013**	153	1.48	only poster for this study was available

Final model assumptions are presented in section B.7.

APPENDIX B. DATA ANALYSES FOR MODEL INPUT

B.1. CSAI and CDLCI short-term effectiveness (first 2 years in the model)

The following sections present detailed information on the analysis of CSAI and CDLCI short-term effectiveness data (UPDRS) that was performed to derive values for short-term effectiveness parameters (first 2 years in the model) included in the CE model for these therapies. Data included in the analyses are from studies identified in the systematic review of both therapies (details in section A.1).

B.1.1. CSAI

UPDRS I

The Drapier 2012 study indicates a 17% improvement with CSAI in part I of the UPDRS scale after 1 year of treatment vs baseline.

Table 49.
CSAI effectiveness– UPDRS I – data included in the analysis

Study	Baseline score (no of patients)	Percentage change vs baseline (no of patients)
		1 year
**Drapier 2012**	2.90 (23)	-17.24% (23)

The Drapier 2012 study is the only study reporting results (only after 1 year of CSAI treatment) for mentation, behaviour, and mood section of UPDRS. Thus, validation of its result and assessment of effectiveness of CSAI at other time points is not possible. In economic analysis it was conservatively assumed that improvement in UPDRS I score is seen immediately after treatment initiation and is maintained for 2 years of treatment (year 1 data used to populate CE model). The final model assumptions are presented in Table 50.

Table 50.
CSAI effectiveness – UPDRS I – CE model assumptions

Parameter	Treatment initiation		Year 1		Year 2		Source
	Mean	SE	Mean	SE	Mean	SE	
**UPDRS I – percentage change from baseline**	-17.24%	3.92%	-17.24%	3.92%	-17.24%	3.92%	Drapier 2012

UPDRS II

The Drapier 2012 study indicates a 8% worsening with CSAI in part II of the UPDRS scale after 1 year of treatment vs baseline.

Table 51.
CSAI effectiveness– UPDRS II – data included in the analysis

Study	Baseline score (no of patients)	Percentage change vs baseline (no of patients)
		1 year
**Drapier 2012**	10.20 (23)	7.84% (23)

Drapier 2012 study is the only one study reporting results (only after 1 year of CSAI treatment) for the activities of daily living section of UPDRS. Thus, validation of its result and assessment of effectiveness of CSAI at other time points is not possible. In economic analysis it was conservatively assumed that there is no immediate change in UPDRS II score after treatment initiation and percentage change after 2 years of treatment is the same as after the first year. The final model assumptions are presented in Table 52.

Table 52.
CSAI effectiveness – UPDRS II – CE model assumptions

Parameter	Treatment initiation		Year 1		Year 2		Source
	Mean	SE	Mean	SE	Mean	SE	
**UPDRS II – percentage change from baseline**	0.00%	0.00%	7.84%	3.78%	7.84%	3.78%	Drapier 2012

UPDRS III

CSAI effectiveness data on the UPDRS part III scale were reported in 3 studies – results after 1 year and 2 years of treatment are available Data on percentage changes in the UPDRS III vs baseline calculated from these studies are presented in Table 53.

Table 53.
CSAI effectiveness– UPDRS III – data included in the analysis

Study	Baseline score (no of patients)	Percentage change vs baseline (no of patients)	
		1 year	2 years
**Drapier 2012**	18.30 (23)	19.1% (23)	-
**de Gaspari 2006**	19.50 (13)	-1.3% (13)	-
**Todorowa 2013**	33.00 (20)	-	-56.1% (15)

Results of the included studies are not consistent. Two of them indicate that there is no improvement / only slight improvement after first year of treatment, while results of third study show significant improvement after 2 years of treatment. A possible explanation for this observation might be the different baseline levels of UPDRS III scores. The results of the Kanovsky 2002 study [16], showing an 45% improvement in UPDRS motor score after 6 months of treatment up to 2 years in patients with average baseline UPDRS III score of 29.7, support the conclusion that higher effectiveness is reached in more advanced patients. As no information about UPDRS measurement condition (on or off meds) was provided in Kanovsky 2002, this study was excluded from the quantitative analysis.

Finally, in economic analysis only data from Todorowa 2013 were used. It was conservatively assumed that improvement in UPDRS III score is seen immediately after treatment initiation and is maintained for 2 years of treatment (year 2 data used to populate CE model). The final model assumptions are presented in Table 54.

Table 54.
CSAI effectiveness – UPDRS III – CE model assumptions

Parameter	Treatment initiation		Year 1		Year 2		Source
	Mean	SE	Mean	SE	Mean	SE	
**UPDRS III – percentage change from baseline**	-56.1%	2.35%	-56.1%	2.35%	-56.1%	2.35%	Todorowa 2013

UPDRS IV

CSAI effectiveness data on the UPDRS part IV scale were reported in 5 studies and data for 3 time points are available. Data on percentage changes in the UPDRS IV vs baseline calculated from these studies are presented in Table 55.

Table 55.
CSAI effectiveness– UPDRS IV – data included in the analysis

Study	Baseline score (no of patients)	Percentage change vs baseline (no of patients)		
		6 months	1 year	2 years
**Drapier 2012**	7.70 (23)	-	-10.4% (23)	-
**Kanovsky 2002**	10.80 (12)	-50.0% (12)	-50.9% (12)	-50.0% (12)
**Martinez-Martin 2011**	10.00 (17)	-	-64.7% (17)	-
**Martinez-Martin 2014**	10.02 (43)	-40.8% (43)	-	-
**Todorowa 2013**	11.30 (20)	-	-	-54.0% (15)

All studies reported improvement in UPDRS IV score in all time points considered. For the purposes of CE model, the effectiveness was estimated as follows:

1. The weighted average % change at 1 year of treatment vs baseline (the most commonly considered time point in studies included in the analysis) was calculated based on the results of 3 studies reporting respective data – 37.50%.
2. The Kanovsky 2002 study was the only study with more than one time point and so results of this study were used to calculate the % change at 2 years of treatment relative to year 1 – 1.89% worsening after 2 years when compared to year 1 results (details in Table 56):

Table 56.
CSAI effectiveness – UPDRS IV – relative effectiveness (1 year vs 2 years) data

Study	Parameter	Baseline (no of patients)	1 year (no of patients)	2 years (no of patients)
Kanovsky 2002	Mean value	10.80 (12)	5.30 (12)	5.40 (12)
	% change	-	-	1.89% (vs 1 year)

The estimated weighted average % change vs baseline at 1 year of treatment (-37.50%) and the % change at 2 years relative to year 1 (1.89% worsening) were combined to calculate the % change at 2 years of treatment vs baseline – -36.22% [(1 - 0.37.5%) x (1 + 1.89%) – 1].

3. No data on initial effectiveness (immediately after treatment initiation) were identified. The results of 2 studies included in the analysis show that improvement in UPDRS IV is achieved as soon as after 6 months of treatment (see Table 55). It was conservatively assumed that initial effectiveness is the same as after first year of treatment.

The final model assumptions are presented in Table 57.

Table 57.
CSAI effectiveness – UPDRS IV – CE model assumptions

Parameter	Treatment initiation		Year 1		Year 2		Sources
	Mean	SE	Mean	SE	Mean	SE	
UPDRS IV – percentage change from baseline	-37.50%	3.86%	-37.50%	3.86%	-36.32%	1.47%	Drapier 2012<br>Kanovsky 2002<br>Martinez-Martin 2011

B.1.2. CDLCI

UPDRS I

CDLCI effectiveness data on the UPDRS part I scale were reported in 2 studies. Only results after 1 year of treatment were available, for which percentage changes to baseline were calculated (Table 58).

Table 58.
CDLCI effectiveness– UPDRS I – data included in the analysis

Study	Baseline score (number of patients)	Percentage change vs baseline (no of patients)
		1 year
**Fernandez 2014[a]**	2.20 (288)	0.00% (272)
**Olanov 2014 / Slevin 2015[b]**	1.80 (36)	16.7% (33)
**Olanov 2014 / Slevin 2015[c]**	1.80 (33)	38.9% (26)

a) In Fernandez 2014 number of patients not reported at 1 year time point for UPDRS I data, number of patients with UPDRS I data assumed the same as number of patients with completed 1 year of follow-up – 272;
b) patients randomized to CDLCI at baseline who continued CDLCI treatment for 12 + 52 weeks (baseline score based on baseline data, 12 + 52 weeks follow-up data used as proxy for 1 year results);
c) patients randomized to BMT at baseline who started CDLCI after 12 weeks and continued CDLCI for further 52 weeks (baseline score based on 12-week data, 1-year results based on 12 + 52 weeks follow-up data)

The results of the Fernandez 2014 study indicate that there is no change in the UPDRS I after 1 year of treatment vs baseline. In Olanov 2014/Slevin 2015, worsening of UPDRS I was found.

In the economic analysis, the weighted average % change after first year of treatment vs baseline was calculated based on data from all studies reporting respective data (Table 58): 4.72%. It was conservatively assumed that UPDRS I score does not change immediately after treatment initiation. Effectiveness after 2 years of treatment was assumed to be the same as after 1 year of treatment. The final model assumptions are presented in Table 59.

Table 59.
CDLCI effectiveness – UPDRS I – CE model assumptions

Parameter	Treatment initiation		Year 1		Year 2		Sources
	Mean	SE	Mean	SE	Mean	SE	
**UPDRS I – percentage change from baseline**	0.00%	0.00%	4.72%	0.90%	4.72%	0.90%	Fernandez 2014 Olanov 2014/Slevin 2015

UPDRS II

CDLCI effectiveness data on the UPDRS part II scale were reported in 5 studies and data for 5 different time points are available. Data on percentage changes in the UPDRS II vs baseline calculated from these studies are presented in Table 60.

Table 60.
CDLCI effectiveness– UPDRS II – data included in the analysis

Study	Baseline score (no of patients)	Percentage change vs baseline (number of patients)				
		4 weeks	12 weeks	6 months	1 year	2 years
**Antonini 2013**	14.78 (73)	-	-	-28.1% (53)	-20.0% (43)	-5.7% (33)
**Antonini 2014 (GLORIA)**	16.50 (172)	-	-	-12.7% (69)	-18.8% (56)	-
**Caceres-Redondo 2014**	14.50 (16)	-	-	-	-	13.8% (16)

Study	Baseline score (no of patients)	Percentage change vs baseline (number of patients)				
		4 weeks	12 weeks	6 months	1 year	2 years
**Fernandez 2014**	17.40 (293)	-32.2% (286)	-31.0% (279)	-30.5% (269)	-24.1% (251)	-
**Olanov 2014 / Slevin 2015[a]**	11.60 (36)	-	15.5% (35)	-	4.3% (33)	-
**Olanov 2014 / Slevin 2015[b]**	11.80 (33)	-	-	-	-8.5% (26)	-

a) patients randomized to CDLCI at baseline who continued CDLCI treatment for 12 + 52 weeks (baseline score based on baseline data, 12 + 52 weeks follow-up data used as proxy for 1 year results);
b) patients randomized to BMT at baseline who started CDLCI after 12 weeks and continued CDLCI for further 52 weeks (baseline score based on 12-week data, 1-year results based on 12 + 52 weeks follow-up data)

Results of 3 studies reporting data for more than one time point indicate that CDLCI treatment positively affects UPDRS II score, however the effect diminishes over time (Antonini 2013, Fernandez 2014, Olanov 2014 / Slevin 2015). On the other hand, results from Antonini 2014 indicate that CDLCI effectiveness after 1 year of treatment is higher than after 6 months of treatment. Two data sets reporting results at single time points – Caceres-Redondo 2014, Olanov 2014 / Slevin 2015 (in group of patients randomised to CDLCI at baseline) – show deterioration in activities of daily living aspects after 2 years and 1 year of CDLCI treatment, respectively.

For the purposes of CE model, the effectiveness was estimated as follows:

1. The weighted average % change after 1 year of treatment vs baseline (the most commonly considered time point in studies included in the analysis) was calculated based on the results of 4 studies (5 groups of patients) reporting respective data – -19.68%.
2. The Fernandez 2014 study results were used to calculate % change at 1 year of treatment relative to initial results – 11.86% worsening after 1 year when compared to initial results (details in Table 61):

Table 61.
CDLCI effectiveness – UPDRS II – relative effectiveness (treatment initiation vs 1 year) data

Study	Parameter	Baseline (no of patients)	Treatment initiation (no of patients)	1 year (no of patients)
**Fernandez 2014**	**Mean value**	17.40 (293)	11.80 (286)	13.20 (251)
	**% change**	-	-	11.86% (vs initial)

The estimated average % change after 1 year of treatment vs baseline (-19.68%) and % change at 1 year of treatment relative to initial results (11.86% worsening) were combined to calculate % change at treatment initiation vs baseline – -28.20% [(1 – 19.68%) / (1 + 11.86%) – 1].

3. The Antonini 2013 study results were used to calculate % change at 2 years of treatment relative to year 1 – 17.92% worsening after 2 years when compared to year 1 results (details in Table 62):

Table 62.
CDLCI effectiveness – UPDRS II – relative effectiveness (1 year vs 2 years) data

Study	Parameter	Baseline	1 year	2 years
Antonini 2013	Mean value	14.78 (73)	11.83 (43)	13.95 (33)
	% change	-	-	17.92% (vs 1 year)

The estimated average % change after 1 year of treatment vs baseline (-19.68%) and % change at 2 years of treatment relative to year 1 (17.92% worsening) were combined to calculate % change after 2 years of treatment vs baseline – -5.29% [(1 – 19.68%) x (1 + 17.92%) – 1].

The final model assumptions are presented in Table 63.

Table 63.
CDLCI effectiveness – UPDRS II – CE model assumptions

Parameter	Treatment initiation		Year 1		Year 2		Sources
	Mean	SE	Mean	SE	Mean	SE	
UPDRS II – percentage change from baseline	-28.20%	0.17%	-19.68%	0.52%	-5.29%	2.13%	Antonini 2013<br>Antonini 2014 (GLORIA)<br>Fernandez 2014<br>Olanov 2014 / Slevin 2015

UPDRS III

CDLCI effectiveness data on the UPDRS part III scale were reported in 10 studies (11 publications). In 5 studies, CDLCI effectiveness was assessed at a single time point only. Data on percentage changes in the UPDRS III vs baseline calculated from all studies included in the analysis are presented in Table 64.

Table 64.
CDLCI effectiveness in clinical studies – UPDRS III – data included in the analysis

Study	Baseline score (no of patients)	Percentage change (number of patients)				
		4 weeks	12 weeks	6 months	1 year	2 years
Antonini 2008	24.60 (22)	-	-	-	-3.3% (22)	0.8% (22)
Antonini 2013	25.34 (73)	-	-	-10.9% (55)	-7.9% (47)	6.7% (29)
Antonini 2014 (GLORIA)	26.50 (172)	-	-	-11.3% (87)	-12.5% (74)	-
Caceres-Redondo 2014	27.20 (16)	-	-	-	-	8.5% (16)
Fernandez 2014	28.80 (291)	-34.0% (286)	-30.6% (279)	-31.6% (269)	-27.8% (251)	-
Honig 2009	19.10 (22)	-	-	-39.3% (22)	-	-
Karlsborg 2010	36.80 (12)	-44.6% (12)	-	-	-	-

Study	Baseline score (no of patients)	Percentage change (number of patients)				
		4 weeks	12 weeks	6 months	1 year	2 years
**Olanow 2014 / Slevin 2015[a]**	18.10 (36)	-	-8.3% (35)	-	8.3% (33)	-
**Olanow 2014 / Slevin 2015[b]**	22.50 (33)	-	-	-	-2.2% (25)	-
**Palhagen 2012**	24.40 (27)	-	-	-	-11.9% (25)	-
**Sensi 2014**	35.50 (17)	-	-	-5.9% (17)	-	-

a) patients randomized to CDLCI at baseline who continued CDLCI treatment for 12 + 52 weeks (baseline score based on baseline data, 12 + 52 weeks follow-up data used as proxy for 1 year results);
b) patients randomized to BMT at baseline who started CDLCI after 12 weeks and continued CDLCI for further 52 weeks (baseline score based on 12-week data, 1-year results based on 12 + 52 weeks follow-up data)

All studies indicate an improvement in UPDRS III after CDLCI initiation (based on 4-week, 12-week and 6-month data). However, results of 3 studies (Antonini 2008, Antonini 2013, Caceres-Redondo 2014, Olanow 2014/Slevin 2015) showed worsening (in relation to baseline values) at further time points (1 year, 2 years), indicating that this effect diminishes over time. This is consistent with results of Zibetti 2013 [50] study (excluded from final calculations as data presented after mean follow-up).

For the purposes of CE model, the effectiveness was estimated as follows:

1. The weighted average % change after 1 year of treatment vs baseline (the most commonly considered time point in studies included in the analysis) was calculated based on the results of 6 studies (7 groups of patients) reporting respective data – -17.65%.
2. The Fernandez 2014 study results were used to calculate % change at 1 year of treatment relative to initial results (4 weeks results) – 9.47% worsening after 1 year when compared to initial results (details in Table 65):

Table 65.
CDLCI effectiveness – UPDRS III – relative effectiveness (treatment initiation vs 1 year) data

Study	Parameter	Baseline	Treatment initiation	1 year
**Fernandez 2014**	**Mean value**	28.80 (291)	19.00 (286)	20.80 (251)
	**% change**	-	-	9.47% (vs initial)

The estimated average % change after 1 year of treatment vs baseline (-17.65%) and % change at 1 year of treatment relative to initial results (9.47% worsening) were combined to calculate % change at treatment initiation vs baseline – -24.77% [(1 - 17.65%) / (1 + 9.47%) – 1].

3. The Antonini 2008 and Antonini 2013 study results were used to calculate % change at 2 years of treatment relative to year 1 – 17.92% worsening after 2 years when compared to year 1 results (weighted average, details in Table 66):

Table 66.
CDLCI effectiveness – UPDRS III – relative effectiveness (1 year vs 2 years) data

Study	Parameter	Baseline	1 year	2 years
Antonini 2008	Mean value	24.60(22)	23.80 (22)	24.80 (22)
	% change	-	-	4.20% (vs 1 year)
Antonini 2013	Mean value	25.34 (73)	23.33 (47)	27.05 (29)
	% change	-	-	15.95% (vs 1 year)
Weighted average % change [a]			-	10.88% (vs 1 year)

a) weighted with number of patients at 2 years

The estimated average % change after 1 year of treatment vs baseline (-17.65%) and % change at 2 years of treatment relative to year 1 (10.88% worsening) were combined to calculate % change after 2 years of treatment vs baseline – -8.69% [(1 – 17.65%) x (1 + 10.88%) – 1].

The final model assumptions are presented in Table 67.

Table 67.
CDLCI effectiveness – UPDRS III – CE model assumptions

Parameter	Treatment initiation		Year 1		Year 2		Sources
	Mean	SE	Mean	SE	Mean	SE	
UPDRS III – percentage change from baseline	-24.77%	0.21%	-17.65%	0.62%	-8.69%	3.55%	Antonini 2008<br>Antonini 2013<br>Antonini 2014 (GLORIA)<br>Fernandez 2014<br>Honig 2009<br>Olanow 2014 / Slevin 2015<br>Palhagen 2012

UPDRS IV

CDLCI effectiveness data on the UPDRS part IV scale were reported in 6 studies and data for 3 time points are available. Data on percentage changes in the UPDRS IV vs baseline calculated from these studies are presented in Table 68.

Table 68.
CDLCI effectiveness– UPDRS IV – data included in the analysis

Study	Baseline score (no of patients)	Percentage change (no of patients)		
		6 months	1 year	2 years
Antonini 2008	8.40 (22)	-	-23.8% (22)	-21.4% (22)
Caceres-Redondo 2014	8.70 (16)	-	-	-23.0% (16)
Honig 2009	10.50 (22)	-57.1% (22)	-	-
Martinez-Martin 2014	9.93 (44)	-56.1% (44)	-	-
Palhagen 2012	9.40 (27)	-	-39.4% (25)	-

Study	Baseline score (no of patients)	Percentage change (no of patients)		
		6 months	1 year	2 years
**Sensi 2014**	8.40 (17)	-33.3% (17)	-	-

All studies indicate an improvement in part IV of UPDRS. For the purposes of CE model, the effectiveness was estimated as follows:

1. The weighted average % change after 1 year of treatment vs baseline was estimated based on studies reporting 1-year outcomes and studies with 6-month outcomes indicating better therapeutic effect than seen at 1 year (Honig 2009, Martinez-Martin 2014) – -44.61%.
2. The Antonini 2008 study results were used to calculate % change at 2 years of treatment relative to year 1 – 3.12% worsening after 2 years when compared to year 1 results (details in Table 69):

Table 69.
CDLCI effectiveness – UPDRS IV – relative effectiveness (1 year vs 2 years) data

Study	Parameter	Baseline (no of patients)	1 year (no of patients)	2 years (no of patients)
**Antonini 2008**	**Mean value**	8.40 (22)	6.40 (22)	6.60 (22)
	**% change**	-	-	3.12% (vs 1 year)

The estimated average % change after 1 year of treatment vs baseline (-44.61%) and % change at 2 years of treatment relative to year 1 (3.12% worsening) were combined to calculate % change after 2 years of treatment vs baseline – -42.88% [(1 - 44.61%) x (1 + 3.12%) – 1].

3. It was conservatively assumed that initial % change vs baseline is the same as after first year of treatment (-44.61%).

The final model assumptions are presented in Table 70.

Table 70.
CDLCI effectiveness – UPDRS IV – CE model assumptions

Parameter	Treatment initiation		Year 1		Year 2		Sources
	Mean	SE	Mean	SE	Mean	SE	
**UPDRS III – percentage change from baseline**	-44.61%	1.29%	-44.61%	1.29%	-42.88%	0.41%	Antonini 2008<br>Honig 2009<br>Martinez-Martin 2014<br>Palhagen 2012<br>Sensi 2014

B.2. Long-term effectiveness/progression

Long-term UPDRS outcomes were modelled based on data identified in the systematic reviews. (details in Appendix A). For CSAI and CDLCI, no treatment specific long-term UPDRS data were identified. The analyses of BMT and DBS data are described in sections B.2.1 and B.2.2. Final model assumptions are presented in section B.2.3.

B.2.1. UPDRS "natural disease progression" – data from "BMT studies"

Results of studies identified in the systematic review (details in section A.3) and included in the analysis are summarised in the table below. Study results were used to calculate a UPDRS change (points change) per year.

Data on UPDRS IV progression were not identified. In some studies data on time to occurrence of motor complications or dyskinesia are shown based on Kaplan-Meier curves. In Hely 2005 [265] study additional data on ON/OFF periods occurrence are presented, including its severity. In economic analysis by Palmer 2002 [317] also a half-year probability for ON/OFF intensification increase were used. However, all these data are not sufficient to estimate change in total part IV of UPDRS over time. Therefore, data from EARLYSTIM (BMT arm) were also considered for estimation of UPDRS IV progression in the model, and for the analysis to derive UPDRS I-III progression rates (see Table 71).

Table 71.
Long-term UPDRS progression in BMT studies – data used in the analysis

Study	UPDRS change [points per year]				Note
	I	II	III	IV	
**Alves 2005**	-	1.900	3.300	-	General PD group; data reported directly in publication
**Brooks 2008**	-	-	1.000	-	Patients from clinical study treated with entacapone; data reported directly within study; value refers to period when initial improvement run out
**Jankovic 2001**	0.167	0.560	0.704	-	General PD group; data reported directly in publication
**LeWitt 2013**	-	1.799	1.967	-	Patients from clinical studies treated with levodopa or rotigotine; baseline value (since disease starts to progress) and at the end of follow-up taken from graph; annual change for 2 groups calculated and then averaged
**Reinosso 2014**	-	-	2.120	-	Data from period 7-9 years after treatment initiation; data reported directly in publication
**Schrag 2000**	-	-	0.378	-	Data from clinic based group with Hoehn&Yahr score 3-5; in study percentage change was reported (0.35%) and here is transformed into point change (0.35% x 108 [UPDRS III range])
**EARLYSTIM BMT arm**	0.235	0.300	0.650	0.355	Calculated as difference between baseline and 2-year value divided by follow-up period [2 years]
**Mean***	**0.201**	**1.136**	**1.447**	**0.355**	

* not weighted mean as number of patients in the studies not always reported.

B.2.2. UPDRS long-term progression in DBS studies

Data on UPDRS long-term outcomes were also collected from DBS studies, in the systematic review described in section A.2. Only studies reporting UPDRS scores at least at two follow-up time-points with first of them at least 2 year after treatment initiation were included in the analysis (other selection criteria listed in section A.2.2). Data used in the analysis are summarised in Table 72, Table 73, Table 74, Table 75. In order to avoid using the same patient group data more than once, publications were collected into groups describing the same data and considered as one study.

Table 72.
DBS long-term effectiveness - UPDRS I – data used in the analysis

Study	Mean UPDRS I (no of patients)						
	2 yrs.	3 yrs.	4 yrs.	5 yrs.	6 yrs.	7 yrs.	10 yrs.
Gan 2007 / Gervais-Bernard 2009		2.40 (36)		2.57 (23)			
Hung 2013	2.90 (60)			3.30 (31)		3.80 (17)	
Kim 2013	2.90 (52)	3.20 (52)	3.60 (<52)[a]	4.40 (<52)[a]	4.50 (<52)[a]	3.00 (<52)[a]	
Kishore 2010		1.20 (36)		2.10 (29)			
Weaver 2012	2.88 (157)	3.23 (159)					
Schupbach 2005	2.00 (32)			3.30 (30)			
Janssen 2014				3.10 (18)			4.20 (12)
Wider 2008	2.60 (36)			3.70 (37)			

a) exact number of patients not reported (for the purposes of the analysis 52 assumed at all time points)

Table 73.
DBS long-term effectiveness - UPDRS II (on med / on stim) – data used in the analysis

Study	Mean UPDRS II (no of patients)							
	2 yrs.	3 yrs.	4 yrs.	5 yrs.	6 yrs.	7 yrs.	8 yrs.	10 yrs.
Aviles-Olmos 2014				13.20 (41)			15.20 (12)	
Gan 2007 / Gervais-Bernard 2009		10.40 (36)	11.35 (23)					
Krack 2003		10.70 (40)		14.00 (39)				
Kim 2013	10.30 (52)	10.70 (52)	11.10 (<52)[a]	12.50 (<52)[a]	12.80 (<52)[a]	12.70 (<52)[a]		
Kishore 2010		7.30 (36)		7.10 (29)				
Li 2013				10.90 (31)			16.70 (29)	
Weaver 2012	14.97 (155)	17.50 (157)						
Piboolnurak 2007		13.20 (33)		14.60 (17)				
Romito 2009		8.90 (20)		8.60 (20)				
Schupbach 2005	10.00 (32)			14.30 (30)				
Janssen 2014				14.30 (18)				20.40 (12)

Study	Mean UPDRS II (no of patients)							
	2 yrs.	3 yrs.	4 yrs.	5 yrs.	6 yrs.	7 yrs.	8 yrs.	10 yrs.
**Wider 2008**	12.80 (36)			18.90 (37)				

a) exact number of patients not reported (for the purposes of the analysis 52 assumed at all time points)

Table 74.
DBS long-term effectiveness - UPDRS III (on med / on stim) – data used in the analysis

Study	Mean UPDRS III (no of patients)							
	2 yrs.	3 yrs.	4 yrs.	5 yrs.	6 yrs.	7 yrs.	8 yrs.	10 yrs.
**Aviles-Olmos 2014**				23.90 (41)			28.70 (12)	
**Fasano 2010**		22.90 (20)		21.40 (20)			26.90 (20)	
**Gan 2007 / Gervais-Bernard 2009**		12.50 (36)		13.17 (23)				
**Krack 2003**		15.30 (40)		21.10 (39)				
**Kim 2013**	17.70 (52)	18.50 (52)	18.50 (<52) [a]	22.10 (<52) [a]	21.80 (<52) [a]	22.10 (<52) [a]		
**Kishore 2010**		14.70 (36)		16.50 (29)				
**Weaver 2012**	19.43 (146)	21.96 (143)						
**Paek 2013**	14.20 (41)	19.50 (41)						
**Piboolnurak 2007**		23.00 (33)		24.80 (17)				
**Schupbach 2005**	10.10 (32)			17.90 (30)				
**Janssen 2014**				21.70 (18)				28.70 (12)
**Toft 2011**	15.40 (110)	18.10 (89)	19.50 (52)	22.40 (32)				
**Wider / Aybek 2007**	27.70 (36)	25.10 (43)		30.60 (37)				

a) exact number of patients not reported (for the purposes of the analysis 52 assumed at all time points)

Table 75.
DBS long-term effectiveness - UPDRS IV – data used in the analysis

Study	Mean UPDRS IV (no of patients)						
	2 yrs.	3 yrs.	4 yrs.	5 yrs.	6 yrs.	7 yrs.	10 yrs.
**Gan 2007 / Gervais-Bernard 2009**		3.10 (36)		3.26 (23)			
**Hung 2013**	1.80 (60)			1.90 (31)		1.80 (17)	
**Kim 2013**	4.40 (52)	4.60 (52)	4.80 (<52) [a]	6.00 (<52) [a]	6.40 (<52) [a]	5.40 (<52) [a]	
**Merola 2014**		2.72 (174)		3.32 (174)			
**Weaver 2012**	4.56 (122)	4.21 (124)					
**Schupbach 2005**	3.20 (32)			5.10 (30)			
**Janssen 2014**				2.40 (18)			2.30 (12)

a) exact number of patients not reported (for the purposes of the analysis 52 assumed at all time points)

The data presented in the tables above were furtherly analyzed with use of linear regression (geeglm function from geepack package for R version 3.2.2) to derive long-term progression rates in patients treated with DBS (separately for each UPDRS score). Use of geeglm function allowed for estimation of single annual progression rate for each UPDRS score based on data from different groups of patients reported at multiple time points (not necessarily the same time points in all groups of patients), weighted with number of patients in each group that was changing over time (patients were lost to follow-up).

Table 76 shows calculations results for UPDRS long-term progression. The estimated mean annual progression rate for each UPDRS scores together with SE are presented. Additionally, statistical significance of results can be assessed based on p-value estimated. Assuming significance level of 0.005, it can be concluded that results for all UPDRS scores are statistically significant (p-value < 0.005), indicating that all UPDRS scores do not remain constant in the long-term setting.

Table 76.
Long-term UPDRS progression – results from regression analyses in DBS studies

Parameter	Annual rate of progression		
	Mean	Standard Error	p-value
**UPDRS I**	0.19301	0.02267	<0.005
**UPDRS II**	0.940	0.229	<0.005
**UPDRS III**	1.366	0.214	<0.005
**UPDRS IV**	0.2685	0.0551	<0.005

The reliability of the results presented above is limited. In most of the studies availability of the UPDRS data decreased with increasing follow-up – patients were lost to follow-up for various reasons and results at further time points were based on data with low number of patients. Consequently, UPDRS changes would possibly differ from those assumed in the analysis, if only results for patients with longer follow-up were taken into account also in earlier time points. However, given data availability, it was not possible to take into account the impact of withdrawals on reported changes in UPDRS scores.

Moreover, in none of the studies listed in above tables, statistical analysis of the changes in UPDRS scores over time was performed, except Schupbach 2005. The results reported in the Schupbach 2005 study (data presented in Table 75) suggest that UPDRS IV score after 5 years of treatment is almost 2 points worse than after 2 years (5.10 at year 5 vs 3.20 at year 2). However, in this analysis, the observed worsening in UPDRS IV was not statistically significant and so the results do not allow a rejection of the hypothesis that UPDRS IV remains constant in the long term setting.

B.2.3. CE model assumptions

Results of the analysis of long-term UPDRS progression in DBS and BMT studies are summarized in Table 77. The table contains also results of Krack 2003 study [128], one of the DBS studies included in the analysis, deemed to be representative for UPDRS III progression in population under consideration, based on clinical expert opinion. As mentioned before, long term evidence for CSAI and CDLCI was not identified.

Given the data availability, reliable assessment of possible changes in progression rates over time (depending on duration of disease, treatment, motor complication etc.) was not possible. Thus, long term UPDRS progression is expressed in terms of a constant annual progression rates.

Table 77.
Long-term UPDRS progression – results of the analysis

Source	Annual progression rate							
	UPDRS I		UPDRS II		UPDRS III		UPDRS IV	
	Mean	SE	Mean	SE	Mean	SE	Mean	SE
**BMT studies**	0.201	0.020	1.136	0.114	1.447	0.145	0.355 [b]	0.223 [b]
**DBS studies**	0.193	0.023	0.940	0.229	1.366	0.214	0.269	0.055
**Krack 2003 [a]**			1.650 [c]	0.335	2.425 [c]	0.595		

a) One of the DBS studies deemed to be representative for UPDRS III progression in population under consideration (clinical expert opinion)
b) Based solely on EARLYSTIM results (BMT arm)
c) Based on 1-year and 5-year follow-up data (see Table 30 and Table 31)

Based on clinical expert opinion, it was assumed that long term progression rates are the same, irrespective of treatment used (DBS, BMT, CSAI, CDLCI). Assumed progression rates (details below) are applied in the model from year 3 onwards, with the exception of UPDRS IV in DBS, CSAI and CDLCI patients. For UPDRS IV, the value from the EARLYSTIM trial (DBS arm) at year 2 is held constant (no progression) for a further 8 years in the DBS arm, and the same progression rate as for the BMT arm is applied thereafter. The basis for this assumption was expert opinion indicating that the main impact of DBS in reducing complications of therapy is maintained in the long-term. The same assumption (UPDRS IV at year 2 held constant for a further 8 years) was applied for CSAI and CDLCI.

The following data sources were used in base case analysis:

- data from BMT studies for UPDRS I & II (for all treatments arms from year 3 onwards),
- data from Krack 2003 [128] for UPDRS III (for all treatments arms from year 3 onwards) – assumption based on expert opinion,
- data from EARLYSTIM trial (BMT arm) for UPDRS IV (from year 3 onwards for the BMT arm, from year 11 onwards for the DBS, CSAI and CDLCI arms).

Due to high uncertainty in estimated progression rates alternative data sources were considered in the sensitivity analysis. Assumptions of base case scenario and sensitivity analysis scenarios are presented in Table 78.

Table 78
Long-term UPDRS progression – model input data

Parameter	UPDRS I		UPDRS II		UPDRS III		UPDRS IV	
	**Mean**	**SE**	**Mean**	**SE**	**Mean**	**SE**	**Mean**	**SE**
**Base case scenario**								
**Annual progression rate**	**0.201**	**0.020**	**1.136**	**0.114**	**2.425**	**0.595**	**0.355**	**0.223**
**Source**	**BMT studies**		**BMT studies**		**Krack 2003**		**EARLYSTIM**	
**Sensitivity analysis – scenario 1**								
**Annual progression rate**	0.201	0.020	1.650	0.335	2.425	0.595	0.355	0.223
**Source**	BMT studies		Krack 2003		Krack 2003		EARLYSTIM	
**Sensitivity analysis – scenario 2**								
**Annual progression rate**	0.193	0.023	0.940	0.229	1.366	0.214	0.355	0.223
**Source**	DBS studies		DBS studies		DBS studies		EARLYSTIM	
**Sensitivity analysis – scenario 3**								
**Annual progression rate**	0.201	0.020	1.136	0.114	1.447	0.145	0.355	0.223
**Source**	BMT studies		BMT studies		BMT studies		EARLYSTIM	
**Sensitivity analysis – scenario 4**								
**Annual progression rate**	0.201	0.020	1.136	0.114	2.425	0.595	0.178	-
**Source**	BMT studies		BMT studies		Krack 2003		2-fold deceleration of progression in relation to EARLYSTIM data	
**Sensitivity analysis – scenario 5**								
**Annual progression rate**	0.201	0.020	1.136	0.114	2.425	0.595	0.710	-
**Source**	BMT studies		BMT studies		Krack 2003		2-fold acceleration of progression in relation to EARLYSTIM data	

Assumptions that were changed in sensitivity analysis (as compared to base case scenario) underlined

Additionally, scenarios assuming different response duration for DBS, CSAI and CDLCI were explored in the sensitivity analysis:

- for UPDRS I-III: value at year 2 held constant (no progression) for a further 2 years, and the same progression rate as for BMT arm applied thereafter;
- for UPDRS IV: the same progression rate as for BMT arm applied from year 3 onwards.

B.3. Adverse events

Data on adverse events associated with DBS, CSAI and CDLCI were identified in the systematic review (details in Appendix A).

Due to high heterogeneity in reporting of adverse events in DBS studies identified in the systematic review, based on their results it was not possible to reliably estimate frequency of adverse events that influence patient's quality of life or generate costs (and thus are worth considering in the model). Data

on adverse events in the EARLYSTIM study (both published and unpublished patient-level data) were used to estimate relevant inputs for the model, for both DBS and BMT. Detailed information on EARLYSTIM-based adverse event analysis is provided in section **Error! Reference source not found.**.

The following sections present data on skin nodules frequency in CSAI studies (section B.3.1) and peritonitis frequency in CDLCI studies (section B.3.2), included from the studies identified in the systematic review.

B.3.1. CSAI – skin nodules frequency

Studies reporting data on skin nodules / skin reaction frequency during CSAI treatment were identified in systematic review (details in section A.1). The data from studies included in the analysis are presented in Table 79. Based on these data weighted mean frequency of skin nodules/ skin reaction occurrence was calculated (74.56%). Given the lack of long term data, this rate was applied only within the 1st year of treatment.

Table 79.
Adverse event (skin nodules / skin reaction) in clinical trials – CSAI treatment

Study	Number of patients	Patients with events	% with events	Note
**Antonini 2011 / de Gaspari 2006**	12	2	17%	Based on Antonini 2011
**Drapier 2012**	23	23	100%	-
**Elia 2012**	10	5	50%	-
**Garcia-Ruiz 2008**	82	56	68%	In 7 patients with severe grade
**Hughes 1993 / Frankel 1990**	25	25	100%	Based on Frankel 1990
**Katzenschlager 2005**	12	9	75%	-
**Morgante 2004 / di Rosa 2003**	10	10	100%	Based on Morgante 2004
**Pietz 1998**	25	25	100%	-
**Poewe 1993**	18	4	22%	-
**Stibe 1988**	11	11	100%	-
**Total**	228	170	**74.56%**	
**SE**			**2.88%**	

Additionally in Stocchi 1993 , Stocchi 2001 and Manson 2002 skin nodules / skin reaction occurred in almost all patients but no detailed data were presented

B.3.2. CDLCI – peritonitis frequency

Due to the heterogeneity in reporting of adverse events in studies identified in the systematic review, the device related AEs rates were estimated based on Nyholm 2008 [45] as this study was also used in other economic analyses (Lowin 2011, Walter 2014 [318, 319]).

Data on peritonitis frequency during CDLCI treatment were retrieved from the studies identified in the systematic review, and the analysis of the data is presented in Table 80. Based on these data, a weighted mean frequency of peritonitis occurrence was calculated (3.23%). Given the lack of long term data, this rate was applied only within the 1st year of treatment.

Table 80.
Occurrence of peritonitis during CDLCI treatment – data from clinical studies

Study	Number of patients	Peritonitis	
		Number of events	Probability of event
**Antonini 2013d**	98	4	4.08%
**Devos 2009**	91	4	4.40%
**Fernandez 2014**	324	9	2.78%
**Martinez-Martin 2014**	44	1	2.27%
**Palhagan 2012**	37	1	2.70%
**Sensi 2014**	28	2	7.14%
**Zibetti 2014**	59	1	1.69%
**Total**	681	22	3.23%
**SE**			0.03%

B.4. Withdrawal

Data on withdrawal rates for CSAI and CDLCI were sourced from studies identified in the systematic review of CSAI and CDLCI efficacy (details in section A.1).

B.4.1. CSAI

Detailed data on treatment discontinuation (including information on follow-up period) were available in 9 studies (12 publications). Data on withdrawal rates in the studies included in the analysis are presented in Table 81. Only withdrawals due to adverse events, lack of efficacy or due to own patient decision were included in the calculations.

Table 81.
Discontinuation of CSAI treatment – data included in the analysis

Study	Total number of patients	Discontinuation		Follow-up [years]	Reasons for withdrawal
		Number of patients	% of patients		
**Antonini 2011 / de Gaspari 2006**	12	6	50.0%	5.00 / 2.50 [a / b]	AEs or no effectiveness (6)
**Drapier 2012**	23	0	0.0%	1.00 [a]	-
**Hughes 1993 / Frankel 1990**	25	6	24.0%	3.04 [b]	AEs (4), other (2)

Study	Total number of patients	Discontinuation		Follow-up [years]	Reasons for withdrawal
		Number of patients	% of patients		
**Katzenschlager 2005**	12	0	0.0%	0.50 [a]	-
**Manson 2002**	64	8	12.5%	2.82 [b]	AEs (3), patient's decision (2), unable to continue (3)
**Martinez-Martin 2014**	43	0	0.0%	0.50 [a]	-
**Morgante 2004 / di Rosa 2003**	12	1	8.3%	2.00 [a]	infection (1)
**Poewe 1993**	18	4	22.2%	1.72 [b]	AEs (3), no effectiveness (1)
**Todorova 2013**	20	5	25.0%	2.00 [a]	skin reaction (5)

a) total follow-up of the study; b) mean follow-up

The probability of treatment discontinuation was calculated based on data from all studies listed above. In the first step, yearly probability of discontinuation was calculated for each study (probability from de Gaspari 2006 was calculated based on mean follow-up period, data on total follow-up period was not used), assuming constant yearly withdrawal. In the second step, the weighted mean withdrawal probability was estimated for all studies. Values of weights were assumed to be equal to the product of number of patients and follow-up period in each study. A summary of calculations is presented in Table 82. The weighted mean was 7.59% (SD 0.12%) and this value was used in the model.

Table 82.
Discontinuation of CSAI treatment – model input data

Study	Annual discontinuation probability	Weight
**Antonini 2011 / de Gaspari 2006**	12.94%	30.00
**Drapier 2012**	0.00%	23.00
**Hughes 1993 / Frankel 1990**	8.63%	76.04
**Katzenschlager 2005**	0.00%	6.00
**Manson 2002**	4.63%	180.27
**Martinez-Martin 2014**	0.00%	21.50
**Morgante 2004 / di Rosa 2003**	4.26%	24.00
**Poewe 1993**	13.62%	30.90
**Todorova 2013**	13.40%	40.00
**Mean**	**7.59%**	-
**SD**	**0.12%**	-

B.4.2. CDLCI

Detailed data on treatment discontinuation were available in 6 studies. Moreover, one study – Nyholm 2012 [320] – was identified in non-systematic search. Detailed data sourced from all included studies are presented in Table 83. Discontinuation rates were estimated separately for initial phase / test phase of treatment, first year of treatment (initial phase excluded) and subsequent years of treatment, according to the information in the studies.

Table 83.
Discontinuation of CDLCI treatment – data included in the analysis

Study	Total number of patients	Discontinuation		Note
		Number of patients	% of patients	
**Test phase / initial treatment period**				
**Antonini 2014 (GLORIA)**	172	8	8.5%	**Test phase:** run-in period (approximately 7-14 days period for verification of effectiveness and dose) – CDLCI infusion via nasoduodenal tube **Discontinuation reason:** not reported in details
**Fernandez 2014**	354	22[a]	6.2%	**Test phase:** nasojejunal (NJ) titration period (2-14 days) and a PEG-J titration period (2-14 days) **Discontinuation reason:** withdrew consent (12), AEs (5), lack of efficacy (5)
**Nyholm 2008**	58	7	12.1%	**Test phase:** nasodoudenal test period for an average 12 days (range: 3-30) **Discontinuation reason:** not reported in details
**Palhagen 2012**	37	10	27.0%	**Test phase:** initiation nasoduodenal CDLCI + period before permanent CDLCI implantation (3 months) **Discontinuation reason:** withdrew consent (3), AEs (2), lack of efficacy (5)
**1st year of treatment**				
**Eggert 2008**	13	4	30.8%	**Discontinuation reason:** patient's refusing (1), AEs (1), mechanical and physical problems (2)
**Fernandez 2014**	324	37[b]	16.0%	**Discontinuation reason:** withdrew consent (13), AEs (22), lack of efficacy (2)
**Slevin 2005**	29	5	17.2%	**Discontinuation reason:** withdrew consent (13), AEs (22), lack of efficacy (2)
**Subsequent years**				
**Nyholm 2012**	135	31	23.0%[c]	**Discontinuation reason:** AEs or lack of effectiveness **Time period:** mean follow-up 4.2 years; restricted mean treatment time estimated with Kaplan–Meier methodology (censoring at the end of the study or death): 7.79 years

a) additional 8 patients withdrew due to administrative reason or protocol violation. According to methodology not included in calculation
b) additional 15 patients withdrew due to administrative reason or protocol violation. According to methodology not included in calculation
c) cumulative discontinuation rate for the whole follow-up period

In the Palhagen 2012 study, discontinuation rate reported for test phase in much higher than in other studies. This might be caused by fact that longer period was considered in this trial (3 months vs up to 1 month in other trials). So in the final calculations data from Palhagen 2012 were not included. Discontinuation rate in initial treatment period was calculated based on the results reported in Antonini

2014 (GLORIA), Fernandez 2014 and Nyholm 2008 – 6.34%. In the model it was assumed that only costs of trial tube insertion and removal are borne in patients who discontinue treatment in initial/test period. The effectiveness of treatment was not considered in such cases – it was assumed that treatment in test period has no effect on UPDRS scores (modelling of UPDRS scores is the same as in patients treated continuously with BMT).

The percentage of patients who stop treatment after treatment initiation but still within the first year of treatment was calculated based on data from all 3 studies identified – Eggert 2008, Fernandez 2014, Slevin 2005 – 12.57%.

Probability of treatment discontinuation in subsequent years was calculated based on Nyholm 2012. Cumulative discontinuation rate for the whole follow-up period (23.0% in 7.79 years) was rescaled to calculate annual discontinuation rate (assumed to be constant over time) – 3.29%.

Summary of discontinuation rates applied in the model is provided in Table 84.

Table 84
Discontinuation of CDLCI treatment – model input data

Treatment period	Discontinuation probability	
	Mean	SD
**Test phase / initial period**	6.34%	0.17%
**1st year**	12.57%	0.25%
**Year 2 and every year thereafter**	3.29%	0.13%

B.5. CSAI dose

Studies reporting data on CSAI dose were identified in the systematic review of CSAI efficacy (details in section A.1). Studies with dose presented as mean daily dose reported were included, whereas studies reporting data on dose as median, as dose per hour or in some specified subgroups of patients were excluded. Finally, 12 studies (described in 15 publications) were included in the analysis.

Mean daily dose of CSAI was calculated using data on mean dose after achieving optimal dosage or mean dose within study follow-up. In case data was available at several time points in the study, data from the last time point were used. This assumption has no effect on the results of the analysis as the differences in daily doses over time were not significant in such cases.

Detailed data from single studies used in calculation are presented below, together with mean daily dose weighted by number of patients.

Table 85.
Daily doses of CSAI in clinical studies

Study	Number of patients	Dose per day [mg]		Note
		Mean	SD	
**Antonini 2011 / de Gaspari 2006**	12	83.40	19.20	-
**Drapier 2012**	23	62.60	18.80	-
**Garcia-Ruiz 2008**	82	72.00	21.38	-
**Hughes 1993 / Frankel 1990**	22	80.80	31.60[a]	data from Hughes 1993 taken; mean dose after initial stabilization
**Katzenschlager 2005**	8	84.70	33.13[a]	data form UK patients group
**Manson 2002**	45	102.50	71.80	data from monotherapy group
**Martinez-Martin 2014**	43	105.90	23.20	-
**Morgante 2004 / di Rosa 2003**	12	100.00	39.11[a]	-
**Poewe 1993**	14	160.00	62.58[a]	data from patients with complete follow-up
**Stibe 1988**	11	77.00	30.12[a]	-
**Stocchi 2001**	30	51.60	34.80	-
**Todorova 2013**	20	105.30	23.90	-
**Weighted average**		**86.67**	**2.45**	

a) SD value not reported within the study; calculated based on relation of SD to mean from other studies (39.11%)

B.6. Falls

No separate systematic review was performed to identify data on falls in PD patients. Relevant data were selected from studies identified in the search for progression data in BMT studies (details in section A.3). Initial selection was based on titles and abstracts and only studies specifically aimed at assessing falls in PD patients were included in full text analysis. Out of 15 publications included in full text analysis, 7 were finally included in the analysis (excluded studies did not provide any data relevant for the analysis). Additionally, references from identified publications were analysed and non-systematic search via Google was performed. This allowed to identify another 9 studies.

Altogether, we found 16 studies reporting the % of falls among included PD patients. A brief characteristics of the studies is provided in Table 86 and data extracted from the studies – in Table 87.

Table 86.
Characteristics of studies included in falls data analysis

Study	Study design	Follow-up	No of patients	Age [mean]	Duration of PD [years]	Hoehn & Yahr	UPDRS III
**Ashburn 2001**	**Design:** prospective<br>**Aim:** To identify one or more features, which would predict individuals at risk of falling during follow-up<br>**Country:** UK	3 months	57	71 (46-86)	not reported	not reported	not reported
**Bloem 2001**	**Design:** prospective<br>**Aim:** To study the epidemiology, clinical impact and prediction of falls in moderately affected PD patients<br>**Country:** The Netherlands	6 months	59 with PD<br>55 control	60.8 (9.7)	7.1 (4.8)	2.3 (0.7)	31.5 (11.0)
**Cheng 2014**	**Design:** prospective<br>**Aim:** To analyze the clinical features, imaging findings, scientific clinical scores, and measurements to determine potential risk factors associated with fall-related fracture in PD<br>**Country:** Taiwan	18 months	100	68.6 (10.6)	5.06 (4.1)	2.7 (1.3)	34.96 (18.31)
**Contreras 2012**	**Design:** retrospective<br>**Aim:** To determine the relevant risk factors for falling in PD<br>**Country:** Spain	not reported	160	72.0 (9.5)	8.1 (6.4)	2.6 (1.0)	28.8 (15.6)
**Gazibara 2014**	**Design:** not reported<br>**Aim:** To estimate fall frequency as well as demographic and clinical related factors<br>**Country:** Serbia	6 months	180 fallers<br>120 non-fallers	62 median for fallers<br><br>60 median for non-fallers	7 for fallers<br>4 for non-fallers	2.5 – fallers<br>2 – non-fallers	45 – fallers<br>38 – non-fallers
**Johnson 2014**	**Design:** prospective<br>**Aim:** To determine the efficacy of clinical tests, balance scales, and stable-platform posturography in detecting postural instability and discriminating between fallers and non-fallers<br>**Country:** Australia	24 months	48	65 (8)	6	2.5 (0.9)	15.3
**Kataoka 2011**	**Design:** prospective<br>**Aim:** To evaluate factors responsible for falling in PD patients at H&Y III stage<br>**Country:** Japan	6 months	30	68.3 (7.0)	7.12 (5.78)	3 – all patients	19.3 (8.7)
**Kerr 2010**	**Design:** prospective<br>**Aim:** To determine an optimal combination of functional and disease-specific tests to predict falls in individuals with PD<br>**Country:** Australia	6 months	101	66.4 (8.2)	6.1 (4.4)	2.1 (0.8)	18.7 (9.2)

Study	Study design	Follow-up	No of patients	Age [mean]	Duration of PD [years]	Hoehn & Yahr	UPDRS III
**Latt 2009**	**Design:** not reported **Aim:** to devise a fall risk screen for people with PD using routine clinical measures and an explanatory (physiological) fall risk assessment for guiding fall prevention interventions **Country:** Australia	12 months	113	66.1	not reported	2.02	not reported
**Lindholm 2015**	**Design:** prospective **Aim:** To determine factors associated with future falls and/or near falls in people with mild PD. **Country:** Norway	6 months	141	68	2 - median	2 - median	13 - median
**Matinolli 2011**	**Design:** prospective **Aim:** To evaluate the risk factors for recurrent falling and mortality in PD **Country:** Finland	24 months	125	67.9 (10.2)	6.1 (4.1)	2.3 (0.7)	25.0 (11.1)
**Parashos 2012**	**Design:** prospective **Aim:** explore risk factors for falls in PD utilizing the cross-sectional, baseline data in the NPF-QII database **Country:** USA	3 months	2,876	68.1 (8.6)	7.6 (5.8)	2.41	not reported
**Rudzinska 2013**	**Design:** prospective **Aim:** To assess the incidence and risk factors of falls in comparison to a control group **Country:** Poland	12 months	100	67.2 (9.9)	6.2 (3.4)	2.75 (0.65)	32.2 (13.8)
**Voss 2012**	**Design:** not reported **Aim:** To define the frequency of falls in early PD and assess potential risk factors for falls in this population **Country:** USA	18 months	431	not reported	< 5 years	not reported	not reported
**Wielinski 2005**	**Design:** retrospective **Aim:** To ascertain frequency, type, risk factors of falling, and resulting injuries among parkinsonian patients **Country:** USA	24 months	1,092	72.7 - median	7.0 - median	not reported	not reported
**Wood 2002**	**Design:** prospective **Aim:** To accurately establish the incidence of falls in PD and to investigate predictive risk factors for fallers from baseline data **Country:** UK	6 months	101	75 - median	6 - median	1.84	not reported

Table 87.
Falls frequency – data included in the analysis

Study	Number of patients		% of patients with falls	Time period [months]	Total number of falls	Falls per patient-year
	Total	Fallers				
**Ashburn 2001**	57	22	39%	3	-	-
**Bloem 2001**	59	30	51%	6	205	6.95
**Cheng 2014**	100	56	56%	18	123[b]	0.82
**Contreras 2012**	160	62	39%	not reported	-	-
**Gazibara 2014**	300	180	60%	6	-	-
**Johnson 2014**	48	26	54%	24	-	-
**Kataoka 2011**	30	15	50%	6	-	-
**Kerr 2010**	101	48	48%	6	-	-
**Latt 2009** [a]	113 (total)	51	45%	12	2,160	19.12
	37 (UPDRS III <10)	11	30%		-	-
	43 (UPDRS III 10-19)	20	47%		-	-
	33 (UPDRS III >19)	20	61%		-	-
**Lindholm 2015**	141	45	32%	6	158	2.24
**Matinolli 2011**	125	79	63%	24	3,125	12.50
**Parashos 2012**	2,876	1,069	37%	3	-	-
**Rudzinska 2013**	100	54	54%	12	194	1.94
**Voss 2012**	431	93	22%	18	-	-
**Wielinski 2005**	1092	597	55%	24	-	-
**Wood 2002**	101	69	68%	6	585	11.58

a) result with division for total UPDRS also available within study; b) mean number of falls per faller in this study was 2.2 and total number of falls was calculated as 56 fallers multiplied by 2.2 falls

The falls data reported by different studies are not consistent and there is a big difference between low and high values. In order to explain differences between reported frequency of falls, patients characteristics in each study were analysed (Table 86). One possible explanation for difference in estimated percentage of fallers can be due to duration of studies – a higher value can be expected for those with longer study duration as they can cover more subjects in later stages of PD.

The following calculations and assumptions were made to arrive at the values for model analyses:

- We have calculated percentage of fallers within model cycle based on data extracted from all 16 studies, resulting in a mean value of 42.78%. As this value is similar to the value reported by Latt 2009 for UPDRS III 10-19 group – 47%, it was assumed that it can be applied to patients at baseline condition in our model (average baseline UPDRS III value of 12.3). Additionally we assumed that there is only one fall per faller.

- The fraction of injurious falls was assessed based on 3 studies. Calculated mean frequency of injurious falls as a percentage of all falls is 50.9% (Table 88) Injurious falls were assumed to lead to hospitalisations.

Table 88.
Frequency of injurious falls – data included in the analysis

Study	No of falls	% of injurious falls
**Rudzinska 2013**	194	40%
**Bloem 2001**	150	62%
**Contreras 2012**	62	58%
**Average**	**-**	**50.88%**

- The reviewed literature indicates that individual UPDRS I-IV scales as well as total UPDRS values are good predictors of the increase of falls frequency with PD progression [251, 259, 321, 322]. Most data were published for UPDRS III, so this scale was chosen as a predictive factor of PD progression related falls. From the list of reviewed papers, 3 publications containing changes of frequency of falls odds ratio per unit of UPDRS III score increase with data have been selected to extract information used in our model. The averaged OR based on these studies is 1.067.

Table 89.
Association between UPDRS III and falls frequency: Odds ratio – data included in the analysis

Study	Falls frequency – odds ratio per 1 point increase in UPDRS III	
	Mean	N
**Ashburn 2001**	1.09	57
**Kataoka 2011**	1.06	30
**Contreras 2012**	1.06	160
**Average**	**1.067**	**247**

A summary of final assumptions of the analysis with regard to falls is presented in the table below.

Table 90
Data on falls in PD – model input data

Parameter	Value	
	Mean	SD
**Percentage of patients who experience falls [a]**	42.78%	0.92%
**Annual number of falls per faller [a]**	1	-
**Percentage of injurious falls (= falls requiring hospitalization) [a]**	50.88%	2.48%
**Average annual probability of fall requiring hospitalization [a]**	**21.77%**	**-**
**OR for falls frequency per 1 point increase in UPDRS III [b]**	1.067	0.031

a) parameters estimated for patients with average UPDRS III value of 12 (reference value for application of OR)
b) UPDRS III value of 12 as reference (increased/decreased falls frequency for UPDRS III scores above/below 12)

B.7. Mortality

Mortality rates in the model are based on UK Life Tables for the general population, and adjustments for more advanced patients (defined through UPDRS III), based on data identified in the literature review (A.4).

B.7.1. Life tables

National Life Tables (United Kingdom 2012-2014) for general adults population in UK are presented in Table 91. Additionally, for illustrative purposes, estimated percentage of males and average death probability for the cohort of patients included in the base-case analysis (age = 52.5, proportion of males = 71.3%) is shown.

Table 91.
UK life tables for general adult population

Age	Death probability		Proportion of males in PD group	Average death probability
	Males	Females		
18	0.000443	0.000183	-	-
19	0.000477	0.000198	-	-
20	0.000467	0.000202	-	-
21	0.000473	0.000207	-	-
22	0.000468	0.000214	-	-
23	0.000555	0.000232	-	-
24	0.000521	0.000227	-	-
25	0.000559	0.000255	-	-
26	0.000641	0.000259	-	-
27	0.000620	0.000274	-	-
28	0.000627	0.000343	-	-
29	0.000709	0.000321	-	-
30	0.000755	0.000370	-	-
31	0.000793	0.000422	-	-
32	0.000796	0.000424	-	-
33	0.000875	0.000469	-	-
34	0.000924	0.000538	-	-
35	0.001016	0.000564	-	-
36	0.001047	0.000600	-	-
37	0.001176	0.000635	-	-
38	0.001355	0.000732	-	-
39	0.001420	0.000822	-	-

Age	Death probability		Proportion of males in PD group	Average death probability
	Males	Females		
40	0.001576	0.000883	-	-
41	0.001626	0.000957	-	-
42	0.001690	0.001058	-	-
43	0.001882	0.001156	-	-
44	0.002062	0.001270	-	-
45	0.002248	0.001382	-	-
46	0.002360	0.001446	-	-
47	0.002502	0.001622	-	-
48	0.002677	0.001710	-	-
49	0.002940	0.001924	-	-
50	0.003101	0.002156	-	-
51	0.003423	0.002344	-	-
52	0.003702	0.002558	71.3%	0.00337
53	0.004067	0.002780	71.3%	0.00370
54	0.004528	0.002977	71.3%	0.00408
55	0.004865	0.003402	71.2%	0.00444
56	0.005353	0.003674	71.2%	0.00487
57	0.005962	0.004033	71.2%	0.00541
58	0.006607	0.004385	71.1%	0.00597
59	0.007416	0.004772	71.1%	0.00665
60	0.008002	0.005226	71.0%	0.00720
61	0.008809	0.005808	71.0%	0.00794
62	0.009679	0.006283	70.9%	0.00869
63	0.010340	0.006755	70.8%	0.00929
64	0.011306	0.007356	70.8%	0.01015
65	0.012111	0.007936	70.7%	0.01089
66	0.013191	0.008579	70.6%	0.01183
67	0.014606	0.009639	70.5%	0.01314
68	0.016131	0.010748	70.4%	0.01454
69	0.017970	0.011719	70.3%	0.01611
70	0.019796	0.013122	70.1%	0.01780
71	0.022073	0.014429	70.0%	0.01978
72	0.025273	0.016475	69.8%	0.02262
73	0.027243	0.018281	69.6%	0.02452
74	0.029995	0.020211	69.5%	0.02701
75	0.033205	0.022532	69.2%	0.02992

Age	Death probability		Proportion of males in PD group	Average death probability
	Males	Females		
76	0.036573	0.025116	69.0%	0.03302
77	0.040211	0.028226	68.8%	0.03647
78	0.045461	0.031273	68.5%	0.04099
79	0.049611	0.035843	68.2%	0.04523
80	0.056322	0.040816	67.9%	0.05134
81	0.063280	0.045772	67.5%	0.05759
82	0.071519	0.051697	67.1%	0.06500
83	0.079828	0.058965	66.6%	0.07286
84	0.089056	0.067661	66.1%	0.08181
85	0.100248	0.076098	65.6%	0.09194
86	0.111772	0.085623	65.0%	0.10262
87	0.123954	0.096404	64.3%	0.11413
88	0.137712	0.106974	63.6%	0.12653
89	0.152512	0.122022	62.8%	0.14117
90	0.166455	0.136144	62.0%	0.15493
91	0.182981	0.151001	61.1%	0.17055
92	0.208161	0.171558	60.2%	0.19360
93	0.222733	0.185224	59.1%	0.20740
94	0.231918	0.202300	58.0%	0.21947
95	0.259055	0.219153	57.1%	0.24192
96	0.286001	0.251076	55.8%	0.27055
97	0.308416	0.267500	54.6%	0.28984
98	0.330830	0.289642	53.2%	0.31154
99	0.347717	0.315701	51.7%	0.33224
100	0.355920	0.329873	50.5%	0.34302

B.7.2. Association between UPDRS scores and mortality

According to the results of the meta-analysis by MacLeod 2014, mortality in PD is higher than in the general population. However, studies in early PD stages indicated mortality was not different from the general population (e.g. Peretz 2014, Williams-Gray 2013, Duarte 2013 [294, 307, 315]).

Five out of 22 identified studies in the systematic review (details in section A.4) reported data on the association between UPDRS score and mortality, of which 3 studies reported a correlation between UPDRS III scores and PD-related mortality (Marras 2005, Forsaa 2010 and Skorvanek 2013 [299–301]). Hazard ratios in these trials indicate a relation between a 10-point increase in UPDRS score

and mortality. Marras 2005 study also reported correlation between UPDRS II and UPDRS total score and mortality. Association between total UPDRS (defined as sum of UPDRS I, UPDRS II and UPDRS III) and mortality was also analysed in Vu 2012 . In Oosterveld 2014 study, UPDRS motor scores less than 30 were indicated as predictors of survival.

Based on available data, for predicting increase of mortality associated with disease progression measured by UPDRS, data on UPDRS III scores were used as this was the most frequently reported. Based on results of 3 studies, hazard ratio per 10 points change of UPDRS III scores was calculated – 1.31 (SD 0.094).

Table 92
Association between UPDRS III and mortality – data included in the analysis and model input data

Study	No of patients	Hazard Ratio per 10 points UPDRS III increase	Comment
**Marras 2005**	800	1.30	associations between baseline variables of interest and time to death
**Forsaa 2010**	230	1.25	associations between variables with mortality adjustment for age
**Skorvanek 2013**	153	1.48	only poster for this study was available
**Mean**	**-**	**1.31**	Weighted by number of patients
**SD**	**-**	**0.094**	**-**

As higher mortality in PD does not apply to patients at initial stages of disease, in the model the increased risk of mortality predicted by UPDRS scores was added for patients with UPDRS III score above 15 (value chosen arbitrarily).

REFERENCES

CSAI / CDLCI data

1. Fernandez HH, Standaert DG, Hauser RA, i in. Levodopa-carbidopa intestinal gel in advanced Parkinson's disease: Final 12-month, open-label results: Levodopa-Carbidopa Intestinal GEL in PD. *Movement Disorders*. 2015; 30(4):500–509.
2. Olanow CW, Kieburtz K, Odin P, i in. Continuous intrajejunal infusion of levodopa-carbidopa intestinal gel for patients with advanced Parkinson's disease: a randomised, controlled, double-blind, double-dummy study. *The Lancet Neurology*. 2014; 13(2):141–149.
3. Slevin JT, Fernandez HH, Zadikoff C, i in. Long-term safety and maintenance of efficacy of levodopa-carbidopa intestinal gel: an open-label extension of the double-blind pivotal study in advanced Parkinson's disease patients. *Journal of Parkinson's Disease*. 2015; 5(1):165–174.
4. Levodopa-Carbidopa Intestinal Gel Open-Label Study in Advanced Parkinson's Disease - Full Text View - ClinicalTrials.gov. https://clinicaltrials.gov/ct2/show/NCT00335153 (8.2.2016).
5. Study of Efficacy, Safety and Tolerability of Levodopa-Carbidopa Intestinal Gel in Levodopa-Responsive Parkinson's Subjects - Full Text View - ClinicalTrials.gov. https://clinicaltrials.gov/ct2/show/NCT00357994 (8.2.2016).
6. Study of Efficacy, Safety and Tolerability of Levodopa-Carbidopa Intestinal Gel in Levodopa-Responsive Parkinson's Subjects - Full Text View - ClinicalTrials.gov. https://clinicaltrials.gov/ct2/show/NCT00660387 (8.2.2016).
7. Elia AE, Dollenz C, Soliveri P, i in. Motor features and response to oral levodopa in patients with Parkinson's disease under continuous dopaminergic infusion or deep brain stimulation. *European Journal of Neurology: The Official Journal of the European Federation of Neurological Societies*. 2012; 19(1):76–83.
8. Martinez-Martin P, Reddy P, Katzenschlager R, i in. EuroInf: AMulticenter Comparative Observational Study of Apomorphine and Levodopa Infusion in Parkinson's Disease: Apomorphine and Levodopa in PD. *Movement Disorders*. 2015; 30(4):510–516.
9. Colzi A, Turner K, Lees AJ. Continuous subcutaneous waking day apomorphine in the long term treatment of levodopa induced interdose dyskinesias in Parkinson's disease. *Journal of Neurology, Neurosurgery, and Psychiatry*. 1998; 64(5):573–576.
10. De Gaspari D, Siri C, Landi A, i in. Clinical and neuropsychological follow up at 12 months in patients with complicated Parkinson's disease treated with subcutaneous apomorphine infusion or deep brain stimulation of the subthalamic nucleus. *Journal of Neurology, Neurosurgery, and Psychiatry*. 2006; 77(4):450–453.
11. Antonini A, Isaias IU, Rodolfi G, i in. A 5-year prospective assessment of advanced Parkinson disease patients treated with subcutaneous apomorphine infusion or deep brain stimulation. *Journal of Neurology*. 2011; 258(4):579–585.
12. Drapier S, Gillioz A-S, Leray E, i in. Apomorphine infusion in advanced Parkinson's patients with subthalamic stimulation contraindications. *Parkinsonism & Related Disorders*. 2012; 18(1):40–44.
13. García Ruiz PJ, Sesar Ignacio Á, Ares Pensado B, i in. Efficacy of long-term continuous subcutaneous apomorphine infusion in advanced Parkinson's disease with motor fluctuations: A multicenter study. *Movement Disorders*. 2008; 23(8):1130–1136.
14. Hughes AJ, Bishop S, Kleedorfer B, i in. Subcutaneous apomorphine in Parkinson's disease: response to chronic administration for up to five years. *Movement Disorders: Official Journal of the Movement Disorder Society*. 1993; 8(2):165–170.
15. Frankel JP, Lees AJ, Kempster PA, i in. Subcutaneous apomorphine in the treatment of Parkinson's disease. *Journal of Neurology, Neurosurgery, and Psychiatry*. 1990; 53(2):96–101.
16. Kanovský P, Kubová D, Bares M, i in. Levodopa-induced dyskinesias and continuous subcutaneous infusions of apomorphine: results of a two-year, prospective follow-up. *Movement Disorders: Official Journal of the Movement Disorder Society*. 2002; 17(1):188–191.
17. Katzenschlager R, Hughes A, Evans A, i in. Continuous subcutaneous apomorphine therapy improves dyskinesias in Parkinson's disease: A prospective study using single-dose challenges. *Movement Disorders*. 2005; 20(2):151–157.
18. Manson AJ, Turner K, Lees AJ. Apomorphine monotherapy in the treatment of refractory motor complications of Parkinson's disease: Long-term follow-up study of 64 patients. *Movement Disorders*. 2002; 17(6):1235–1241.

19. Pablo M-M, Prashanth R, Angelo A, i in. Chronic Subcutaneous Infusion Therapy with Apomorphine in Advanced Parkinson's Disease Compared to Conventional Therapy: A Real Life Study of Non Motor Effect. *Journal of Parkinson's Disease*. 2011; (2):197–203.
20. Morgante L, Basile G, Epifanio A, i in. Continuous apomorphine infusion (CAI) and neuropsychiatric disorders in patients with advanced Parkinson's disease: a follow-up of two years. *Archives of Gerontology and Geriatrics. Supplement*. 2004; (9):291–296.
21. Di Rosa AE, Epifanio A, Antonini A, i in. Continuous apomorphine infusion and neuropsychiatric disorders: a controlled study in patients with advanced Parkinson's disease. *Neurological Sciences*. 2003; 24(3):174–175.
22. Pietz K, Hagell P, Odin P. Subcutaneous apomorphine in late stage Parkinson's disease: a long term follow up. *Journal of Neurology, Neurosurgery, and Psychiatry*. 1998; 65(5):709–716.
23. Pinter MM, Helscher RJ, Mundsperger N, i in. Transient increase of pancreatic enzymes evoked by apomorphine in Parkinson's disease. *Journal of Neural Transmission (Vienna, Austria: 1996)*. 1998; 105(10-12):1237–1244.
24. Poewe W, Kleedorfer B, Wagner M, i in. Continuous subcutaneous apomorphine infusions for fluctuating Parkinson's disease. Long-term follow-up in 18 patients. *Advances in Neurology*. 1993; 60:656–659.
25. Wenning GK, Bösch S, Luginger E, i in. Effects of long-term, continuous subcutaneous apomorphine infusions on motor complications in advanced Parkinson's disease. *Advances in Neurology*. 1999; 80:545–548.
26. Stibe CM, Lees AJ, Kempster PA, i in. Subcutaneous apomorphine in parkinsonian on-off oscillations. *Lancet (London, England)*. 1988; 1(8582):403–406.
27. Stocchi F, Vacca L, De Pandis MF, i in. Subcutaneous continuous apomorphine infusion in fluctuating patients with Parkinson's disease: long-term results. *Neurological Sciences: Official Journal of the Italian Neurological Society and of the Italian Society of Clinical Neurophysiology*. 2001; 22(1):93–94.
28. Todorova A, Martinez-Martin P, Martin A, i in. Daytime apomorphine infusion combined with transdermal Rotigotine patch therapy is tolerated at 2 years: A 24-h treatment option in Parkinson's disease. *Basal Ganglia*. 2013; 3(2):127–130.
29. Antonini A, Mancini F, Canesi M, i in. Duodenal Levodopa Infusion Improves Quality of Life in Advanced Parkinson's Disease. *Neurodegenerative Diseases*. 2008; 5(3-4):244–246.
30. Antonini A, Odin P, opiano L, i in. Effect and safety of duodenal levodopa infusion in advanced Parkinson's disease: a retrospective multicenter outcome assessment in patient routine care. *Journal of Neural Transmission*. 2013; 120(11):1553–1558.
31. Antonini A, Yegin A, Preda C, i in. Global long-term study on motor and non-motor symptoms and safety of levodopa-carbidopa intestinal gel in routine care of advanced Parkinson's disease patients; 12-month interim outcomes. *Parkinsonism & Related Disorders*. 2015; 21(3):231–235.
32. Cáceres-Redondo MT, Carrillo F, Lama MJ, i in. Long-term levodopa/carbidopa intestinal gel in advanced Parkinson's disease. *Journal of Neurology*. 2014; 261(3):561–569.
33. Devos D, for the French DUODOPA Study Group. Patient profile, indications, efficacy and safety of duodenal levodopa infusion in advanced Parkinson's disease: Duodenal Levodopa Infusion in Parkinson's Disease. *Movement Disorders*. 2009; 24(7):993–1000.
34. Eggert K, Schrader C, Hahn M, i in. Continuous Jejunal Levodopa Infusion in Patients With Advanced Parkinson Disease: Practical Aspects and Outcome of Motor and Non-motor Complications. *Clinical Neuropharmacology*. 2008; 31(3):151–166.
35. Fasano A, Ricciardi L, Lena F, i in. Intrajejunal levodopa infusion in advanced Parkinson's disease: long-term effects on motor and non-motor symptoms and impact on patient's and caregiver's quality of life. *European Review for Medical and Pharmacological Sciences*. 2012; 16(1):79–89.
36. Fernandez HH, Vanagunas A, Odin P, i in. Levodopa–carbidopa intestinal gel in advanced Parkinson's disease open-label study: Interim results. *Parkinsonism & Related Disorders*. 2013; 19(3):339–345.
37. Standaert DG, Fernandez HH, Odin P, i in. A long-term, open-label study of levodopa–/INS;carbidopa intestinal gel in advanced Parkinson's disease patients: Functional and health-related quality-of-life endpoints. *Journal of the Neurological Sciences*. 2013; 333:e123–e124.
38. Foltynie T, Magee C, James C, i in. Impact of Duodopa on Quality of Life in Advanced Parkinson's Disease: A UK Case Series. *Parkinson's Disease*. 2013; 2013:1–5.
39. Honig H, Antonini A, Martinez-Martin P, i in. Intrajejunal levodopa infusion in Parkinson's disease: A pilot multicenter study of effects on nonmotor symptoms and quality of life. *Movement Disorders*. 2009; 24(10):1468–1474.
40. Isacson D, Bingefors K, Kristiansen IS, i in. Fluctuating functions related to quality of life in advanced Parkinson disease: effects of duodenal levodopa infusion. *Acta Neurologica Scandinavica*. 2008; 118(6):379–386.
41. Karlsborg M, Korbo L, Regeur L, i in. Duodopa pump treatment in patients with advanced Parkinson's disease. *Danish Medical Bulletin*. 2010; 57(6):A4155.

42. Lundqvist C, Beiske AG, Reiertsen O, i in. Real life cost and quality of life associated with continuous intraduodenal levodopa infusion compared with oral treatment in Parkinson patients. *Journal of Neurology*. 2014; 261(12):2438–2445.
43. Meppelink AM, Nyman R, van Laar T, i in. Transcutaneous port for continuous duodenal levodopa/carbidopa administration in Parkinson's disease. *Movement Disorders*. 2011; 26(2):331–334.
44. Merola A, Zibetti M, Angrisano S, i in. Comparison of subthalamic nucleus deep brain stimulation and Duodopa in the treatment of advanced Parkinson's disease. *Movement Disorders*. 2011; 26(4):664–670.
45. Nyholm D, Lewander T, Johansson A, i in. Enteral levodopa/carbidopa infusion in advanced Parkinson disease: long-term exposure. *Clinical Neuropharmacology*. 2008; 31(2):63–73.
46. Pålhagen SE, Dizdar N, Hauge T, i in. Interim analysis of long-term intraduodenal levodopa infusion in advanced Parkinson disease. *Acta Neurologica Scandinavica*. 2012; 126(6):e29–e33.
47. Pickut BA, van der Linden C, Dethy S, i in. Intestinal levodopa infusion: the Belgian experience. *Neurological Sciences*. 2013;
48. Sensi M, Preda F, Trevisani L, i in. Emerging issues on selection criteria of levodopa carbidopa infusion therapy: considerations on outcome of 28 consecutive patients. *Journal of Neural Transmission*. 2014; 121(6):633–642.
49. Zibetti M, Rizzone M, Merola A, i in. Sleep improvement with levodopa/carbidopa intestinal gel infusion in Parkinson disease. *Acta Neurologica Scandinavica*. 2013; 127(5):e28–e32.
50. Zibetti M, Merola A, Ricchi V, i in. Long-term duodenal levodopa infusion in Parkinson's disease: a 3-year motor and cognitive follow-up study. *Journal of Neurology*. 2013; 260(1):105–114.
51. Zibetti M, Merola A, Artusi CA, i in. Levodopa/carbidopa intestinal gel infusion in advanced Parkinson's disease: a 7-year experience. *European Journal of Neurology*. 2014; 21(2):312–318.

Long-term DBS effectiveness

52. Abboud H, Floden D, Thompson NR, i in. Impact of mild cognitive impairment on outcome following deep brain stimulation surgery for Parkinson's disease. *Parkinsonism & Related Disorders*. 2015; 21(3):249–253.
53. Accolla E, Caputo E, Cogiamanian F, i in. Gender differences in patients with Parkinson's disease treated with subthalamic deep brain stimulation. *Movement Disorders: Official Journal of the Movement Disorder Society*. 2007; 22(8):1150–1156.
54. Aviles-Olmos I, Kefalopoulou Z, Tripoliti E, i in. Long-term outcome of subthalamic nucleus deep brain stimulation for Parkinson's disease using an MRI-guided and MRI-verified approach. *Journal of Neurology, Neurosurgery, and Psychiatry*. 2014; 85(12):1419–1425.
55. Aybek S, Gronchi-Perrin A, Berney A, i in. Long-term cognitive profile and incidence of dementia after STN-DBS in Parkinson's disease. *Movement Disorders: Official Journal of the Movement Disorder Society*. 2007; 22(7):974–981.
56. Baba Y, Higuchi M-A, Abe H, i in. Anti-cholinergics for axial symptoms in Parkinson's disease after subthalamic stimulation. *Clinical Neurology and Neurosurgery*. 2012; 114(10):1308–1311.
57. Baizabal Carvallo JF, Mostile G, Almaguer M, i in. Deep brain stimulation hardware complications in patients with movement disorders: risk factors and clinical correlations. *Stereotactic and Functional Neurosurgery*. 2012; 90(5):300–306.
58. Bang Henriksen M, Johnsen EL, Sunde N, i in. Surviving 10 years with deep brain stimulation for Parkinson's disease - a follow-up of 79 patients. *European Journal of Neurology: The Official Journal of the European Federation of Neurological Societies*. 2014;
59. Bannier S, Montaurier C, Derost PP, i in. Overweight after deep brain stimulation of the subthalamic nucleus in Parkinson disease: long term follow-up. *Journal of Neurology, Neurosurgery, and Psychiatry*. 2009; 80(5):484–488.
60. Barichella M, Marczewska AM, Mariani C, i in. Body weight gain rate in patients with Parkinson's disease and deep brain stimulation. *Movement Disorders: Official Journal of the Movement Disorder Society*. 2003; 18(11):1337–1340.
61. Boviatsis EJ, Stavrinou LC, Themistocleous M, i in. Surgical and hardware complications of deep brain stimulation. A seven-year experience and review of the literature. *Acta Neurochirurgica*. 2010; 152(12):2053–2062.
62. Burdick AP, Fernandez HH, Okun MS, i in. Relationship between higher rates of adverse events in deep brain stimulation using standardized prospective recording and patient outcomes. *Neurosurgical Focus*. 2010; 29(2):E4.
63. Capecci M, Ricciuti RA, Burini D, i in. Functional improvement after subthalamic stimulation in Parkinson's disease: a non-equivalent controlled study with 12-24 month follow up. *Journal of Neurology, Neurosurgery, and Psychiatry*. 2005; 76(6):769–774.

64. Castelli L, Rizzi L, Zibetti M, i in. Neuropsychological changes 1-year after subthalamic DBS in PD patients: A prospective controlled study. *Parkinsonism & Related Disorders*. 2010; 16(2):115–118.
65. Castrioto A, Kistner A, Klinger H, i in. Psychostimulant effect of levodopa: reversing sensitisation is possible. *Journal of Neurology, Neurosurgery, and Psychiatry*. 2013; 84(1):18–22.
66. Castrioto A, Funkiewiez A, Debû B, i in. Iowa gambling task impairment in Parkinson's disease can be normalised by reduction of dopaminergic medication after subthalamic stimulation. *Journal of Neurology, Neurosurgery, and Psychiatry*. 2015; 86(2):186–190.
67. Chandran S, Krishnan S, Rao RM, i in. Gender influence on selection and outcome of deep brain stimulation for Parkinson's disease. *Annals of Indian Academy of Neurology*. 2014; 17(1):66–70.
68. Chiou S-M, Lin Y-C, Lu M-K, i in. Bilateral subthalamic stimulation for advanced Parkinson disease: early experience at an Eastern center. *Neurological Sciences: Official Journal of the Italian Neurological Society and of the Italian Society of Clinical Neurophysiology*. 2015; 36(4):515–520.
69. Cilia R, Siri C, Marotta G, i in. Brain networks underlining verbal fluency decline during STN-DBS in Parkinson's disease: an ECD-SPECT study. *Parkinsonism & Related Disorders*. 2007; 13(5):290–294.
70. Cilia R, Marotta G, Landi A, i in. Clinical and cerebral activity changes induced by subthalamic nucleus stimulation in advanced Parkinson's disease: a prospective case-control study. *Clinical Neurology and Neurosurgery*. 2009; 111(2):140–146.
71. Cohen DB, Oh MY, Baser SM, i in. Fast-track programming and rehabilitation model: a novel approach to postoperative deep brain stimulation patient care. *Archives of Physical Medicine and Rehabilitation*. 2007; 88(10):1320–1324.
72. Cury RG, Galhardoni R, Fonoff ET, i in. Effects of deep brain stimulation on pain and other nonmotor symptoms in Parkinson disease. *Neurology*. 2014; 83(16):1403–1409.
73. Daniele A, Albanese A, Contarino MF, i in. Cognitive and behavioural effects of chronic stimulation of the subthalamic nucleus in patients with Parkinson's disease. *Journal of Neurology, Neurosurgery, and Psychiatry*. 2003; 74(2):175–182.
74. Daniels C, Krack P, Volkmann J, i in. Is improvement in the quality of life after subthalamic nucleus stimulation in Parkinson's disease predictable? *Movement Disorders: Official Journal of the Movement Disorder Society*. 2011; 26(14):2516–2521.
75. Davis JT, Lyons KE, Pahwa R. Freezing of gait after bilateral subthalamic nucleus stimulation for Parkinson's disease. *Clinical Neurology and Neurosurgery*. 2006; 108(5):461–464.
76. Deep-Brain Stimulation for Parkinson's Disease Study Group. Deep-brain stimulation of the subthalamic nucleus or the pars interna of the globus pallidus in Parkinson's disease. *The New England Journal of Medicine*. 2001; 345(13):956–963.
77. DeLong MR, Huang KT, Gallis J, i in. Effect of advancing age on outcomes of deep brain stimulation for Parkinson disease. *JAMA neurology*. 2014; 71(10):1290–1295.
78. Derost P-P, Ouchchane L, Morand D, i in. Is DBS-STN appropriate to treat severe Parkinson disease in an elderly population? *Neurology*. 2007; 68(17):1345–1355.
79. Derrey S, Maltête D, Chastan N, i in. Deep brain stimulation of the subthalamic nucleus in Parkinson's disease: usefulness of intraoperative radiological guidance. The Stereoplan. *Stereotactic and Functional Neurosurgery*. 2008; 86(6):351–358.
80. Derrey S, Lefaucheur R, Chastan N, i in. Alleviation of off-period dystonia in Parkinson disease by a microlesion following subthalamic implantation. *Journal of Neurosurgery*. 2010; 112(6):1263–1266.
81. Deuschl G, Schade-Brittinger C, Krack P, i in. A randomized trial of deep-brain stimulation for Parkinson's disease. *The New England Journal of Medicine*. 2006; 355(9):896–908.
82. Doshi PK, Chhaya NA, Bhatt MA. Bilateral subthalamic nucleus stimulation for Parkinson's disease. *Neurology India*. 2003; 51(1):43–48.
83. Drapier S, Raoul S, Drapier D, i in. Only physical aspects of quality of life are significantly improved by bilateral subthalamic stimulation in Parkinson's disease. *Journal of Neurology*. 2005; 252(5):583–588.
84. Erola T, Karinen P, Heikkinen E, i in. Bilateral subthalamic nucleus stimulation improves health-related quality of life in Parkinsonian patients. *Parkinsonism & Related Disorders*. 2005; 11(2):89–94.
85. Erola T, Heikkinen ER, Haapaniemi T, i in. Efficacy of bilateral subthalamic nucleus (STN) stimulation in Parkinson's disease. *Acta Neurochirurgica*. 2006; 148(4):389–394.
86. Esselink R a. J, de Bie RMA, de Haan RJ, i in. Unilateral pallidotomy versus bilateral subthalamic nucleus stimulation in PD: a randomized trial. *Neurology*. 2004; 62(2):201–207.
87. Esselink R a. J, de Bie RMA, de Haan RJ, i in. Long-term superiority of subthalamic nucleus stimulation over pallidotomy in Parkinson disease. *Neurology*. 2009; 73(2):151–153.
88. Eusebio A, Witjas T, Cohen J, i in. Subthalamic nucleus stimulation and compulsive use of dopaminergic medication in Parkinson's disease. *Journal of Neurology, Neurosurgery, and Psychiatry*. 2013; 84(8):868–874.

89. Evidente VGH, Premkumar AP, Adler CH, i in. Medication dose reductions after pallidal versus subthalamic stimulation in patients with Parkinson's disease. *Acta Neurologica Scandinavica*. 2011; 124(3):211–214.
90. Falowski S, Ooi YC, Smith A, i in. An evaluation of hardware and surgical complications with deep brain stimulation based on diagnosis and lead location. *Stereotactic and Functional Neurosurgery*. 2012; 90(3):173–180.
91. Fasano A, Romito LM, Daniele A, i in. Motor and cognitive outcome in patients with Parkinson's disease 8 years after subthalamic implants. *Brain*. 2010; 133(9):2664–2676.
92. Fenoy AJ, Simpson RK. Risks of common complications in deep brain stimulation surgery: management and avoidance. *Journal of Neurosurgery*. 2014; 120(1):132–139.
93. Ferraye MU, Debû B, Fraix V, i in. Effects of subthalamic nucleus stimulation and levodopa on freezing of gait in Parkinson disease. *Neurology*. 2008; 70(16 Pt 2):1431–1437.
94. Ferraye MU, Ardouin C, Lhommée E, i in. Levodopa-resistant freezing of gait and executive dysfunction in Parkinson's disease. *European Neurology*. 2013; 69(5):281–288.
95. Figueiras-Méndez R, Regidor I, Riva-Meana C, i in. Further supporting evidence of beneficial subthalamic stimulation in Parkinson's patients. *Neurology*. 2002; 58(3):469–470.
96. Fluchere F, Witjas T, Eusebio A, i in. Controlled general anaesthesia for subthalamic nucleus stimulation in Parkinson's disease. *Journal of Neurology, Neurosurgery, and Psychiatry*. 2014; 85(10):1167–1173.
97. Follett KA, Weaver FM, Stern M, i in. Pallidal versus subthalamic deep-brain stimulation for Parkinson's disease. *The New England Journal of Medicine*. 2010; 362(22):2077–2091.
98. Ford B, Winfield L, Pullman SL, i in. Subthalamic nucleus stimulation in advanced Parkinson's disease: blinded assessments at one year follow up. *Journal of Neurology, Neurosurgery, and Psychiatry*. 2004; 75(9):1255–1259.
99. Foubert-Samier A, Maurice S, Hivert S, i in. A long-term follow-up of weight changes in subthalamic nucleus stimulated Parkinson's disease patients. *Revue Neurologique*. 2012; 168(2):173–176.
100. Fraix V, Houeto J-L, Lagrange C, i in. Clinical and economic results of bilateral subthalamic nucleus stimulation in Parkinson's disease. *Journal of Neurology, Neurosurgery, and Psychiatry*. 2006; 77(4):443–449.
101. Funkiewiez A, Ardouin C, Cools R, i in. Effects of levodopa and subthalamic nucleus stimulation on cognitive and affective functioning in Parkinson's disease. *Movement Disorders: Official Journal of the Movement Disorder Society*. 2006; 21(10):1656–1662.
102. Gan J, Xie-Brustolin J, Mertens P, i in. Bilateral subthalamic nucleus stimulation in advanced Parkinson's disease: three years follow-up. *Journal of Neurology*. 2007; 254(1):99–106.
103. St George RJ, Carlson-Kuhta P, Burchiel KJ, i in. The effects of subthalamic and pallidal deep brain stimulation on postural responses in patients with Parkinson disease. *Journal of Neurosurgery*. 2012; 116(6):1347–1356.
104. Gervais-Bernard H, Xie-Brustolin J, Mertens P, i in. Bilateral subthalamic nucleus stimulation in advanced Parkinson's disease: five year follow-up. *Journal of Neurology*. 2009; 256(2):225–233.
105. Gómez-Esteban JC, Lezcano E, Zarranz JJ, i in. Outcome of bilateral deep brain subthalamic stimulation in patients carrying the R1441G mutation in the LRRK2 dardarin gene. *Neurosurgery*. 2008; 62(4):857–862; discussion 862–863.
106. Guehl D, Cuny E, Benazzouz A, i in. Side-effects of subthalamic stimulation in Parkinson's disease: clinical evolution and predictive factors. *European Journal of Neurology: The Official Journal of the European Federation of Neurological Societies*. 2006; 13(9):963–971.
107. Guo S, Zhuang P, Hallett M, i in. Subthalamic deep brain stimulation for Parkinson's disease: correlation between locations of oscillatory activity and optimal site of stimulation. *Parkinsonism & Related Disorders*. 2013; 19(1):109–114.
108. Hamasaki T, Yamada K, Hirai T, i in. A positive correlation between fractional white matter volume and the response of Parkinson disease patients to subthalamic stimulation. *Acta Neurochirurgica*. 2010; 152(6):997–1006; discussion 1006.
109. Hamel W, Fietzek U, Morsnowski A, i in. Deep brain stimulation of the subthalamic nucleus in Parkinson's disease: evaluation of active electrode contacts. *Journal of Neurology, Neurosurgery, and Psychiatry*. 2003; 74(8):1036–1046.
110. Hariz MI, Rehncrona S, Quinn NP, i in. Multicenter study on deep brain stimulation in Parkinson's disease: an independent assessment of reported adverse events at 4 years. *Movement Disorders: Official Journal of the Movement Disorder Society*. 2008; 23(3):416–421.
111. Harries AM, Kausar J, Roberts SAG, i in. Deep brain stimulation of the subthalamic nucleus for advanced Parkinson disease using general anesthesia: long-term results. *Journal of Neurosurgery*. 2012; 116(1):107–113.
112. Herzog J, Volkmann J, Krack P, i in. Two-year follow-up of subthalamic deep brain stimulation in Parkinson's disease. *Movement Disorders: Official Journal of the Movement Disorder Society*. 2003; 18(11):1332–1337.

113. Herzog J, Möller B, Witt K, i in. Influence of subthalamic deep brain stimulation versus levodopa on motor perseverations in Parkinson's disease. *Movement Disorders: Official Journal of the Movement Disorder Society*. 2009; 24(8):1206–1210.
114. Houeto JL, Damier P, Bejjani PB, i in. Subthalamic stimulation in Parkinson disease: a multidisciplinary approach. *Archives of Neurology*. 2000; 57(4):461–465.
115. Houeto J-L, Mallet L, Mesnage V, i in. Subthalamic stimulation in Parkinson disease: behavior and social adaptation. *Archives of Neurology*. 2006; 63(8):1090–1095.
116. Hung H-Y, Tsai S-T, Lin S-H, i in. Uneven benefits of subthalamic nucleus deep brain stimulation in Parkinson's disease—A 7-year cross-sectional study. *Tzu Chi Medical Journal*. 2013; 25(4):239–245.
117. Jaggi JL, Umemura A, Hurtig HI, i in. Bilateral stimulation of the subthalamic nucleus in Parkinson's disease: surgical efficacy and prediction of outcome. *Stereotactic and Functional Neurosurgery*. 2004; 82(2-3):104–114.
118. Janssen MLF, Duits AA, Turaihi AM, i in. Subthalamic nucleus high-frequency stimulation for advanced Parkinson's disease: motor and neuropsychological outcome after 10 years. *Stereotactic and Functional Neurosurgery*. 2014; 92(6):381–387.
119. Jiang J-L, Chen S-Y, Hsieh T-C, i in. Different effectiveness of subthalamic deep brain stimulation in Parkinson's disease: A comparative cohort study at 1 year and 5 years. *Journal of the Formosan Medical Association = Taiwan Yi Zhi*. 2013;
120. Kalteis K, Standhardt H, Kryspin-Exner I, i in. Influence of bilateral Stn-stimulation on psychiatric symptoms and psychosocial functioning in patients with Parkinson's disease. *Journal of Neural Transmission (Vienna, Austria: 1996)*. 2006; 113(9):1191–1206.
121. Kenney C, Simpson R, Hunter C, i in. Short-term and long-term safety of deep brain stimulation in the treatment of movement disorders. *Journal of Neurosurgery*. 2007; 106(4):621–625.
122. Kim H-J, Jeon BS, Lee J-Y, i in. The benefit of subthalamic deep brain stimulation for pain in Parkinson disease: a 2-year follow-up study. *Neurosurgery*. 2012; 70(1):18–23; discussion 23–24.
123. Kim HY, Chang WS, Kang DW, i in. Factors related to outcomes of subthalamic deep brain stimulation in Parkinson's disease. *Journal of Korean Neurosurgical Society*. 2013; 54(2):118–124.
124. Kim YE, Jeon BS, Paek S-H, i in. Rapid eye movement sleep behavior disorder after bilateral subthalamic stimulation in Parkinson's disease. *Journal of Clinical Neuroscience: Official Journal of the Neurosurgical Society of Australasia*. 2015; 22(2):315–319.
125. Kishore A, Rao R, Krishnan S, i in. Long-term stability of effects of subthalamic stimulation in Parkinson's disease: Indian Experience. *Movement Disorders: Official Journal of the Movement Disorder Society*. 2010; 25(14):2438–2444.
126. Kleiner-Fisman G, Fisman DN, Sime E, i in. Long-term follow up of bilateral deep brain stimulation of the subthalamic nucleus in patients with advanced Parkinson disease. *Journal of Neurosurgery*. 2003; 99(3):489–495.
127. Koike Y, Shima F, Nakamizo A, i in. Direct localization of subthalamic nucleus supplemented by single-track electrophysiological guidance in deep brain stimulation lead implantation: techniques and clinical results. *Stereotactic and Functional Neurosurgery*. 2008; 86(3):173–178.
128. Krack P, Batir A, Van Blercom N, i in. Five-year follow-up of bilateral stimulation of the subthalamic nucleus in advanced Parkinson's disease. *The New England Journal of Medicine*. 2003; 349(20):1925–1934.
129. Krause M, Fogel W, Mayer P, i in. Chronic inhibition of the subthalamic nucleus in Parkinson's disease. *Journal of the Neurological Sciences*. 2004; 219(1-2):119–124.
130. Kumar R, Lang AE, Rodriguez-Oroz MC, i in. Deep brain stimulation of the globus pallidus pars interna in advanced Parkinson's disease. *Neurology*. 2000; 55(12 Suppl 6):S34–39.
131. Lagrange E, Krack P, Moro E, i in. Bilateral subthalamic nucleus stimulation improves health-related quality of life in PD. *Neurology*. 2002; 59(12):1976–1978.
132. Langner-Lemercier S, Drapier S, Naudet F, i in. Preoperative brain metabolism and quality of life after subthalamic nucleus stimulation in Parkinson's disease. *Journal of Neurology*. 2015; 262(4):881–889.
133. Lee JY, Han JH, Kim HJ, i in. STN DBS of Advanced Parkinson's Disease Experienced in a Specialized Monitoring Unit with a Prospective Protocol. *Journal of Korean Neurosurgical Society*. 2008; 44(1):26–35.
134. Lefaucheur J-P, Gurruchaga J-M, Pollin B, i in. Outcome of bilateral subthalamic nucleus stimulation in the treatment of Parkinson's disease: correlation with intra-operative multi-unit recordings but not with the type of anaesthesia. *European Neurology*. 2008; 60(4):186–199.
135. Lewis CJ, Maier F, Eggers C, i in. Parkinson's disease patients with subthalamic stimulation and carers judge quality of life differently. *Parkinsonism & Related Disorders*. 2014; 20(5):514–519.
136. Lewis CJ, Maier F, Horstkötter N, i in. Subjectively perceived personality and mood changes associated with subthalamic stimulation in patients with Parkinson's disease. *Psychological Medicine*. 2015; 45(1):73–85.
137. Lhommée E, Klinger H, Thobois S, i in. Subthalamic stimulation in Parkinson's disease: restoring the balance of motivated behaviours. *Brain: A Journal of Neurology*. 2012; 135(Pt 5):1463–1477.

138. Li D, Cao C, Zhang J, i in. Subthalamic nucleus deep brain stimulation for Parkinson's disease: 8 years of follow-up. *Translational Neurodegeneration*. 2013; 2(1):11.

139. Liang GS, Chou KL, Baltuch GH, i in. Long-term outcomes of bilateral subthalamic nucleus stimulation in patients with advanced Parkinson's disease. *Stereotactic and Functional Neurosurgery*. 2006; 84(5-6):221–227.

140. Liang J, Hu X, Zhou X, i in. Five-year follow-up of 23 asymmetrical Parkinson's disease patients treated with unilateral subthalamic nucleus stimulation. *Neural Regeneration Research*. 2012; 7(18):1428–1435.

141. Lilleeng B, Gjerstad M, Baardsen R, i in. Motor symptoms after deep brain stimulation of the subthalamic nucleus. *Acta Neurologica Scandinavica*. 2015; 131(5):298–304.

142. Limousin P, Krack P, Pollak P, i in. Electrical stimulation of the subthalamic nucleus in advanced Parkinson's disease. *The New England Journal of Medicine*. 1998; 339(16):1105–1111.

143. Liu H, Ma Y, Zhang K, i in. Subthalamic deep brain stimulation with a new device in Parkinson's disease: an open-label trial. *Neuromodulation: Journal of the International Neuromodulation Society*. 2013; 16(3):212–218; discussion 218.

144. Locke MC, Wu SS, Foote KD, i in. Weight changes in subthalamic nucleus vs globus pallidus internus deep brain stimulation: results from the COMPARE Parkinson disease deep brain stimulation cohort. *Neurosurgery*. 2011; 68(5):1233–1237; discussion 1237–1238.

145. Lokkegaard A, Werdelin LM, Regeur L, i in. Dopamine transporter imaging and the effects of deep brain stimulation in patients with Parkinson's disease. *European Journal of Nuclear Medicine and Molecular Imaging*. 2007; 34(4):508–516.

146. Lyons KE, Wilkinson SB, Overman J, i in. Surgical and hardware complications of subthalamic stimulation: a series of 160 procedures. *Neurology*. 2004; 63(4):612–616.

147. Lyons KE, Pahwa R. Effects of bilateral subthalamic nucleus stimulation on sleep, daytime sleepiness, and early morning dystonia in patients with Parkinson disease. *Journal of Neurosurgery*. 2006; 104(4):502–505.

148. Merola A, Zibetti M, Artusi CA, i in. Subthalamic nucleus deep brain stimulation outcome in young onset Parkinson's disease: a role for age at disease onset? *Journal of Neurology, Neurosurgery, and Psychiatry*. 2012; 83(3):251–257.

149. Merola A, Rizzi L, Artusi CA, i in. Subthalamic deep brain stimulation: clinical and neuropsychological outcomes in mild cognitive impaired parkinsonian patients. *Journal of Neurology*. 2014; 261(9):1745–1751.

150. Minguez-Castellanos A, Escamilla-Sevilla F, Katati MJ, i in. Different patterns of medication change after subthalamic or pallidal stimulation for Parkinson's disease: target related effect or selection bias? *Journal of Neurology, Neurosurgery, and Psychiatry*. 2005; 76(1):34–39.

151. Monteiro A, Andrade C, Rosas MJ, i in. Deep brain stimulation of the subthalamic nucleus in advanced Parkinson's disease: five year follow-up at a Portuguese center. *Revista De Neurologia*. 2014; 58(10):433–439.

152. Montel SR, Bungener C. Coping and quality of life of patients with Parkinson disease who have undergone deep brain stimulation of the subthalamic nucleus. *Surgical Neurology*. 2009; 72(2):105–110; discussion 110–111.

153. Nazzaro JM, Pahwa R, Lyons KE. The impact of bilateral subthalamic stimulation on non-motor symptoms of Parkinson's disease. *Parkinsonism & Related Disorders*. 2011; 17(8):606–609.

154. Ngoga D, Mitchell R, Kausar J, i in. Deep brain stimulation improves survival in severe Parkinson's disease. *Journal of Neurology, Neurosurgery, and Psychiatry*. 2014; 85(1):17–22.

155. Nilsson MH, Törnqvist AL, Rehncrona S. Deep-brain stimulation in the subthalamic nuclei improves balance performance in patients with Parkinson's disease, when tested without anti-parkinsonian medication. *Acta Neurologica Scandinavica*. 2005; 111(5):301–308.

156. Nunta-Aree S, Sitthinamsuwan B, Boonyapisit K, i in. SW2-year outcomes of subthalamic deep brain stimulation for idiopathic Parkinson's disease. *Journal of the Medical Association of Thailand = Chotmaihet Thangphaet*. 2010; 93(5):529–540.

157. Odekerken VJJ, van Laar T, Staal MJ, i in. Subthalamic nucleus versus globus pallidus bilateral deep brain stimulation for advanced Parkinson's disease (NSTAPS study): a randomised controlled trial. *The Lancet. Neurology*. 2013; 12(1):37–44.

158. Ogura M, Nakao N, Nakai E, i in. The mechanism and effect of chronic electrical stimulation of the globus pallidus for treatment of Parkinson disease. *Journal of Neurosurgery*. 2004; 100(6):997–1001.

159. Okun MS, Gallo BV, Mandybur G, i in. Subthalamic deep brain stimulation with a constant-current device in Parkinson's disease: an open-label randomised controlled trial. *The Lancet. Neurology*. 2012; 11(2):140–149.

160. Okun MS, Wu SS, Fayad S, i in. Acute and Chronic Mood and Apathy Outcomes from a randomized study of unilateral STN and GPi DBS. *PloS One*. 2014; 9(12):e114140.

161. Ondo WG, Silay Y, Almaguer M, i in. Subthalamic deep brain stimulation in patients with a previous pallidotomy. *Movement Disorders: Official Journal of the Movement Disorder Society*. 2006; 21(8):1252–1254.

162. Ory-Magne F, Brefel-Courbon C, Simonetta-Moreau M, i in. Does ageing influence deep brain stimulation outcomes in Parkinson's disease? *Movement Disorders: Official Journal of the Movement Disorder Society*. 2007; 22(10):1457–1463.

163. Østergaard K, Sunde N, Dupont E. Effects of bilateral stimulation of the subthalamic nucleus in patients with severe Parkinson's disease and motor fluctuations. *Movement Disorders: Official Journal of the Movement Disorder Society*. 2002; 17(4):693–700.

164. Østergaard K, Aa Sunde N. Evolution of Parkinson's disease during 4 years of bilateral deep brain stimulation of the subthalamic nucleus. *Movement Disorders: Official Journal of the Movement Disorder Society*. 2006; 21(5):624–631.

165. Otaka T, Oshima H, Katayama Y, i in. Impact of subthalamic nucleus stimulation on young-onset Parkinson's disease. *Neuromodulation: Journal of the International Neuromodulation Society*. 2010; 13(1):10–16.

166. Oyama G, Foote KD, Jacobson CE, i in. GPi and STN deep brain stimulation can suppress dyskinesia in Parkinson's disease. *Parkinsonism & Related Disorders*. 2012; 18(7):814–818.

167. Paek SH, Lee J-Y, Kim H-J, i in. Electrode position and the clinical outcome after bilateral subthalamic nucleus stimulation. *Journal of Korean Medical Science*. 2011; 26(10):1344–1355.

168. Paek SH, Kim HJ, Yoon JY, i in. Fusion image-based programming after subthalamic nucleus deep brain stimulation. *World Neurosurgery*. 2011; 75(3-4):517–524.

169. Paek SH, Yun JY, Song SW, i in. The clinical impact of precise electrode positioning in STN DBS on three-year outcomes. *Journal of the Neurological Sciences*. 2013; 327(1-2):25–31.

170. Paluzzi A, Belli A, Bain P, i in. Operative and hardware complications of deep brain stimulation for movement disorders. *British Journal of Neurosurgery*. 2006; 20(5):290–295.

171. Patel DM, Walker HC, Brooks R, i in. Adverse events associated with deep brain stimulation for movement disorders: analysis of 510 consecutive cases. *Neurosurgery*. 2015; 11 Suppl 2:190–199.

172. Pellaprat J, Ory-Magne F, Canivet C, i in. Deep brain stimulation of the subthalamic nucleus improves pain in Parkinson's disease. *Parkinsonism & Related Disorders*. 2014; 20(6):662–664.

173. Perriol M-P, Krystkowiak P, Defebvre L, i in. Stimulation of the subthalamic nucleus in Parkinson's disease: cognitive and affective changes are not linked to the motor outcome. *Parkinsonism & Related Disorders*. 2006; 12(4):205–210.

174. Piboolnurak P, Lang AE, Lozano AM, i in. Levodopa response in long-term bilateral subthalamic stimulation for Parkinson's disease. *Movement Disorders: Official Journal of the Movement Disorder Society*. 2007; 22(7):990–997.

175. Portman AT, van Laar T, Staal MJ, i in. Chronic stimulation of the subthalamic nucleus increases daily on-time without dyskinesia in advanced Parkinson's disease. *Parkinsonism & Related Disorders*. 2006; 12(3):143–148.

176. Price CC, Favilla C, Tanner JJ, i in. Lateral ventricle volume is poor predictor of post unilateral DBS motor change for Parkinson's disease. *Parkinsonism & Related Disorders*. 2011; 17(5):343–347.

177. Reese R, Pinsker MO, Herzog J, i in. The atypical subthalamic nucleus--an anatomical variant relevant for stereotactic targeting. *Movement Disorders: Official Journal of the Movement Disorder Society*. 2012; 27(4):544–548.

178. Rizzone MG, Fasano A, Daniele A, i in. Long-term outcome of subthalamic nucleus DBS in Parkinson's disease: from the advanced phase towards the late stage of the disease? *Parkinsonism & Related Disorders*. 2014; 20(4):376–381.

179. Rocha S, Monteiro A, Linhares P, i in. Long-term mortality analysis in Parkinson's disease treated with deep brain stimulation. *Parkinson's Disease*. 2014; 2014:717041.

180. Rodriguez-Oroz MC, Obeso JA, Lang AE, i in. Bilateral deep brain stimulation in Parkinson's disease: a multicentre study with 4 years follow-up. *Brain: A Journal of Neurology*. 2005; 128(Pt 10):2240–2249.

181. Romito LMA, Scerrati M, Contarino MF, i in. Long-term follow up of subthalamic nucleus stimulation in Parkinson's disease. *Neurology*. 2002; 58(10):1546–1550.

182. Romito LM, Scerrati M, Contarino MF, i in. Bilateral high frequency subthalamic stimulation in Parkinson's disease: long-term neurological follow-up. *Journal of Neurosurgical Sciences*. 2003; 47(3):119–128.

183. Romito LM, Contarino MF, Vanacore N, i in. Replacement of dopaminergic medication with subthalamic nucleus stimulation in Parkinson's disease: long-term observation. *Movement Disorders: Official Journal of the Movement Disorder Society*. 2009; 24(4):557–563.

184. Schlaier JR, Hanson C, Janzen A, i in. Deep brain stimulation in Parkinson's disease: motor effects relative to the MRI-defined STN. *Neurosurgical Review*. 2014; 37(3):461–470; discussion 470–471.

185. Schüpbach WMM, Chastan N, Welter ML, i in. Stimulation of the subthalamic nucleus in Parkinson's disease: a 5 year follow up. *Journal of Neurology, Neurosurgery, and Psychiatry*. 2005; 76(12):1640–1644.

186. Schüpbach MWM, Welter ML, Bonnet AM, i in. Mortality in patients with Parkinson's disease treated by stimulation of the subthalamic nucleus. *Movement Disorders: Official Journal of the Movement Disorder Society*. 2007; 22(2):257–261.

187. Seijo FJ, Alvarez-Vega MA, Gutierrez JC, i in. Complications in subthalamic nucleus stimulation surgery for treatment of Parkinson's disease. Review of 272 procedures. *Acta Neurochirurgica*. 2007; 149(9):867–875; discussion 876.

188. Seijo F, Alvarez de Eulate Beramendi S, Santamarta Liébana E, i in. Surgical adverse events of deep brain stimulation in the subthalamic nucleus of patients with Parkinson's disease. The learning curve and the pitfalls. *Acta Neurochirurgica*. 2014; 156(8):1505–1512; discussion 1512.

189. Simonin C, Tir M, Devos D, i in. Reduced levodopa-induced complications after 5 years of subthalamic stimulation in Parkinson's disease: a second honeymoon. *Journal of Neurology*. 2009; 256(10):1736–1741.

190. Smeding HMM, Speelman JD, Koning-Haanstra M, i in. Neuropsychological effects of bilateral STN stimulation in Parkinson disease: a controlled study. *Neurology*. 2006; 66(12):1830–1836.

191. Smeding HMM, Speelman JD, Huizenga HM, i in. Predictors of cognitive and psychosocial outcome after STN DBS in Parkinson's Disease. *Journal of Neurology, Neurosurgery, and Psychiatry*. 2011; 82(7):754–760.

192. Soulas T, Sultan S, Gurruchaga J-M, i in. Depression and coping as predictors of change after deep brain stimulation in Parkinson's disease. *World Neurosurgery*. 2011; 75(3-4):525–532.

193. Soulas T, Sultan S, Gurruchaga J-M, i in. Changes in quality of life, burden and mood among spouses of Parkinson's disease patients receiving neurostimulation. *Parkinsonism & Related Disorders*. 2012; 18(5):602–605.

194. Sung VW, Watts RL, Schrandt CJ, i in. The relationship between clinical phenotype and early staged bilateral deep brain stimulation in Parkinson disease. *Journal of Neurosurgery*. 2013; 119(6):1530–1536.

195. Tagliati M, Martin C, Alterman R. Lack of motor symptoms progression in Parkinson's disease patients with long-term bilateral subthalamic deep brain stimulation. *The International Journal of Neuroscience*. 2010; 120(11):717–723.

196. Tai C-H, Wu R-M, Lin C-H, i in. Deep brain stimulation therapy for Parkinson's disease using frameless stereotaxy: comparison with frame-based surgery. *European Journal of Neurology: The Official Journal of the European Federation of Neurological Societies*. 2010; 17(11):1377–1385.

197. Tanei T, Kajita Y, Kaneoke Y, i in. Staged bilateral deep brain stimulation of the subthalamic nucleus for the treatment of Parkinson's disease. *Acta Neurochirurgica*. 2009; 151(6):589–594.

198. Tang V, Zhu CXL, Chan D, i in. Evidence of improved immediate verbal memory and diminished category fluency following STN-DBS in Chinese-Cantonese patients with idiopathic Parkinson's disease. *Neurological Sciences: Official Journal of the Italian Neurological Society and of the Italian Society of Clinical Neurophysiology*. 2015; 36(8):1371–1377.

199. Tavella A, Bergamasco B, Bosticco E, i in. Deep brain stimulation of the subthalamic nucleus in Parkinson's disease: long-term follow-up. *Neurological Sciences: Official Journal of the Italian Neurological Society and of the Italian Society of Clinical Neurophysiology*. 2002; 23 Suppl 2:S111–112.

200. Temel Y, Wilbrink P, Duits A, i in. Single electrode and multiple electrode guided electrical stimulation of the subthalamic nucleus in advanced Parkinson's disease. *Neurosurgery*. 2007; 61(5 Suppl 2):346–355; discussion 355–357.

201. Tir M, Devos D, Blond S, i in. Exhaustive, one-year follow-up of subthalamic nucleus deep brain stimulation in a large, single-center cohort of parkinsonian patients. *Neurosurgery*. 2007; 61(2):297–304; discussion 304–305.

202. Toft M, Lilleeng B, Ramm-Pettersen J, i in. Long-term efficacy and mortality in Parkinson's disease patients treated with subthalamic stimulation. *Movement Disorders: Official Journal of the Movement Disorder Society*. 2011; 26(10):1931–1934.

203. Troche MS, Brandimore AE, Foote KD, i in. Swallowing outcomes following unilateral STN vs. GPi surgery: a retrospective analysis. *Dysphagia*. 2014; 29(4):425–431.

204. Tröster AI, Fields JA, Wilkinson S, i in. Effect of motor improvement on quality of life following subthalamic stimulation is mediated by changes in depressive symptomatology. *Stereotactic and Functional Neurosurgery*. 2003; 80(1-4):43–47.

205. Tsai S-T, Lin S-H, Chou Y-C, i in. Prognostic factors of subthalamic stimulation in Parkinson's disease: a comparative study between short- and long-term effects. *Stereotactic and Functional Neurosurgery*. 2009; 87(4):241–248.

206. Tsai S-T, Lin S-H, Hung H-Y, i in. Long-term comparison of subthalamic nucleus stimulation between patients with young-onset and late-onset Parkinson's disease. *Tzu Chi Medical Journal*. 2012; 24(2):65–72.

207. Tykocki T, Szalecki K, Koziara H, i in. Quality of life and depressive symptoms in Parkinson's disease after subthalamic deep brain stimulation: a 2-year follow-up study. *Turkish Neurosurgery*. 2013; 23(3):379–384.

208. Tykocki T, Nauman P, Koziara H, i in. Microlesion effect as a predictor of the effectiveness of subthalamic deep brain stimulation for Parkinson’s disease. *Stereotactic and Functional Neurosurgery*. 2013; 91(1):12–17.

209. Umemura A, Oka Y, Yamamoto K, i in. Complications of subthalamic nucleus stimulation in Parkinson’s disease. *Neurologia Medico-Chirurgica*. 2011; 51(11):749–755.

210. Vercruysse S, Vandenberghe W, Münks L, i in. Effects of deep brain stimulation of the subthalamic nucleus on freezing of gait in Parkinson’s disease: a prospective controlled study. *Journal of Neurology, Neurosurgery, and Psychiatry*. 2014; 85(8):871–877.

211. Vergani F, Landi A, Pirillo D, i in. Surgical, medical, and hardware adverse events in a series of 141 patients undergoing subthalamic deep brain stimulation for Parkinson disease. *World Neurosurgery*. 2010; 73(4):338–344.

212. Vesper J, Haak S, Ostertag C, i in. Subthalamic nucleus deep brain stimulation in elderly patients--analysis of outcome and complications. *BMC neurology*. 2007; 7:7.

213. Vingerhoets G, van der Linden C, Lannoo E, i in. Cognitive outcome after unilateral pallidal stimulation in Parkinson’s disease. *Journal of Neurology, Neurosurgery, and Psychiatry*. 1999; 66(3):297–304.

214. Vingerhoets FJG, Villemure J-G, Temperli P, i in. Subthalamic DBS replaces levodopa in Parkinson’s disease: two-year follow-up. *Neurology*. 2002; 58(3):396–401.

215. Visser-Vandewalle V, van der Linden C, Temel Y, i in. Long-term motor effect of unilateral pallidal stimulation in 26 patients with advanced Parkinson disease. *Journal of Neurosurgery*. 2003; 99(4):701–707.

216. Visser-Vandewalle V, van der Linden C, Temel Y, i in. Long-term effects of bilateral subthalamic nucleus stimulation in advanced Parkinson disease: a four year follow-up study. *Parkinsonism & Related Disorders*. 2005; 11(3):157–165.

217. Voges J, Waerzeggers Y, Maarouf M, i in. Deep-brain stimulation: long-term analysis of complications caused by hardware and surgery--experiences from a single centre. *Journal of Neurology, Neurosurgery, and Psychiatry*. 2006; 77(7):868–872.

218. Volkmann J, Albanese A, Kulisevsky J, i in. Long-term effects of pallidal or subthalamic deep brain stimulation on quality of life in Parkinson’s disease. *Movement Disorders: Official Journal of the Movement Disorder Society*. 2009; 24(8):1154–1161.

219. Walker HC, Watts RL, Guthrie S, i in. Bilateral effects of unilateral subthalamic deep brain stimulation on Parkinson’s disease at 1 year. *Neurosurgery*. 2009; 65(2):302–309; discussion 309–310.

220. Wang X, Chang C, Geng N, i in. Long-term effects of bilateral deep brain stimulation of the subthalamic nucleus on depression in patients with Parkinson’s disease. *Parkinsonism & Related Disorders*. 2009; 15(8):587–591.

221. Weaver FM, Follett K, Stern M, i in. Bilateral Deep Brain Stimulation vs Best Medical Therapy for Patients With Advanced Parkinson Disease. *JAMA : the journal of the American Medical Association*. 2009; 301(1):63.

222. Weaver FM, Follett KA, Stern M, i in. Randomized trial of deep brain stimulation for Parkinson disease: thirty-six-month outcomes. *Neurology*. 2012; 79(1):55–65.

223. Welter M-L, Schüpbach M, Czernecki V, i in. Optimal target localization for subthalamic stimulation in patients with Parkinson disease. *Neurology*. 2014; 82(15):1352–1361.

224. Wenzelburger R, Kopper F, Zhang B-R, i in. Subthalamic nucleus stimulation for Parkinson’s disease preferentially improves akinesia of proximal arm movements compared to finger movements. *Movement Disorders: Official Journal of the Movement Disorder Society*. 2003; 18(10):1162–1169.

225. Wider C, Russmann H, Villemure J-G, i in. Long-duration response to levodopa in patients with advanced Parkinson disease treated with subthalamic deep brain stimulation. *Archives of Neurology*. 2006; 63(7):951–955.

226. Wider C, Pollo C, Bloch J, i in. Long-term outcome of 50 consecutive Parkinson’s disease patients treated with subthalamic deep brain stimulation. *Parkinsonism & Related Disorders*. 2008; 14(2):114–119.

227. Williams A, Gill S, Varma T, i in. Deep brain stimulation plus best medical therapy versus best medical therapy alone for advanced Parkinson’s disease (PD SURG trial): a randomised, open-label trial. *Lancet Neurology*. 2010; 9(6):581–591.

228. Winkler D, Hammer N, Oehlwein C, i in. Implementing conventional Zamorano Dujovny frames versus individually manufactured microTargeting platforms--a comparative study on deep brain stimulation in Parkinson patients. *Stereotactic and Functional Neurosurgery*. 2013; 91(6):392–398.

229. Witjas T, Kaphan E, Régis J, i in. Effects of chronic subthalamic stimulation on nonmotor fluctuations in Parkinson’s disease. *Movement Disorders: Official Journal of the Movement Disorder Society*. 2007; 22(12):1729–1734.

230. Witt K, Daniels C, Krack P, i in. Negative impact of borderline global cognitive scores on quality of life after subthalamic nucleus stimulation in Parkinson’s disease. *Journal of the Neurological Sciences*. 2011; 310(1-2):261–266.

231. Witt K, Granert O, Daniels C, i in. Relation of lead trajectory and electrode position to neuropsychological outcomes of subthalamic neurostimulation in Parkinson's disease: results from a randomized trial. *Brain: A Journal of Neurology*. 2013; 136(Pt 7):2109–2119.

232. Yamada K, Goto S, Matsuzaki K, i in. Psychiatric symptoms and subthalamic nucleus stimulation in Parkinson's disease. A retrospective study in our Japanese patients. *Neuromodulation: Journal of the International Neuromodulation Society*. 2006; 9(2):107–114.

233. Yamada K, Goto S, Kuratsu J-I, i in. Stereotactic surgery for subthalamic nucleus stimulation under general anesthesia: a retrospective evaluation of Japanese patients with Parkinson's disease. *Parkinsonism & Related Disorders*. 2007; 13(2):101–107.

234. Yamada K, Goto S, Hamasaki T, i in. Effect of bilateral subthalamic nucleus stimulation on levodopa-unresponsive axial symptoms in Parkinson's disease. *Acta Neurochirurgica*. 2008; 150(1):15–22; discussion 22.

235. Yamada K, Hamasaki T, Kuratsu J. Subthalamic nucleus stimulation applied in the earlier vs. advanced stage of Parkinson's disease - retrospective evaluation of postoperative independence in pursuing daily activities. *Parkinsonism & Related Disorders*. 2009; 15(10):746–751.

236. Yamada K, Shiraishi S, Hamasaki T, i in. Cardiac ^{123}I-MIBG scintigraphy as an outcome-predicting tool for subthalamic nucleus stimulation in Parkinson's disease. *Acta Neurochirurgica*. 2010; 152(12):2063–2068.

237. Zahodne LB, Okun MS, Foote KD, i in. Greater improvement in quality of life following unilateral deep brain stimulation surgery in the globus pallidus as compared to the subthalamic nucleus. *Journal of Neurology*. 2009; 256(8):1321–1329.

238. Zangaglia R, Pacchetti C, Pasotti C, i in. Deep brain stimulation and cognitive functions in Parkinson's disease: A three-year controlled study. *Movement Disorders: Official Journal of the Movement Disorder Society*. 2009; 24(11):1621–1628.

239. Zibetti M, Torre E, Cinquepalmi A, i in. Motor and nonmotor symptom follow-up in parkinsonian patients after deep brain stimulation of the subthalamic nucleus. *European Neurology*. 2007; 58(4):218–223.

240. Zibetti M, Pesare M, Cinquepalmi A, i in. Neuro-psychiatric therapy during chronic subthalamic stimulation in Parkinson's disease. *Parkinsonism & Related Disorders*. 2009; 15(2):128–133.

241. International Society For Pharmacoeconomics and Outcomes Research. http://www.ispor.org/ (8.2.2016).

Long-term progression in BMT studies

242. Reinoso G, Allen JC, Au W-L, i in. Clinical evolution of Parkinson's disease and prognostic factors affecting motor progression: 9-year follow-up study. *European Journal of Neurology: The Official Journal of the European Federation of Neurological Societies*. 2015; 22(3):457–463.

243. Jankovic J, Kapadia AS. Functional decline in Parkinson disease. *Archives of Neurology*. 2001; 58(10):1611–1615.

244. Alves G, Wentzel-Larsen T, Aarsland D, i in. Progression of motor impairment and disability in Parkinson disease: a population-based study. *Neurology*. 2005; 65(9):1436–1441.

245. LeWitt PA, Boroojerdi B, Surmann E, i in. Rotigotine transdermal system for long-term treatment of patients with advanced Parkinson's disease: results of two open-label extension studies, CLEOPATRA-PD and PREFER. *Journal of Neural Transmission (Vienna, Austria: 1996)*. 2013; 120(7):1069–1081.

246. Brooks DJ, Leinonen M, Kuoppamäki M, i in. Five-year efficacy and safety of levodopa/DDCI and entacapone in patients with Parkinson's disease. *Journal of Neural Transmission (Vienna, Austria: 1996)*. 2008; 115(6):843–849.

247. Schrag A, Dodel R, Spottke A, i in. Rate of clinical progression in Parkinson's disease. A prospective study. *Movement Disorders: Official Journal of the Movement Disorder Society*. 2007; 22(7):938–945.

248. Antonini A, Barone P, Marconi R, i in. The progression of non-motor symptoms in Parkinson's disease and their contribution to motor disability and quality of life. *Journal of Neurology*. 2012; 259(12):2621–2631.

249. Bloem BR, Grimbergen YA, Cramer M, i in. Prospective assessment of falls in Parkinson's disease. *Journal of Neurology*. 2001; 248(11):950–958.

250. Chan PLS, Nutt JG, Holford NHG. Levodopa slows progression of Parkinson's disease: external validation by clinical trial simulation. *Pharmaceutical Research*. 2007; 24(4):791–802.

251. Cheng K-Y, Lin W-C, Chang W-N, i in. Factors associated with fall-related fractures in Parkinson's disease. *Parkinsonism & Related Disorders*. 2014; 20(1):88–92.

252. Chia LG, Liu LH. Parkinson's disease in Taiwan: an analysis of 215 patients. *Neuroepidemiology*. 1992; 11(3):113–120.

253. Di Rocco A, Molinari SP, Kollmeier B, i in. Parkinson's disease: progression and mortality in the L-DOPA era. *Advances in Neurology*. 1996; 69:3–11.

254. Duarte J, García Olmos LM, Mendoza A, i in. Natural History of Parkinson's Disease in the Province of Segovia: Disability in a 20 Years Longitudinal Study. *Neuro-Degenerative Diseases*. 2015; 15(2):87–92.

255. Evans JR, Mason SL, Williams-Gray CH, i in. The natural history of treated Parkinson's disease in an incident, community based cohort. *Journal of Neurology, Neurosurgery, and Psychiatry*. 2011; 82(10):1112–1118.

256. Factor SA, Molho ES, Feustel PJ, i in. Long-term comparative experience with tolcapone and entacapone in advanced Parkinson's disease. *Clinical Neuropharmacology*. 2001; 24(5):295–299.

257. Foltynie T, Brayne CEG, Robbins TW, i in. The cognitive ability of an incident cohort of Parkinson's patients in the UK. The CamPaIGN study. *Brain: A Journal of Neurology*. 2004; 127(Pt 3):550–560.

258. Gago MF, Garrett MC, Fonseca MR, i in. How do cognitive and axial motor signs correlate in Parkinson's disease? A 6-year prospective study. *Journal of Neurology*. 2009; 256(10):1655–1662.

259. Gazibara T, Pekmezovic T, Kisic Tepavcevic D, i in. Fall frequency and risk factors in patients with Parkinson's disease in Belgrade, Serbia: a cross-sectional study. *Geriatrics & Gerontology International*. 2015; 15(4):472–480.

260. Goetz CG, Tanner CM, Stebbins GT, i in. Risk factors for progression in Parkinson's disease. *Neurology*. 1988; 38(12):1841–1844.

261. Guimaraes P, Kieburtz K, Goetz CG, i in. Non-linearity of Parkinson's disease progression: implications for sample size calculations in clinical trials. *Clinical Trials (London, England)*. 2005; 2(6):509–518.

262. Hauser RA, McDermott MP, Messing S. Factors associated with the development of motor fluctuations and dyskinesias in Parkinson disease. *Archives of Neurology*. 2006; 63(12):1756–1760.

263. Hauser RA, Rascol O, Korczyn AD, i in. Ten-year follow-up of Parkinson's disease patients randomized to initial therapy with ropinirole or levodopa. *Movement Disorders: Official Journal of the Movement Disorder Society*. 2007; 22(16):2409–2417.

264. Hely MA, Morris JG, Traficante R, i in. The sydney multicentre study of Parkinson's disease: progression and mortality at 10 years. *Journal of Neurology, Neurosurgery, and Psychiatry*. 1999; 67(3):300–307.

265. Hely MA, Morris JGL, Reid WGJ, i in. Sydney Multicenter Study of Parkinson's disease: non-L-dopa-responsive problems dominate at 15 years. *Movement Disorders: Official Journal of the Movement Disorder Society*. 2005; 20(2):190–199.

266. Hiorth YH, Lode K, Larsen JP. Frequencies of falls and associated features at different stages of Parkinson's disease. *European Journal of Neurology*. 2013; 20(1):160–166.

267. Hiorth YH, Larsen JP, Lode K, i in. Natural history of falls in a population-based cohort of patients with Parkinson's disease: an 8-year prospective study. *Parkinsonism & Related Disorders*. 2014; 20(10):1059–1064.

268. Maier Hoehn MM. Parkinsonism treated with levodopa: progression and mortality. *Journal of Neural Transmission. Supplementum*. 1983; 19:253–264.

269. Holford NHG, Chan PLS, Nutt JG, i in. Disease progression and pharmacodynamics in Parkinson disease - evidence for functional protection with levodopa and other treatments. *Journal of Pharmacokinetics and Pharmacodynamics*. 2006; 33(3):281–311.

270. Johnson SJ, Diener MD, Kaltenboeck A, i in. An economic model of Parkinson's disease: implications for slowing progression in the United States. *Movement Disorders: Official Journal of the Movement Disorder Society*. 2013; 28(3):319–326.

271. Johnson L, James I, Rodrigues J, i in. Clinical and posturographic correlates of falling in Parkinson's disease: Balance and Posturography in PD. *Movement Disorders*. 2013; 28(9):1250–1256.

272. Klotsche J, Reese JP, Winter Y, i in. Trajectory classes of decline in health-related quality of life in Parkinson's disease: a pilot study. *Value in Health: The Journal of the International Society for Pharmacoeconomics and Outcomes Research*. 2011; 14(2):329–338.

273. Lang AE, Eberly S, Goetz CG, i in. Movement disorder society unified Parkinson disease rating scale experiences in daily living: longitudinal changes and correlation with other assessments. *Movement Disorders: Official Journal of the Movement Disorder Society*. 2013; 28(14):1980–1986.

274. Larsen JP, Boas J, Erdal JE. Does selegiline modify the progression of early Parkinson's disease? Results from a five-year study. The Norwegian-Danish Study Group. *European Journal of Neurology*. 1999; 6(5):539–547.

275. Lindholm B, Hagell P, Hansson O, i in. Prediction of falls and/or near falls in people with mild Parkinson's disease. *PloS One*. 2015; 10(1):e0117018.

276. Liou H-H, Wu C-Y, Chiu Y-H, i in. Natural history and effectiveness of early detection of Parkinson's disease: results from two community-based programmes in Taiwan (KCIS no. 11). *Journal of Evaluation in Clinical Practice*. 2008; 14(2):198–202.

277. López IC, Ruiz PJG, Del Pozo SVF, i in. Motor complications in Parkinson's disease: ten year follow-up study. *Movement Disorders: Official Journal of the Movement Disorder Society*. 2010; 25(16):2735–2739.

278. Matinolli M, Korpelainen JT, Sotaniemi KA, i in. Recurrent falls and mortality in Parkinson's disease: a prospective two-year follow-up study. *Acta Neurologica Scandinavica*. 2011; 123(3):193–200.
279. Müller T, Muhlack S, Woitalla D. Pain perception, pain drug therapy and health status in patients with Parkinson's disease. *Neuroepidemiology*. 2011; 37(3-4):183–187.
280. Pickering RM, Grimbergen YAM, Rigney U, i in. A meta-analysis of six prospective studies of falling in Parkinson's disease. *Movement Disorders: Official Journal of the Movement Disorder Society*. 2007; 22(13):1892–1900.
281. Poewe W. The natural history of Parkinson's disease. *Journal of Neurology*. 2006; 253 Suppl 7:VII2–6.
282. Rajput AH, Voll A, Rajput ML, i in. Course in Parkinson disease subtypes: A 39-year clinicopathologic study. *Neurology*. 2009; 73(3):206–212.
283. Rascol O, Brooks DJ, Korczyn AD, i in. A five-year study of the incidence of dyskinesia in patients with early Parkinson's disease who were treated with ropinirole or levodopa. 056 Study Group. *The New England Journal of Medicine*. 2000; 342(20):1484–1491.
284. Roos RA, Jongen JC, van der Velde EA. Clinical course of patients with idiopathic Parkinson's disease. *Movement Disorders: Official Journal of the Movement Disorder Society*. 1996; 11(3):236–242.
285. Rudzińska M, Bukowczan S, Banaszkiewicz K, i in. Causes and risk factors of falls in patients with Parkinson's disease. *Neurologia I Neurochirurgia Polska*. 2008; 42(3):216–222.
286. Rudzińska M, Bukowczan S, Stożek J, i in. The incidence and risk factors of falls in Parkinson disease: prospective study. *Neurologia I Neurochirurgia Polska*. 2013; 47(5):431–437.
287. Rudzińska M, Bukowczan S, Stożek J, i in. Causes and consequences of falls in Parkinson disease patients in a prospective study. *Neurologia I Neurochirurgia Polska*. 2013; 47(5):423–430.
288. Sato K, Hatano T, Yamashiro K, i in. Prognosis of Parkinson's disease: time to stage III, IV, V, and to motor fluctuations. *Movement Disorders: Official Journal of the Movement Disorder Society*. 2006; 21(9):1384–1395.
289. Schrag A, Jahanshahi M, Quinn N. How does Parkinson's disease affect quality of life? A comparison with quality of life in the general population. *Movement Disorders: Official Journal of the Movement Disorder Society*. 2000; 15(6):1112–1118.
290. Schrag A, Spottke A, Quinn NP, i in. Comparative responsiveness of Parkinson's disease scales to change over time. *Movement Disorders: Official Journal of the Movement Disorder Society*. 2009; 24(6):813–818.
291. Stocchi F, Ruggieri S, Vacca L, i in. Prospective randomized trial of lisuride infusion versus oral levodopa in patients with Parkinson's disease. *Brain: A Journal of Neurology*. 2002; 125(Pt 9):2058–2066.
292. Velseboer DC, Broeders M, Post B, i in. Prognostic factors of motor impairment, disability, and quality of life in newly diagnosed PD. *Neurology*. 2013; 80(7):627–633.
293. Vu TC, Nutt JG, Holford NHG. Disease progress and response to treatment as predictors of survival, disability, cognitive impairment and depression in Parkinson's disease. *British Journal of Clinical Pharmacology*. 2012; 74(2):284–295.
294. Williams-Gray CH, Mason SL, Evans JR, i in. The CamPaIGN study of Parkinson's disease: 10-year outlook in an incident population-based cohort. *Journal of Neurology, Neurosurgery, and Psychiatry*. 2013; 84(11):1258–1264.
295. Zhao YJ, Wee HL, Chan Y-H, i in. Progression of Parkinson's disease as evaluated by Hoehn and Yahr stage transition times. *Movement Disorders: Official Journal of the Movement Disorder Society*. 2010; 25(6):710–716.
296. Zhao YJ, Wee HL, Au WL, i in. Selegiline use is associated with a slower progression in early Parkinson's disease as evaluated by Hoehn and Yahr Stage transition times. *Parkinsonism & Related Disorders*. 2011; 17(3):194–197.

Other

297. Xu J, Gong DD, Man CF, i in. Parkinson's disease and risk of mortality: meta-analysis and systematic review. *Acta Neurologica Scandinavica*. 2014; 129(2):71–79.
298. Macleod AD, Taylor KSM, Counsell CE. Mortality in Parkinson's disease: a systematic review and meta-analysis. *Movement Disorders: Official Journal of the Movement Disorder Society*. 2014; 29(13):1615–1622.
299. M. Skorvanek, J. Rosenberger, I. Rajnicova-Nagyova. Fatigue is not independent predictor of mortality in Parkinson's disease.
300. Marras C, McDermott MP, Rochon PA, i in. Survival in Parkinson disease: thirteen-year follow-up of the DATATOP cohort. *Neurology*. 2005; 64(1):87–93.
301. Forsaa EB, Larsen JP, Wentzel-Larsen T, i in. What predicts mortality in Parkinson disease?: a prospective population-based long-term study. *Neurology*. 2010; 75(14):1270–1276.

302. Allyson Jones C, Wayne Martin WR, Wieler M, i in. Incidence and mortality of Parkinson's disease in older Canadians. *Parkinsonism & Related Disorders*. 2012; 18(4):327–331.

303. Auyeung M, Tsoi TH, Mok V, i in. Ten year survival and outcomes in a prospective cohort of new onset Chinese Parkinson's disease patients. *Journal of Neurology, Neurosurgery, and Psychiatry*. 2012; 83(6):607–611.

304. Benito-León J, Louis ED, Villarejo-Galende A, i in. Under-reporting of Parkinson's disease on death certificates: a population-based study (NEDICES). *Journal of the Neurological Sciences*. 2014; 347(1-2):188–192.

305. Chillag-Talmor O, Giladi N, Linn S, i in. Estimation of Parkinson's disease survival in Israeli men and women, using health maintenance organization pharmacy data in a unique approach. *Journal of Neurology*. 2013; 260(1):62–70.

306. Désesquelles A, Demuru E, Salvatore MA, i in. Mortality from Alzheimer's disease, Parkinson's disease, and dementias in France and Italy: a comparison using the multiple cause-of-death approach. *Journal of Aging and Health*. 2014; 26(2):283–315.

307. Duarte J, García Olmos LM, Mendoza A, i in. The natural history of Parkinson's disease in the province of Segovia: mortality in a longitudinal study (20-year follow-up). *Acta Neurologica Scandinavica*. 2013; 127(5):295–300.

308. Frandsen R, Baandrup L, Kjellberg J, i in. Increased all-cause mortality with psychotropic medication in Parkinson's disease and controls: a national register-based study. *Parkinsonism & Related Disorders*. 2014; 20(11):1124–1128.

309. Hobson P, Meara J, Ishihara-Paul L. The estimated life expectancy in a community cohort of Parkinson's disease patients with and without dementia, compared with the UK population. *Journal of Neurology, Neurosurgery, and Psychiatry*. 2010; 81(10):1093–1098.

310. Hoyert DL, Xu J. Deaths: preliminary data for 2011. *National Vital Statistics Reports: From the Centers for Disease Control and Prevention, National Center for Health Statistics, National Vital Statistics System*. 2012; 61(6):1–51.

311. Kaltenboeck A, Johnson SJ, Davis MR, i in. Direct costs and survival of medicare beneficiaries with early and advanced Parkinson's disease. *Parkinsonism & Related Disorders*. 2012; 18(4):321–326.

312. Mackenbach JP, Karanikolos M, Looman CWN. The rise of mortality from mental and neurological diseases in Europe, 1979-2009: observational study. *BMC public health*. 2014; 14:840.

313. Morgan JC, Currie LJ, Harrison MB, i in. Mortality in levodopa-treated Parkinson's disease. *Parkinson's Disease*. 2014; 2014:426976.

314. Oosterveld LP, Allen JC, Reinoso G, i in. Prognostic factors for early mortality in Parkinson's disease. *Parkinsonism & Related Disorders*. 2015; 21(3):226–230.

315. Peretz C, Chillag-Talmor O, Linn S, i in. Parkinson's disease patients first treated at age 75 years or older: a comparative study. *Parkinsonism & Related Disorders*. 2014; 20(1):69–74.

316. Willis AW, Schootman M, Kung N, i in. Predictors of survival in patients with Parkinson disease. *Archives of Neurology*. 2012; 69(5):601–607.

317. Palmer CS, Nuijten MJC, Schmier JK, i in. Cost effectiveness of treatment of Parkinson's disease with entacapone in the United States. *PharmacoEconomics*. 2002; 20(9):617–628.

318. Lowin J, Bergman A, Chaudhuri KR, i in. A cost-effectiveness analysis of levodopa/carbidopa intestinal gel compared to standard care in late stage Parkinson's disease in the UK. *Journal of Medical Economics*. 2011; 14(5):584–593.

319. Walter E, Odin P. Cost-effectiveness of continuous subcutaneous apomorphine in the treatment of Parkinson's disease in the UK and Germany. *Journal of Medical Economics*. 2015; 18(2):155–165.

320. Nyholm D, Klangemo K, Johansson A. Levodopa/carbidopa intestinal gel infusion long-term therapy in advanced Parkinson's disease: Long-term levodopa gel infusion. *European Journal of Neurology*. 2012; 19(8):1079–1085.

321. Kataoka H, Tanaka N, Eng M, i in. Risk of falling in Parkinson's disease at the Hoehn-Yahr stage III. *European Neurology*. 2011; 66(5):298–304.

322. Voss TS, Elm JJ, Wielinski CL, i in. Fall frequency and risk assessment in early Parkinson's disease. *Parkinsonism & Related Disorders*. 2012; 18(7):837–841.

323. Schuepbach WMM, Rau J, Knudsen K, i in. Neurostimulation for Parkinson's disease with early motor complications. *The New England Journal of Medicine*. 2013; 368(7):610–622.

324. Murphy AM. Economic evaluations for health technologies with an evolving evidence base: a case study of transcatheter aortic valve implantation. University of Glasgow http://theses.gla.ac.uk/4061/ (21.12.2015).
