# Supplementary material for: Deep Brain Stimulation for Parkinson’s Disease with Early Motor Complications: A UK Cost-Effectiveness Analysis
Source: PLoS One. 2016 Jul 21;11(7):e0159340. doi: 10.1371/journal.pone.0159340 (PMC4956248; doi:10.1371/journal.pone.0159340)
Supplement: S1 Table — (DOCX) [file pone.0159340.s003.docx]

**S1 Table: Parameter input values, sources and distributions for probabilistic sensitivity analysis**

| **Parameter** | **Deterministic value (standard deviation)** | **Distribution in PSA** | **Source** |
| --- | --- | --- | --- |
| **Baseline/Demographic Parameters (all treatments)** | | | |
| Age at baseline | 52.55 (0.4) | Normal | [36] |
| % of male patients | 71.3% (0.9%) | Beta | [36] |
| UPDRS I score | 1.06 (0.17) | Normal | [36] |
| UPDRS II score | 4.85 (0.57) | Normal | [36] |
| UPDRS III score | 12.29 (1.48) | Normal | [36] |
| UPDRS IV score | 5.54 (0.31) | Normal | [36] |
| **2-year efficacy parameters – % change in UPDRS scores versus baseline** | | | |
| BMT:  UPDRS I (after treatment initiation)  UPDRS I (1 year)  UPDRS I (2 years)  UPDRS II (after treatment initiation)  UPDRS II (1 year)  UPDRS II (2 years)  UPDRS III (after treatment initiation)  UPDRS III (1 year)  UPDRS III (2 years)  UPDRS IV (after treatment initiation)  UPDRS IV (1 year)  UPDRS IV (2 years) | 0%  25.71% (2.1%)  44.76% (2.26%)  0%  3.92% (1.51%)  11.75% (1.56%)  0%  31.65% (1.56%)  31.65% (1.56%)  0%  -1.63% (0.7%)  12.89% (0.73%) | -  Normal  Normal  -  Normal  Normal  -  Normal  Normal  -  Normal  Normal | Assumption  [36]  Assumption  [36]  [36]  Assumption  [36]  Assumption  Assumption  [36]  [36] |
| DBS:  UPDRS I (after treatment initiation)  UPDRS I (1 year)  UPDRS I (2 years)  UPDRS II (after treatment initiation)  UPDRS II (1 year)  UPDRS II (2 years)  UPDRS III (after treatment initiation)  UPDRS III (1 year)  UPDRS III (2 years)  UPDRS IV (after treatment initiation)  UPDRS IV (1 year)  UPDRS IV (2 years) | 0%  5.61% (2.11%)  15.89% (2.24%)  0%  -9.26% (1.57%)  2.67% (1.58%)  0%  -9.05% (1.54%)  -9.05% (1.54%)  -67.38% (0.7%)  -67.56% (0.71%)  -60.22% (0.73%) | -  Normal  Normal  -  Normal  Normal  -  Normal  Normal  Normal  Normal  Normal | Assumption  [36]  [36]  Assumption  [36]  [36]  Assumption  [36]  Assumption  [36]  [36]  [36] |
| **Annual progression in UPDRS domain scores after year 2 – by treatment** | | | |
| UPDRS I (both treatment groups) | 0.201 (0.02) | Normal | [36,42] |
| UPDRS II (both treatment groups) | 1.136 (0.11) | Normal | [5,36,42,43] |
| UPDRS III (both treatment groups) | 2.425 (0.59) | Normal | [45] |
| UPDRS IV (BMT) – from year 3 onwards | 0.355 (0.22) | Normal | [36] |
| UPDRS IV (DBS) – from year 3 to year 10 | 0 | - | Assumption |
| UPDRS IV (DBS) – from year 10 onwards | 0.355 (0.22) | Normal | [36] |
| **Adverse event probabilities – DBS** | | | |
| Device-related SAEs – per year | 0.0204 (0.0011) | Beta | [36] |
| Surgery-related SAEs (no withdrawal) – year 1 and year 2 | 0.0496 (0.0018) | Beta | [36] |
| Surgery-related SAEs (no withdrawal) – each subsequent year | 0 | - | Assumption |
| Surgery-related SAEs (treatment withdrawal) – year 1 and year 2 | 0.0081 (0.0007) | Beta | [36] |
| Surgery-related SAEs (treatment withdrawal) – each subsequent year | 0 | - | Assumption |
| Worsening of mobility – per year | 0.0204 (0.0011) | Beta | [36] |
| **Adverse event probabilities – BMT** | | | |
| Worsening of mobility – per year | 0.0443 (0.0016) | Beta | [36] |
| Motor fluctuations – per year | 0.0279 (0.0013) | Beta | [36] |
| **Incidence of falls and associated hospitalisation rate** | | | |
| Percentage of patients falling per year (baseline value based on patients with UPDRS III score of 12 points) | 42.78% (0. 92%) | Beta | [61-75] |
| Odds ratio for risk of fall – per 1-point increase in UPDRS III score | 1.067 (0.031) | Lognormal | [61,64,66] |
| Annual number of falls per faller | 1 | - | Assumption |
| Probability that a fall leads to hospitalisation | 0.5088 (0.0248) | Beta | [62,65,72] |
| **Additional days per year in hospital (due to comorbidities)** | | | |
| BMT patients  DBS patients | 5.97 (0.60)  2.63 (0.26) | Normal  Normal | [87]  [87] |
| **Treatment withdrawal probabilities** | | | |
| BMT withdrawal | 0 | - | Assumption |
| DBS withdrawal | 0 | - | Assumption |
| **Mortality parameters** | | | |
| Baseline annual mortality risk | Age- and gender-specific | - | [53] |
| Hazard ratio for mortality risk – per 10-point increase in UPDRS III score above 15 | 1.31 (0.094) | Lognormal | [58,59,60] |
| **Cost parameters – DBS** | | | |
| DBS system: Activa PC implantable pulse generator, device extensions, leads and patient programmer | £12,336 | - | [84] |
| Pre-operative assessment / hospitalisation | £482 | - | [81] HRG code = AA25B |
| Additional pre-operative hospitalisation | £482 | - | [81] HRG code = AA25B |
| DBS implantation procedure | £5,358 | - | [81] HRG code = AA07B |
| Battery replacement procedure | £3,556 | - | [81] HRG code = AB07Z |
| Cost per day of BMT when on DBS | £2.28 (45% reduction vs. BMT group, with SD of 12%) | Normal (% reduction vs. BMT group) | [82,87] |
| Device-related serious adverse event | £6,178.83 (617.88) | Gamma | [81] Multiple HRG codes |
| Surgery-related serious adverse event (no treatment withdrawal) | £4,099.10 (409.91) | Gamma | [81] Multiple HRG codes |
| Surgery-related serious adverse event (with treatment withdrawal) | £4,444.75 | - | [81] Multiple HRG codes |
| Worsening of mobility (cost per day; duration = 8 days) | £570 | - | [86] |
| **Cost parameters – BMT** | | | |
| Worsening of mobility or motor fluctuations – cost per day | £570 (57) | Gamma | [86] |
| Duration of worsening of mobility (days) | 8 | - | [36] |
| Duration of motor fluctuations (days) | 11 | - | [36] |
| Cost per day of BMT when receiving no other therapy | £4.16 (0.48) | Gamma | [82,87] |
| **Cost parameters – miscellaneous** | | | |
| Neurology follow-up visit (outpatient) | £216 | - | [81] Neurology outpatient visit |
| PD nurse visit | £116 | - | [83] |
| Fall-related hospitalisation | £711 (71) | Gamma | [81] HRG code = WA23X |
| **Utility Parameters** | | | |
| DBS utility – year 1 | 0.69 (0.012) | Normal | [36,76] |
| DBS utility – year 2 | 0.68 (0.013) | Normal | [36,76] |
| BMT utility – year 1 | 0.59 (0.013) | Normal | [36,76] |
| BMT utility – year 2 | 0.59 (0.014) | Normal | [36,76] |
| Utilities for both treatment groups treatment groups from year 3 onwards. | Mapping function based on time-dependent UPDRS domain scores | Normal | See quality of life section of main manuscript |
| Utility with device- and surgery-related adverse events (DBS), based on minimum UK EQ-5D utility. | -0.594 | - | Assumption |
| **Miscellaneous Parameters** | | | |
| Discount rate for costs and QALYs | 3.5% per year | - | [40] |
| Battery longevity - DBS | 4.5 years | - | Medtronic device performance data |
